# Decoupling Cyanide Activation from C–C Bond Formation in Ni-Catalyzed Cyanation of Strained Ketones Using Benzonitriles

**DOI:** 10.1021/jacs.5c21546

**Published:** 2026-04-02

**Authors:** Nathan J. Coddington, Robert D. Bradley, Yvette A. Luna, Madison D. Loper, Paul J. Saucedo, Rihan Ouyang, William L. Lo, Veronica Carta, Ana Bahamonde

**Affiliations:** Department of Chemistry, 8790 University of California, Riverside, California 92521, United States

## Abstract

We have developed a Ni-catalyzed approach that generates silyl cyanides in situ to enable C–CN bond formation from readily available, low-toxicity benzonitriles. This strategy decouples cyanide activation from subsequent bond-forming reactions within distinct catalytic cycles to facilitate the ring-opening cyanation of cyclopropyl and cyclobutyl ketones. By separating C–CN activation from coupling, the chemistry is not limited by the intrinsic reactivity of the specific intermediate generated from activating the cyanide precursor. Indeed, we demonstrate that the separation of both cycles allows us to expand Ni-catalyzed cyanation chemistry using nontoxic precursors beyond hydrocyanation of π-systems and benzonitrile synthesis. Specifically, we report the synthesis of γ- and δ-cyanated ketones with broad functional group tolerance. Mechanistic investigations, including kinetic studies, stoichiometric reactions, and the isolation of a rare bimetallic Ni species featuring a bridging cyano ligand, collectively support a pathway in which Ni-catalyzed formation of TMSCN occurs concurrently with Ni-mediated activation of the cyclopropyl and cyclobutyl ketones. This work provides a safer and more sustainable alternative to conventional toxic cyanation reagents and presents a complementary reactivity scope to other nontoxic cyanide protocols where cyanide activation and coupling occur within the same cycle.

## Graphical abstract

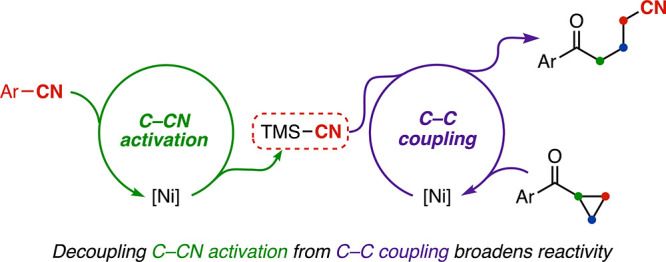

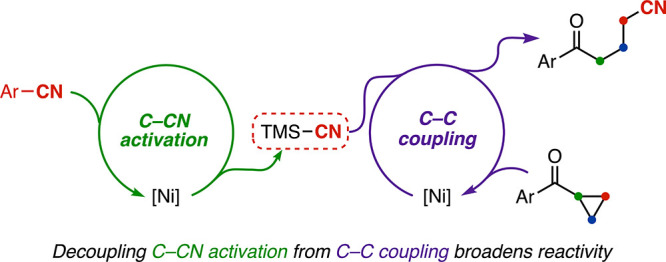

## Introduction

Nitriles are ubiquitous in pharmaceuticals, materials, fine chemicals, and agrochemicals, making efficient and safe cyanation methods of enduring synthetic importance.^1–3^ To mitigate the hazards of using toxic hydrogen cyanide (HCN), a variety of surrogates have been developed. Early examples include inorganic cyanide salts such as KCN and [Fe­(CN)_6_]^4–^, which, although easier to handle, suffer from solubility issues and still present significant toxicity concerns due to their propensity to release HCN under acidic conditions. To improve ease of use while maintaining the high reactivity of HCN, trimethylsilyl cyanide (TMSCN) has emerged as the most widely used alternative.^4–10^ The high solubility of TMSCN allows for it to directly participate in transition-metal-catalyzed cyanation reactions, either as a cyanide donor or through in situ generation of HCN under mild conditions. Despite its advantages, TMSCN is a volatile liquid that remains highly toxic and relatively expensive, limiting its practicality on larger or industrial scales. Given the broad synthetic importance of the cyano group, the development of safer and more sustainable strategies to achieve comparable reactivity remains a compelling goal.

In this vein, most efforts toward milder and safer cyanation chemistry have focused on identifying conditions that enable the formation of metal–cyanide intermediates from lower-toxicity precursors.^11–15^ Morandi and co-workers demonstrated that butyronitrile can serve as a practical cyanide source for the hydrocyanation of olefins at room temperature, proceeding via the formation of a Ni–hydrocyanide intermediate (Figure 1a).^16^ Additionally, the Rousseaux group reported that malononitriles can act as effective cyanide-transfer reagents, where a migratory insertion followed by β-elimination into one of the cyano groups is proposed to drive the transformation (Figure 1b).^17–19^

**Figure 1:**
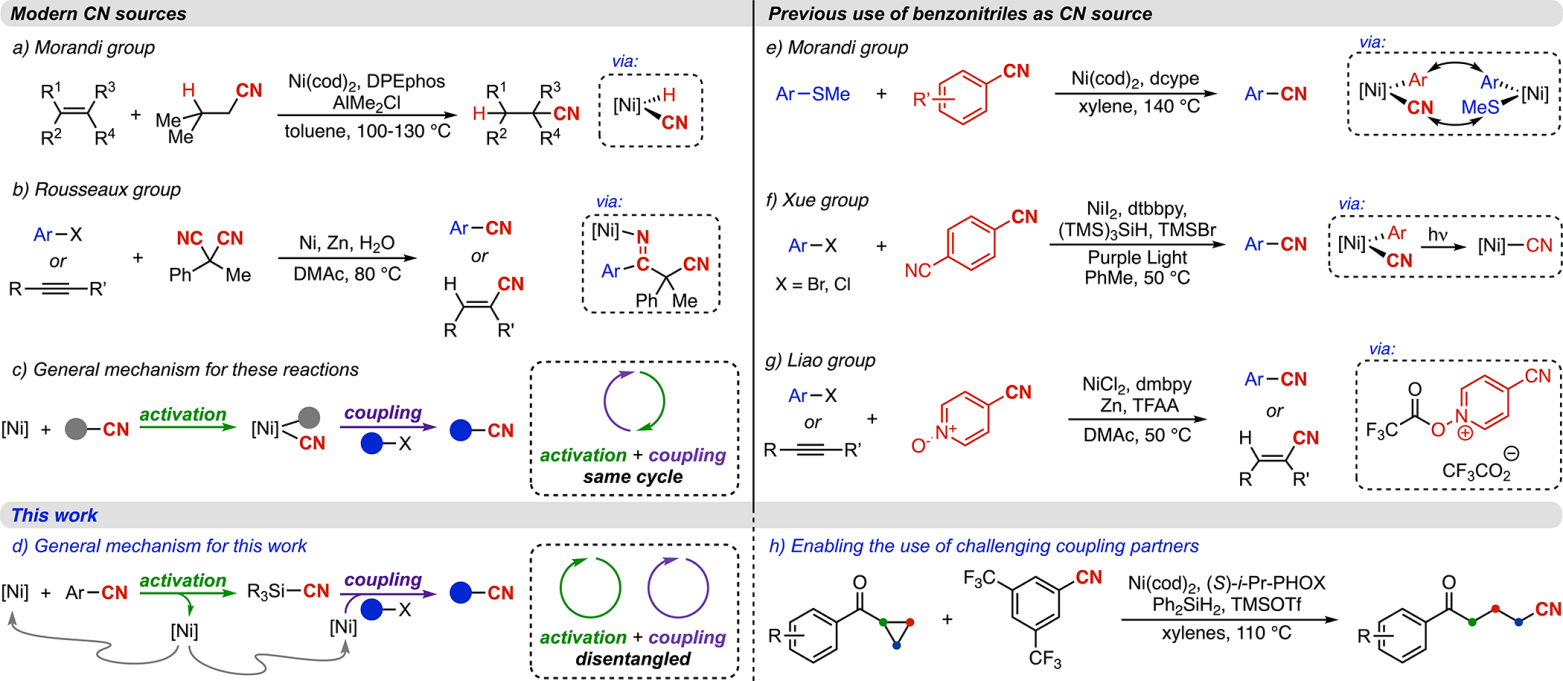
Relevant precedents and this work.

All of these strategies require the formation of specific Ni-ligated CN activation intermediates that are directly involved in the subsequent coupling reactions (Figure 1a,1b).^12–19^ Therefore, these methodologies proceed via mechanisms where the activation of the cyanide precursor and coupling are successive steps in the same catalytic cycle (Figure 1c). This has inherently restricted the accessible reactivity to relatively straightforward transformations; namely, the transfer of CN to an arene and hydrocyanation across π-systems. This contrasts with the broader range of transformations enabled by less reactive, yet more versatile, reagents like TMSCN. Herein, we report conditions that allow the in situ formation of silyl cyanides, akin to TMSCN, directly from nontoxic benzonitriles (Figure 1d). This approach therefore decouples cyanide generation from the subsequent bond-forming step, enabling us to target new transformations, while also minimizing the formation of polycyanated Ni species, a common deactivation pathway in related reactions.

Benzonitriles were chosen as cyanide sources due to their wide availability, reduced toxicity, and the presence of an sp^2^-hybridized carbon cyanide bond.^20,21^ This structural feature lowers the C–CN oxidative addition barrier relative to that for acetonitrile, allowing this step to proceed at room temperature instead of the high temperatures (>150 °C) typically required for acetonitrile.^22–24^ The activation of benzonitriles by low-valent Ni species has been known since 1974,^25,26^ and their primary use has been as an aryl electrophile surrogate in cross-coupling reactions, where the cyanide is discarded and only functions as a leaving group.^27–35^ In comparison, routes that exploit benzonitriles specifically for cyanation are significantly rarer. This reactivity is limited to two examples reported by the Morandi^36^ and Xue^37^ groups, both of which describe the transfer of cyanide from one arene to another (Figure 1e,f, respectively).

Morandi achieved benzonitrile formation through a functional-group metathesis between a thioether and a benzonitrile, whereas Xue accessed benzonitrile products via purple-light photolysis of a Ni–aryl cyanide intermediate. Related reactivity has also been demonstrated using cyanopyridine *N*-oxide reagents, which under Ni catalysis and activation by an anhydride can also generate new benzonitriles as well as facilitate the hydrocyanation of alkynes (Figure 1g).^38^ Once again, in contrast to our study, the Ni–CN bond formed during activation of the aryl cyanide directly engages in the coupling event in all of these examples (Figure 1c). This therefore prevents the activation and bond-forming steps from being disentangled (Figure 1d), which we envision to be a critical design element for integrating cyanide activation with the wider scope of reactivity typically afforded by Ni catalysis.

To demonstrate the utility of in situ generation of silyl cyanides from benzonitrile, we sought to evaluate a demanding class of coupling partners and selected cyclopropyl ketones, which require a Ni-catalyzed cleavage of an additional C–C bond (Figure 1h).^39–42^ In the context of Ni catalysis, these strained systems have previously been coupled to π-systems to furnish five-membered rings^43^ and have been intercepted by diverse coupling partners to deliver ring-opened alkylation,^44–46^ arylation,^47^ and borylation^48^ products. To our surprise, the reactivity that we describe in this paper surpasses these previous examples by also accommodating cyclobutyl ketone, which is, to the best of our knowledge, unprecedented in Ni-catalyzed C–C bond activation. The Ni activation of cyclobutyl groups has been restricted to highly activated systems like biphenylene, cyclobutanone, and cyclobutenone.^49–51^ Furthermore, this reactivity enables access to γ- and δ-cyanated ketones from versatile three- and four-carbon synthons.

It should be noted that the ring opening and cyanation of cyclopropyl ketones has been reported using a Cu-catalyzed photochemical approach that proceeds via open-shell intermediates using TMSCN.^52^ However, beyond the safety concerns of using TMSCN as a reagent, the two-electron Ni chemistry described here provides access to completely orthogonal products, with no overlap in the scope between both methodologies. Specifically, their approach is limited to the use of cyclopropyl rings that render a resonance-stabilized radical upon opening to yield benzylic, allylic, and propargylic cyanides. This is not a requirement for our chemistry, which operates via an entirely different mechanism (vide infra).

## Results and Discussion

For simplicity, phenyl cyclopropyl ketone **1a** was selected as the model substrate for the initial optimization campaign (Table 1). Preliminary ligand screening revealed that phosphine ligands delivered superior yields, with diphenylphosphino oxazoline ligands (PHOX) and 1,2-bis­(diphenylphosphino)­ethane (dppe) performing best and *i*-Pr-PHOX providing the highest yields (see Figure S1).

**Table 1:**
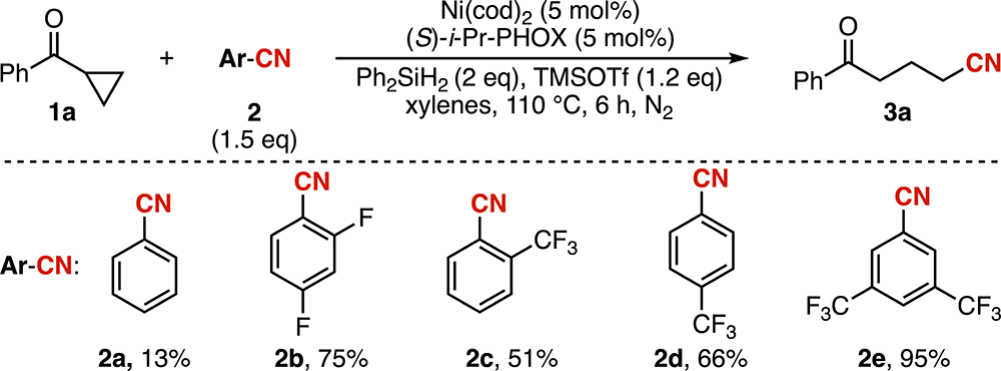
Reaction Optimization and Control Reactions^a^

entry	Ar-*CN*	other variations	yield of **3a**
1	**2e**	None	95%
2	**2e**	Ni(cod)_2_ (10 mol%)	80%
3	**2e**	100 °C	92%
4	**2e**	No Ph_2_SiH_2_	6%
5	**2e**	No TMSOTf	not detected
6	**2e**	No Ni(cod)_2_	not detected

a Reaction conditions: Ni­(cod)_2_ (0.0075 mmol), (*S*)-*i*-Pr-PHOX (0.0075 mmol), **1a** (0.15 mmol), **2** (0.225 mmol), diphenyl silane (0.3 mmol), TMSOTf (0.18 mmol), and xylenes (2.25 mL). The solution was heated to 110 °C for 6 h. All yields were determined by ^1^H NMR using dimethyl fumarate as an internal standard.

A series of benzonitriles was then evaluated, and electron-poor arenes provided the highest yields (**2a**–**2e**). With **2e** identified as the optimal cyanide source, we determined that the best catalytic system consisted of 1.2 equiv of trimethylsilyl trifluoromethanesulfonate (TMSOTf) and 2 equiv of diphenylsilane, heated at 110 °C in xylenes for 6 h (entry 1). Similar reactivity was observed for 5 and 10 mol% Ni loadings, with different optimal loadings depending on the substrate (Table 1, entries 1–2 and Figure 2). Lowering the reaction temperature to 100 °C resulted in a modest reduction of the product yield (entry 3).

**Figure 2:**
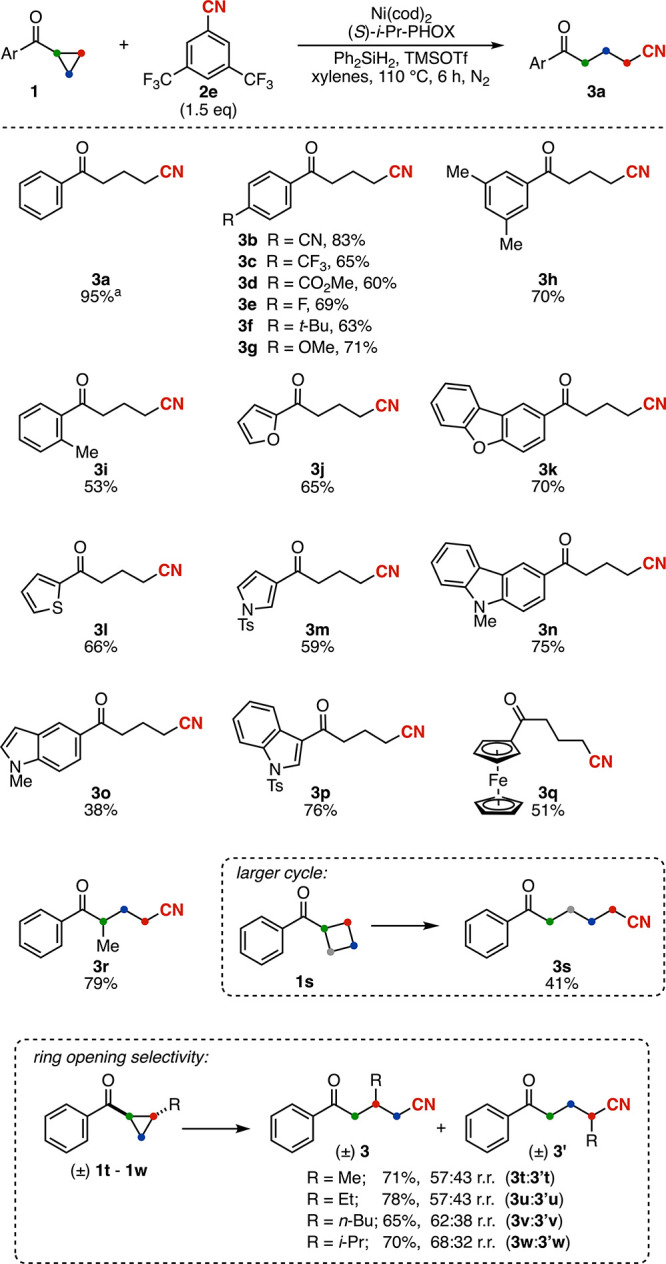
Cyclopropyl and cyclobutyl ketone cyanation scope. Reaction conditions: Ni­(cod)_2_ (0.015 mmol), *(S)-i*-Pr-PHOX (0.015 mmol), **1** (0.15 mmol), **2e** (0.225 mmol), diphenyl silane (0.3 mmol), TMSOTf (0.18 mmol), and xylenes (2.25 mL). The solution was heated to 110 °C for 6 h. All yields were determined by isolation. ^a^ 5 mol% nickel and ligand was employed.

When running control reactions to test the necessity of each component, small amounts of product were observed in the absence of silane (entry 4). The yields, however, plateau at levels matching the Ni loading, suggesting that silane is required for catalyst turnover but is not otherwise involved in the catalytic cycle. This need for a reductant is unsurprising, as the reaction couples two electrophiles. In contrast, both the TMSOTf Lewis acid and Ni were required to form product (entries 5 and 6).

With the optimized conditions in hand, we examined the scope of the reaction. Evaluation of the electronic properties of the arene revealed that a range of *para*-substituted ketones were well accommodated, with electron-withdrawing groups generally providing higher yields than electron-donating substituents (**3a**–**3g**). Notably, Lewis-basic functionalities such as the ester in **3d** or the methoxy group in **3g** led to only modest decreases in yield. We were also pleased to observe that selective activation of benzonitrile **2e** can be obtained in the presence of other benzonitrile functionality, and no decyanation was observed in the formation of **3b**.

Different patterns of aromatic substitution were also well tolerated, including *ortho* substitution, which resulted in only a modest decrease in efficiency (**3h** and **3i**). The reaction is also compatible with a variety of heterocycles, which furnished **3j**–**3q** in modest to good yields. Indole **1o** was identified as an exception, leading to a low yield of the cyanated product **3o**, along with complete consumption of the starting indole to form unidentified side products. These results are notable given the propensity of such substrates to coordinate Ni or to engage in competing reactions with the Lewis acid. In contrast, substrates bearing pyridine substituents were identified as limitations of this methodology (Figure S2).

Beyond aryl and heteroaryl variations, changes to the cyclopropyl core were also examined. A ketone containing an α-quaternary center was shown to perform well, delivering **3r** in 79% yield. Beyond its synthetic value, this result indicates that enolization of the cyclopropyl ketone is not required for productive ring opening, as this pathway is not accessible for this substrate.

To our delight, cyclobutyl ketone **1s** also furnished the ring-opening cyanated product **3s** in 41% yield. This result is particularly notable, as cyclobutyl ketones rarely engage in Ni-catalyzed ring-opening processes. Indeed, the ring opening of unfunctionalized cyclobutyl ketones, which endure a reduced ring strain, are unprecedented for first row transition metals.^53^ Moreover, the success of this substrate supports our proposal that oxidative cyclization does not proceed through single-electron transfer followed by radical ring opening, as has been invoked in related systems.^48,52^

Cyclopropanes bearing substituents on the ring (**1t**–**1w**) may open between the α-carbon (highlighted in green) and the substituted or unsubstituted carbon (highlighted in red and blue, respectively), leading to cyanation at either the substituted or unsubstituted carbon. The primary, less hindered cyanide is favored under these conditions. Smaller substituents like methyl and ethyl groups give a regioisomeric ratio (r.r.) of 57:43 (**3t** and **3u**). Increasing the steric bulk of the alkyl substituent leads to an increase in the r.r. to 62:38 and 68:23 for **3v** and **3w**, respectively; however, it does not appear to influence the yield. Although the selectivity levels are moderate, reaction at the less substituted carbon is preferred. This pattern is inconsistent with an open-shell oxidative addition mechanism, which would favor functionalization at the more substituted site. Finally, despite using an enantiopure ligand, no chiral induction was detected in the formation of these products.

In contrast to the aliphatic substrates, cyclopropyl ketones bearing aryl groups (R = aryl) preferentially underwent formation of the corresponding β,γ-unsaturated ketones (Figure S2).^54^ This divergence in reactivity highlights the complementarity of our approach to the reported Cu-mediated protocol, which is largely restricted to aryl-substituted cyclopropyl ketones.^52^

### Mechanistic Investigations

Previous studies have shown that Ni(0) species can undergo Lewis acid–assisted oxidative addition into benzonitriles^55–58^ as well as cyclopropyl ketones,^40,43,59^ affording Ni–aryl cyanides and Ni-bound enolates, respectively. These precedents led us to consider whether these intermediates might engage in a transmetalation step to generate the observed cyanated product.

To evaluate this possibility, we probed the effect of Ni concentration on the reaction rate. A bimolecular step involving two Ni catalytic intermediates would be expected to be rate-limiting, and thus would exhibit a second-order dependence on Ni.^60,61^ Contrary to this expectation, our kinetic measurements revealed saturation behavior, with an apparent Ni order of approximately 0.6 under the reaction conditions (see Figures S3–S6). This data does not definitively rule out a possible transmetalation-mediated mechanism, but it suggests that other mechanistic pathways may be predominant and prompted us to further investigate alternative mechanistic hypotheses.

For Ni- and Rh-catalyzed reduction of benzonitriles, the Maiti and Bergman/Brookhart groups, respectively, demonstrated that, in the presence of a silane, metal–aryl cyanide complexes undergo formation of a silyl isocyanide.^62–64^ Because silyl isocyanides are in rapid equilibrium with silyl cyanides,^65^ we sought to test whether such an intermediate could be involved in the mechanism of our reaction. To this end, we conducted a reaction in which benzonitrile was replaced by TMSCN, which afforded the desired product in comparable yield to the standard conditions (99% vs 95%; Figure 3a). Given our initial observation that the silane is only required for Ni turnover (Table 1, entry 4), we also performed this experiment with TMSCN in the absence of silane, and the product was still formed in 92% yield. This indicates that TMSCN is a suitable CN source for the reaction that can enable multiple turnovers in the absence of the silane.

**Figure 3:**
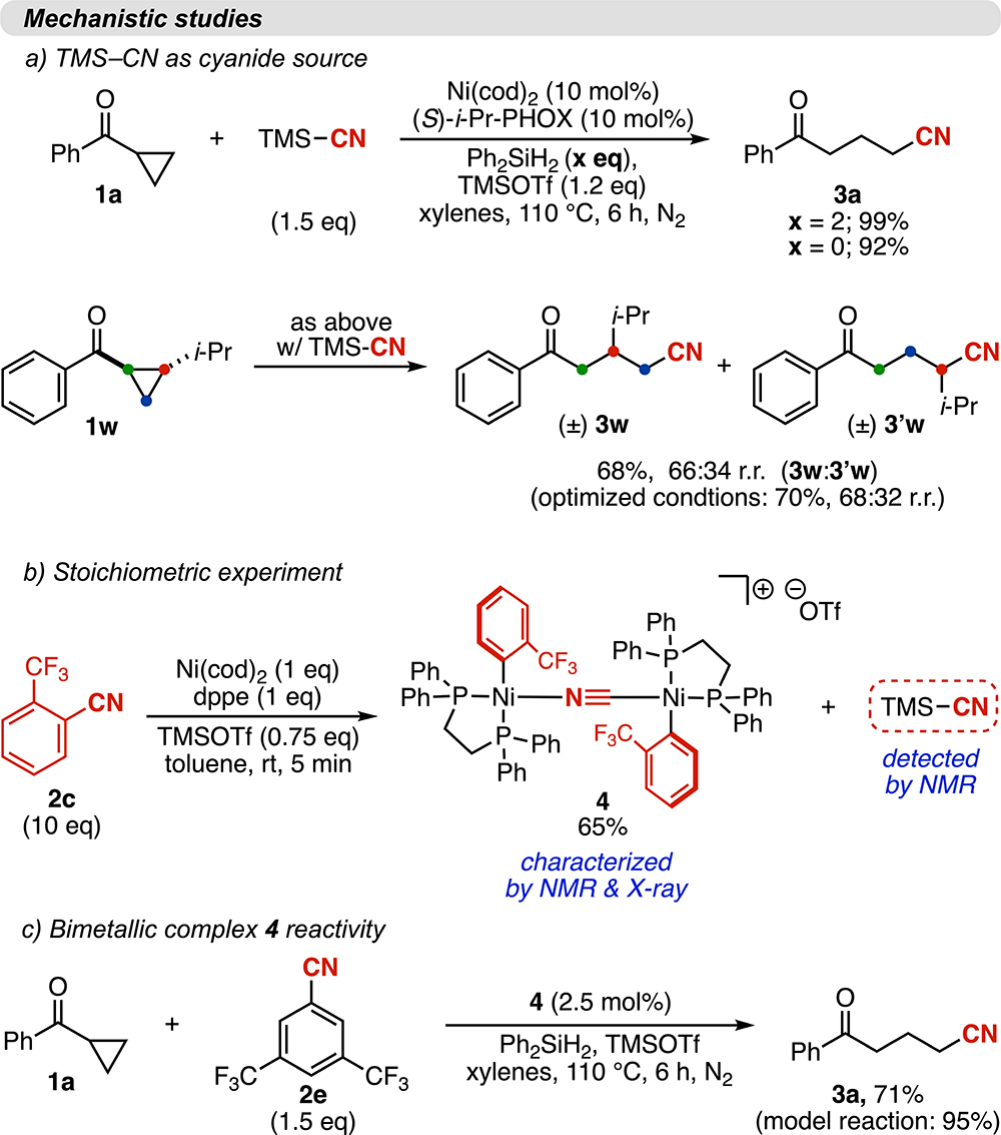
Mechanistic studies. (a) Reactions carried out with TMSCN as CN source. Reaction conditions: Ni­(cod)_2_ (0.0075 mmol), *(S)-i*-Pr-PHOX (0.0075 mmol), **1** (0.15 mmol), TMSCN (0.225 mmol), diphenyl silane (0.3 mmol, unless otherwise stated), TMSOTf (0.18 mmol), and xylenes (2.25 mL). Yields were determined by ^1^H NMR with dimethyl fumarate as an internal standard. (b) Stoichiometric experiment performed to study the C–CN activation mechanism. Reaction conditions: Ni­(cod)_2_ (0.8 mmol), dppe (0.8 mmol), **2c** (8 mmol), TMSOTf (0.6 mmol), and toluene (18 mL). (c) Reaction carried out with **4** as Ni source. Reaction conditions: **4** (0.00375 mmol), **1a** (0.15 mmol), TMSCN (0.225 mmol), diphenyl silane (0.3 mmol), TMSOTf (0.18 mmol), and xylenes (2.25 mL). The yield was determined by ^1^H NMR with dimethyl fumarate as an internal standard.

Intrigued by these results, we sought to determine whether these conditions enable access to an alternative reaction pathway or whether the mechanism that operates under the optimized benzonitrile conditions proceeds via initial TMSCN liberation. To challenge this hypothesis, we examined the reactivity of substrate **1w** using TMSCN. This experiment allows direct comparison of both product formation and regioselectivity. As shown in Figure 3a, comparable results to those obtained with benzonitrile **2e** as CN source were obtained. These findings suggest that the product-determining step is shared between the reaction under study and the TMSCN variant, again suggesting that TMSCN may be a reaction intermediate.

Finally, a time-course experiment comparing the model reaction with an otherwise identical reaction using TMSCN as the cyanating agent revealed a significantly faster reaction profile when TMSCN was employed. Full conversion to product was achieved within 15 min using TMSCN, whereas the model reaction reached only 40% yield over the same time period (see Figure S15). These observations provide further support for the proposal that TMSCN serves as an intermediate in the catalytic process.

To investigate whether TMSCN analogues are formed under our conditions, we conducted stoichiometric nickel studies aiming to generate the Ni–aryl cyanide complex in the presence of a Lewis acid, and subsequently observe the release of silyl isocyanide. For ease of analyzing the intermediates, we selected the symmetric bisphosphine ligand dppe, which affords slightly reduced yields under our conditions (see Figure S1). To our surprise, when the (dppe)­Ni(0) complex was stirred with the Lewis acid and benzonitrile **2c**, we did not observe formation of the expected Ni–aryl cyanide complex. Instead, a bimetallic complex featuring a bridging cyano ligand (**4**) was isolated and characterized by X-ray single crystal diffraction (Figure 3b). Additionally, bimetallic complex **4** was shown to be catalytically competent in the model reaction, affording **3a** in 71% yield.

Complex **4** is a rare example of a bimetallic four-coordinate nickel framework featuring a bridging cyano group.^66^ Both Ni centers adopt square-planar geometries characteristic of low-spin Ni­(II). The cyanide ligand displays a linear arrangement between the Ni atoms, with a C–N bond length of 1.147(8) Å, consistent with retention of the triple-bond character.^20^

Analysis of the mass balance of the reaction suggests the formation of an equivalent of TMSCN. To confirm that TMSCN is generated during the synthesis of **4**, the reaction was performed in an NMR tube and monitored by ^1^H NMR. A new signal at 0.01 ppm appeared concurrently with the formation of complex **4**. The identity of this signal was unambiguously established by spiking the reaction mixture with an authentic sample of TMSCN (see Figure S18). The formation of TMSCN was also observed under the optimal conditions in the absence of cyclopropyl ketone, even at room temperature, confirming that TMSCN generation is not specific to the dppe ligand system (see Figure S17). Efforts to identify the same diagnostic signal in the coupling reaction crude were inconclusive given the large number of overlapping signals arising from multiple organosilicon species present in the reaction.

We rationalized the formation of complex **4** and TMSCN as arising from initial Lewis acid-mediated oxidative addition to generate a [Ni­(aryl)­(CN → TMS)]­(OTf) species,^55^ which can dimerize to give **4** and release TMSCN. However, when equimolar or excess TMSOTf is added during the synthesis, akin to the catalytic conditions, (dppe)_2_Ni­(OTf)_2_ is obtained as the major product. This suggests that generating an additional equivalent of TMSCN is favored over the formation of complex **4** when [Ni] ≪ [TMSOTf].

Building upon these observations, we propose the mechanism shown in Figure 4, which features the formation of TMSCN as a key intermediate. While the possibility of a transmetalation between a putative Ni–CN species and the Ni enolate complex **IV** cannot be fully excluded, we believe that the data presented provides strong support for the proposed pathway; specifically:

(1) For the C–CN activation cycle: (i) the formation of TMSCN under the reaction conditions was demonstrated under the reaction conditions in the absence of cyclopropyl ketone and in the stoichiometric experiment shown in Figures 3b, S17 and S18, wherein oxidative addition of a Ni(0) complex into benzonitrile **2c** in the presence of TMSOTf was shown to generate one equivalent of TMSCN along with a Ni­(aryl)­(OTf) complex (**III**, Figure 4). (ii) The requirement of a silane for catalytic turnover, together with the detection of the reduced arene 5 in 92% yield under the optimized catalytic conditions, suggests that transmetalation of **II** with the silane forms Ni–hydride **III**, which subsequently undergoes reductive elimination to regenerate Ni(0).
(2) For the C–C coupling cycle: the opening of cyclopropyl ketones **1** in the presence of Ni(0) complexes and Lewis acids is precedented.^42,67^ Accordingly, we propose that a second Ni(0) complex **I** engages the ketone, generating the ring-opened intermediate **IV**.
(3) Regarding the intermediacy of TMSCN in the reaction: (i) the use of TMSCN as the cyanide source under otherwise optimized conditions led to product formation with yields and regioselectivities comparable to those obtained using benzonitriles (Figure 3a). (ii) The reaction employing TMSCN was shown to proceed with a significantly faster catalytic profile than the model reaction, consistent with its role as a reaction intermediate. On this basis, we propose TMSCN engages **IV** and delivers **3”** while regenerating Ni(0), thereby turning over the catalytic cycle. (iii) As previously mentioned, the formation of TMSCN under the reaction conditions at room temperature and in the absence of the cyclopropyl ketone was observed by ^1^H NMR (Figure S17)
(4) A full rate-law analysis revealed fractional reaction orders with respect to Ni, cyclopropyl ketone **1**, and the silane. In contrast, benzonitrile **2** exhibits a negative half-order (−0.5), while the Lewis acid displays saturation kinetics, approaching zero-order behavior under the reaction conditions (see Section S5). These results indicate that the turnover-limiting step most likely resides within the cyclopropyl ketone opening/C–C coupling cycle (depicted at the bottom of Figure 4). Indeed, although our reaction requires high temperature, benzonitrile activation occurs readily at room temperature (Figure 3b). On the other hand, cyclopropyl ketone activation typically requires elevated temperatures.^43–46^ Finally, we rationalize the negative order in benzonitrile as arising from desynchronization of the two catalytic cycles at higher benzonitrile concentrations and/or reversible, strong binding of benzonitrile to Ni, which sequesters the catalyst.

**Figure 4:**
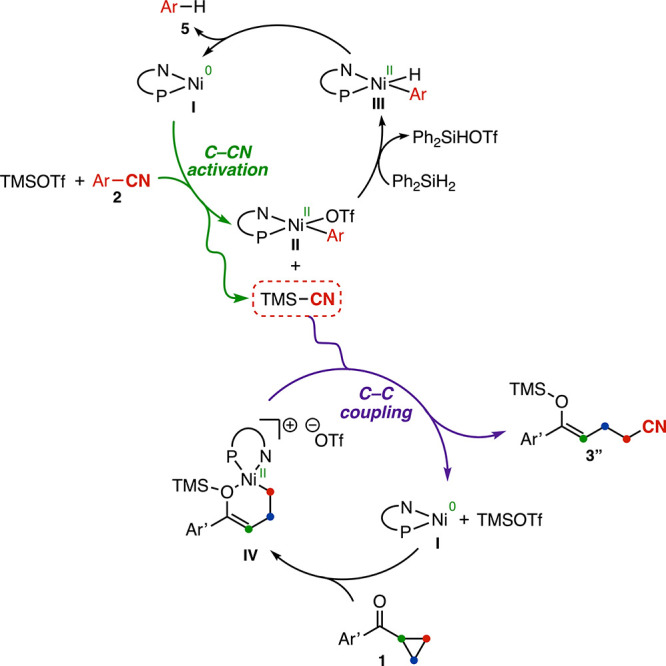
Mechanistic studies and proposed mechanism. The first catalytic turnover is shown for simplicity. Scrambling between the Lewis acid and silane is observed, with a variety of silicon species detected as the reaction proceeds (see Section S7).

## Conclusions

In summary, we have developed a Ni-catalyzed methodology that enables in situ generation of silyl cyanides directly from nontoxic benzonitriles, providing a safer and more versatile alternative to TMSCN. This approach decouples cyanide formation from the C–C bond-forming step, thereby expanding the scope of accessible cyanation transformations beyond transfer from one arene to another and hydrocyanation of a π-system. Cyclopropyl and cyclobutyl ketones were chosen as substrates to showcase the ability of this strategy to promote challenging couplings and afford ring-opening cyanation to form an array of γ- and δ-cyanated ketones.

Mechanistic studies, including kinetic analysis, stoichiometric reactions, NMR monitoring, and isolation of a rare bimetallic bridging-cyano Ni species, support a pathway in which oxidative addition, transmetalation, and silane-mediated catalyst turnover are key steps in two discrete catalytic cycles. This strategy overcomes limitations of prior cyanation methods that rely on metal–cyanide intermediates, providing a general platform for the safe and selective incorporation of cyanide into complex molecules. More broadly, we foresee this method for controlled in situ generation of silyl cyanides from nontoxic precursors as a platform ripe for integration with other established organometallic reactivity manifolds.

## Supplementary Material



## Notes

Experimental procedures and characterization and spectral data for new compounds (PDF)
