# Supplementary material for: Decoupling Cyanide Activation from C–C Bond Formation in Ni-Catalyzed Cyanation of Strained Ketones Using Benzonitriles
Source: J Am Chem Soc. 2026 Apr 2;148(14):14963–70. doi: 10.1021/jacs.5c21546 (PMC13088241; doi:10.1021/jacs.5c21546)
Supplement: Supplementary file 1 [file ja5c21546_si_001.pdf]

Decoupling Cyanide Activation from C–C Bond Formation in Ni-Catalyzed Cyanation of Strained Ketones Using Benzonitriles

Nathan J. Coddington, Robert D. Bradley,[‡] Yvette A. Luna,[‡] Madison D. Loper, Paul J. Saucedo, Rihan Ouyang, William L. Lo, Veronica Carta, and Ana Bahamonde*

[‡] Contributed equally to this manuscript

Department of Chemistry, University of California, Riverside

501 W Big Springs Rd., Riverside, CA, 92521, USA

E-mail: ana.bahamonde@ucr.edu

Table of Contents:

1. General considerations and methods S2
2. General procedures and experimental set up S3
   2.1. Ring opening and cyanation of strained ketones S3
   2.2. Synthesis of starting materials S4
3. Optimization S7
4. Starting material characterization S10
5. Kinetic experiments S20
6. Reactions using TMSCN S33
7. NMR experiments of stability of reaction components S36
8. Bimetallic bridging cyanide complex **4**: synthesis and reactivity S39
9. X-ray crystallographic data S46
10. Product characterization S49
11. References S63
12. ^{1}H, ^{13}C, ^{19}F, and ^{31}P NMR spectra S65

1. General considerations and methods

All reagents were purchased from commercial suppliers and distilled prior to use unless otherwise stated. ^{1}H NMR spectra were obtained in $CDCl_3$, benzene-d_6, or acetonitrile-d_3 at 400 MHz or 600 MHz. Chemical shifts are reported in ppm and referenced to the $CHCl_3$ singlet at 7.26 ppm, the benzene singlet at 7.16 ppm, or the acetonitrile singlet at 1.94 ppm. ^{13}C NMR spectra were obtained in $CDCl_3$, benzene-d_6, or acetonitrile-d_3 at 101 MHz or 151 MHz and referenced to the center peak of the $CDCl_3$ triplet at 77.16 ppm, the benzene triplet at 128.06 ppm, or the acetonitrile heptet at 1.32 ppm. ^{19}F NMR spectra were obtained in $CDCl_3$ or acetonitrile-d_3 at 376 MHz or 564 MHz and referenced to the trifluorotoluene singlet at -63.72 ppm of an external standard. 31P NMR spectra were obtained in acetonitrile-d_3 at 162 MHz or 243 MHz and referenced to the triphenylphosphine singlet at -6.00 of an external standard. The abbreviations s, d, t, quint, sext, hept, dd, ddd, dt, m, brs, brd, brt, brm, and ABq stand for the resonance multiplicities singlet, doublet, triplet, quintet, sextet, heptet, doublet of doublets, doublet of doublet of doublets, doublet of triplets, multiplet, broad singlet, broad doublet, broad triplet, broad multiplet, and AB quartet respectively. Thin-layer chromatography was performed with EMD silica gel 60 F254 plates eluting with solvents indicated, visualized by a 254 nm UV lamp, and stained with potassium permanganate ($KMnO_4$) or *p*-anisaldehyde. Flash chromatography was performed using EM reagent silica 60 (230-400 mesh). Melting points were obtained on a Barnstead Electrothermal 1101D Mel-Temp. High resolution accurate mass spectral data was obtained on an Agilent 6545 LC/SFC Hybrid Q-TOF instrument. NMR and mass spectra were all obtained using the Analytical Chemistry Instrumentation Facility at the University of California, Riverside. Compounds **1a**, **1i**, **1s**, were purchased from commercial suppliers and distilled prior to use. **1e** was purchased, treated with toluene, then concentrated under vacuum to azeotrope off trace water.

2. General procedures and experimental set up

2.1. Ring opening and cyanation of strained ketones

General procedure A

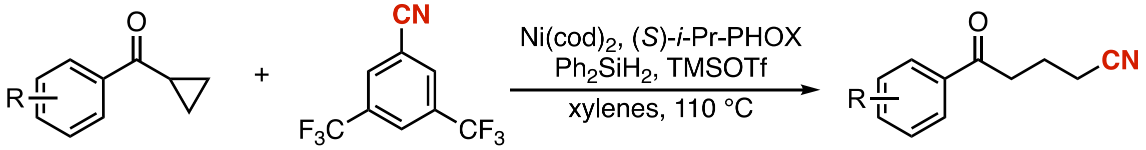

In a N_2 filled glove box, a stock solution of catalyst was prepared by charging a 1-dram vial with (*S*)-*i*-Pr-PHOX (0.03 mmol, 11.2 mg) and dissolving with 2 mL xylenes (0.015 M). Subsequently, $Ni(cod)_2$ (0.03 mmol, 8.3 mg) was weighed into a separate 1-dram vial to which the ligand solution was added by pipet. This stock solution is added to the reaction vessel at the appropriate time after all the nickel dissolved.

In a separate 2-dram vial equipped with a PTFE-coated lid, cyclopropyl ketone **1** (0.150 mmol) was added and dissolved with 1.25 mL xylenes. Next, 3,5-bis(trifluoromethyl)benzonitrile **2e** (0.225 mmol, 37.1 μL), diphenyl silane (0.300 mmol, 55.7 μL), and TMSOTf (0.180 mmol, 32.7 μL) were added. The mixture was gently swirled after each addition. At this stage, 1.0 mL (10 mol% Ni) of the previously prepared catalyst stock solution was added. The reaction vial was then capped, electrical taped, and placed in an aluminum block heated to 110 °C for 6 h. The reaction proceeded without stirring.

Upon completion, the reaction was removed from the glovebox and quenched by the addition of 1 mL of DCM and 2-4 drops of water, followed by vigorous mixing until the solution turned yellow. The solution was then dried with $MgSO_4$, filtered through cotton, and concentrated in vacuo. The resulting residue was purified by flash chromatography.

NOTE: The reaction maintains a dark wine-red that is almost black. Should the solution turn yellow prior to workup, the yield is expected to be drastically lower. This potentially indicates the presence of trace moisture, trace Lewis base, or an incompatible substrate.

2.2. Synthesis of starting materials

General procedure B

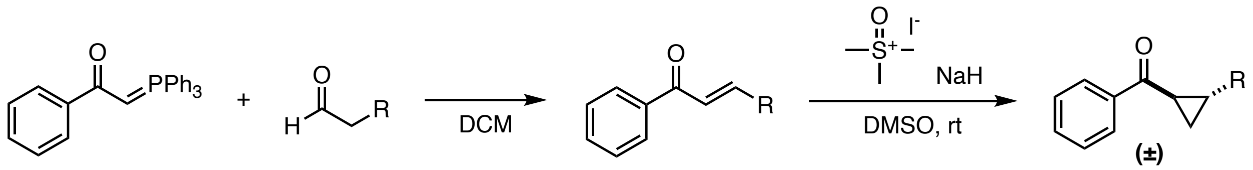

The following procedure was adapted from the literature.[1] To an oven-dried, nitrogen flushed, round bottom flask equipped with a PTFE-coated stir bar was added phosphonium ylide (10.0 mmol) followed by dry 25 mL DCM. Aldehyde (20.0 mmol) was then added dropwise and left to react for 48 h at room temperature under nitrogen. Once finished, the solvent was evaporated under vacuum and the resulting solid residue was filtered through a celite plug and washed with Et_2O 3x. The filtrate was then concentrated under vacuum and the resulting residue was purified by flash chromatography to afford the enone intermediate.

To an oven-dried, nitrogen flushed, 100 mL round bottom flask equipped with a PTFE-coated stir bar was added NaH (60% dispersion in mineral oil, 6.0 mmol), Me_3SOI (6.0 mmol) and 30 mL DMSO and allowed to stir at room temperature for 30 min. The enone (5.0 mmol) was dissolved in 2 mL DMSO and added dropwise then left to stir at room temperature for 24 h or at least overnight under nitrogen. Once completed, reaction was quenched with water and extracted with Et_2O 3x. The combined organic layers were washed with brine, dried with $MgSO_4$, filtered, and concentrated under gentle vacuum. The resulting oil was purified by flash chromatography.

General procedure C

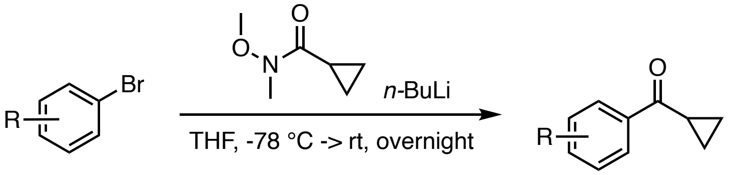

The following procedure was adapted from the literature.[2] To an oven-dried, 25 mL round bottom flask equipped with a magnetic stir bar was added aryl bromide (2.5 mmol) and 5 mL dry THF using air-free techniques. The reaction was cooled to -78 °C using a dry ice/acetone bath, and a *n*-BuLi solution in hexane (2.5 M, 2.265 mmol) was added and allowed to stir for 30 min. Cyclopropane Weinreb amide (2.265 mmol) was then added dropwise, and the solution was allowed to warm to room temperature and stirred overnight under nitrogen. Once completed, the reaction was poured into saturated NH_4Cl solution, extracted with Et_2O 3x, and the combined organic layers were washed with brine, dried with $MgSO_4$, filtered, and concentrated under vacuum. The resulting residue was purified by flash chromatography.

General procedure D

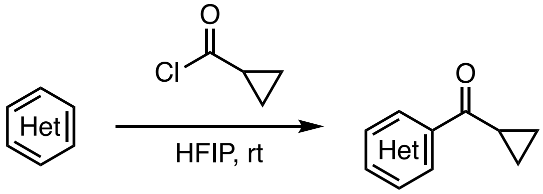

The following procedure was adapted from the literature.[3] To an oven-dried, nitrogen flushed, 25 mL round bottom flask equipped with a magnetic stir bar, heteroarene (1.2 mmol) was added, and dissolved in 5 mL of HFIP (0.1 M). The flask was placed in a room temperature water bath to control any exotherm, and cyclopropane carboxylic acid chloride (1.0 mmol) was added dropwise. The reaction was allowed to stir for 12 h at room temperature under nitrogen. The reaction was then poured into saturated $NaHCO_3$ solution and extracted with EtOAc 3x. Emulsions were cleared by the addition of brine and waiting. The combined organic layers were washed with brine, dried with $MgSO_4$, filtered, and concentrated under vacuum. The resulting residue was purified by flash chromatography.

General procedure E

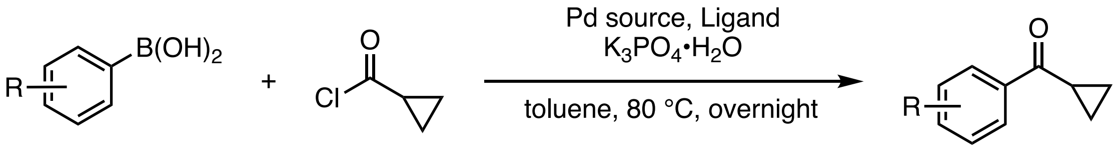

The following procedure was adapted from the literature.[4] To a two-neck 25 mL round bottom flask equipped with a magnetic stir bar was added boronic acid (1.5 mmol), tribasic potassium phosphate hydrate (2.25 mmol), triphenylphosphine (12 mol%), and palladium diacetate (3 mol%), followed by 8 mL of toluene. To the stirred suspension was added cyclopropane carboxylic acid chloride (1.8 mmol). The reaction was then heated to 80 °C in an oil bath for 18 h under nitrogen. On completion, the reaction was then filtered through a celite plug, washed with DCM, and concentrated under vacuum. The resulting residue was purified by flash chromatography.

3. Optimization

Figure S1: Optimization of ligand

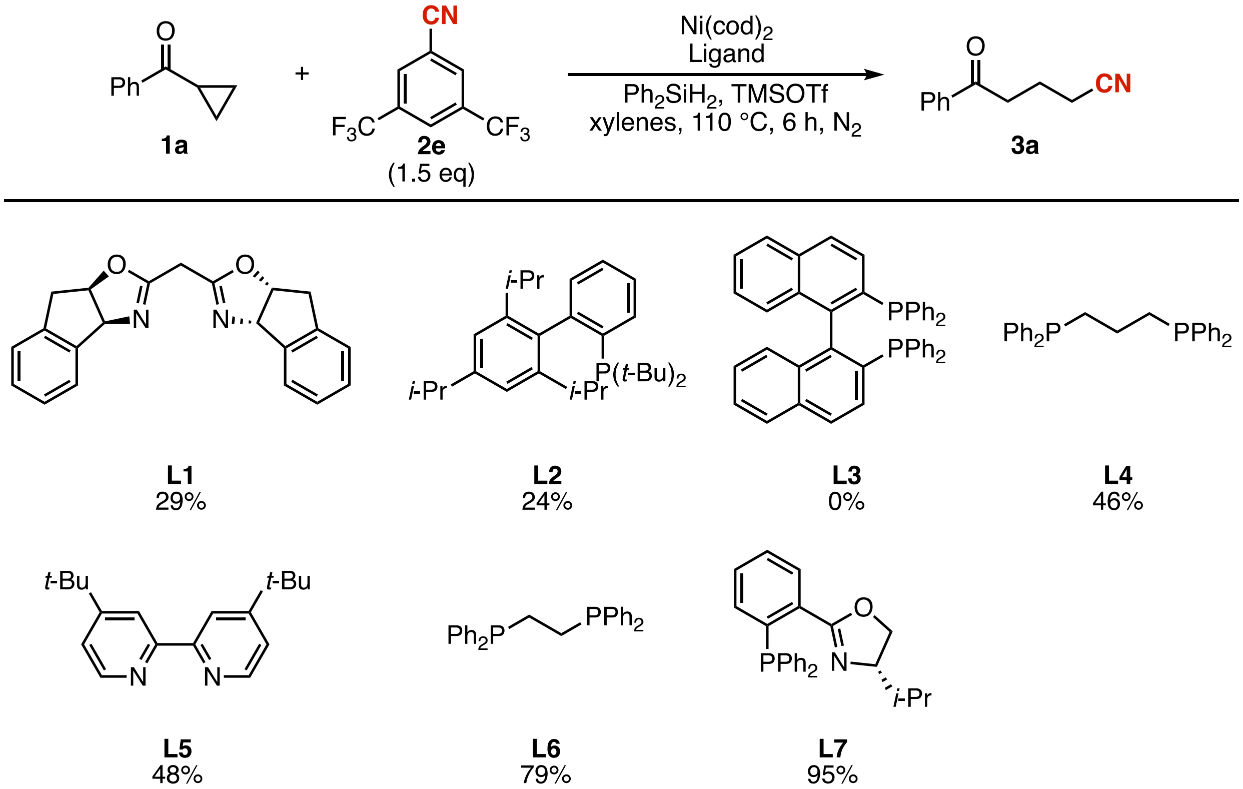

Reaction carried out following general procedure A, Ni(cod)$_2$ (0.005 mmol), **ligand** (0.005 mmol), **1a** (0.1 mmol), **2e** (0.15 mmol), diphenyl silane (0.2 mmol), TMSOTf (0.12 mmol), and xylenes (2 mL). The reaction was heated to 110 °C for 6 h. All yields were determined by ^{1}H NMR using dimethyl fumarate as an NMR standard.

Table S1: Optimization of Lewis acid

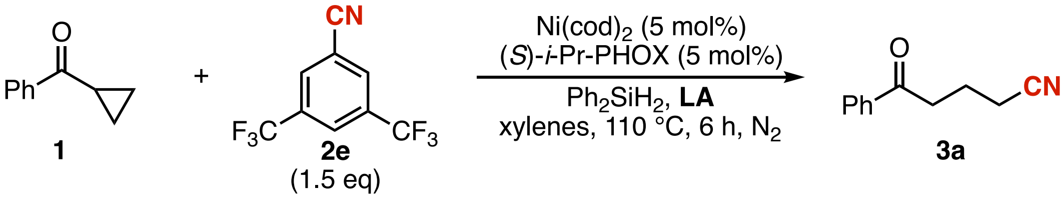

Entry	LA	Yield of 3a
1	**TMSOTf**	95%
2	**TESOTf**	99%
3	**TBSOTf**	0%
4	**TMSCl**	0%
5	**$Al(OTf)_3$**	0%

Reaction carried out following general procedure A, $Ni(cod)_2$ (0.0075 mmol), ligand (0.0075 mmol), **1a** (0.15 mmol), **2e** (0.225 mmol), diphenyl silane (0.3 mmol), **Lewis acid** (0.18 mmol), and xylenes (2.25 mL). The reaction was heated to 110 °C for 6 h. All yields were determined by ^{1}H NMR using dimethyl fumarate as an NMR standard.

Table S2: Optimization of reductant

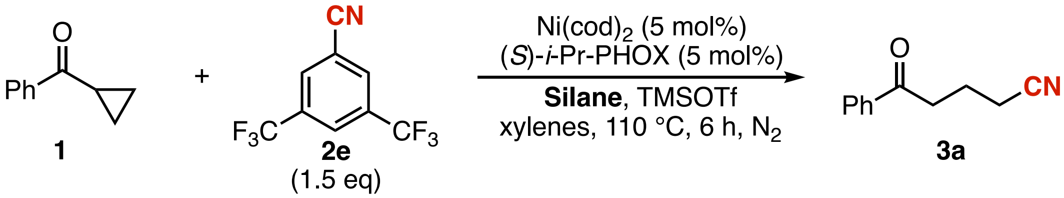

Entry	Silane	Yield of 3a
1	**Ph_2SiH_2**	95%
2	**Et_2SiH_2**	93%
3	**$(TMS)_3SiH$**	20%
4	**$(MeO)_3SiH$**	0%
5	**$(TMS)_2$**	0%
6	**Zn**[a]	10%
7	**Mn**[a]	22%

Reaction carried out following general procedure A, $Ni(cod)_2$ (0.0075 mmol), ligand (0.0075 mmol), **1a** (0.15 mmol), **2e** (0.225 mmol), **silane** (0.3 mmol), TMSOTf (0.18 mmol), and xylenes

(2.25 mL). The reaction was heated to 110 °C for 6 h. All yields were determined by ^{1}H NMR using dimethyl fumarate as an NMR standard. [a] Reaction was performed with $Ni(cod)_2$ (0.015 mmol), ligand (0.015 mmol).

Figure S2: Unsuccessful substrates

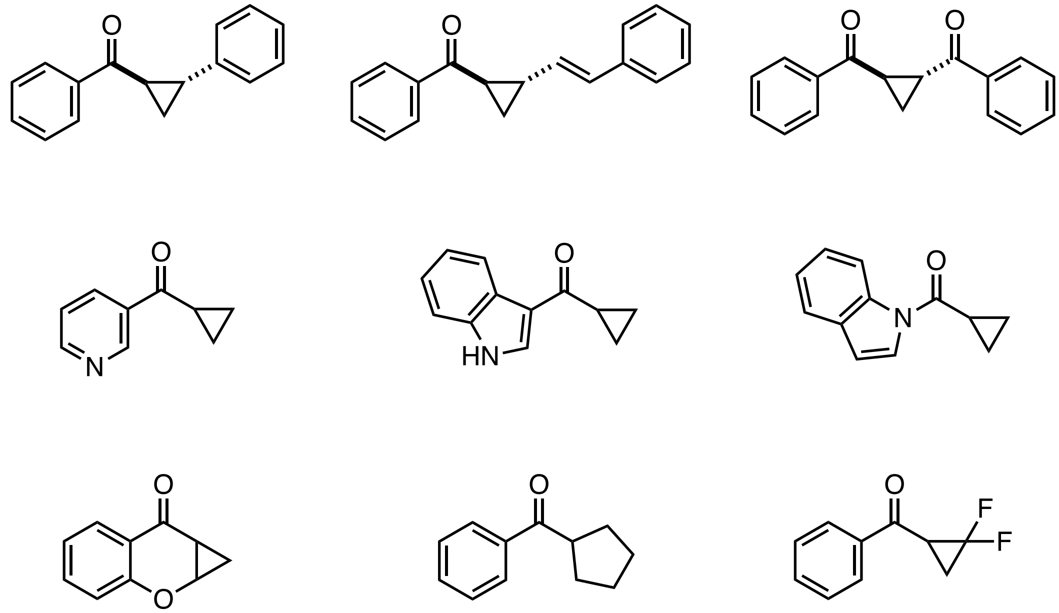

Reaction carried out following general procedure A, $Ni(cod)_2$ (0.015 mmol), ligand (0.015 mmol), **Substrate** (0.15 mmol), **2e** (0.225 mmol), diphenyl silane (0.3 mmol), TMSOTf (0.18 mmol), and xylenes (2.25 mL). The reaction was heated to 110 °C for 6 h. Trace product formation was detected by ^{1}H NMR.

4. Starting material characterization

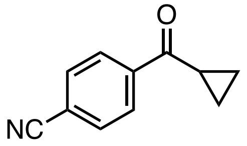

4-cyanophenyl cyclopropyl ketone (**1b**)

Following general procedure C, 4-bromobenzonitrile (1.0 mmol, 182.0 mg) was employed. The resulting residue was purified by flash chromatography (silica, 10% EtOAc in hexanes, ***Rf*** = 0.176 in 10% EtOAc in hexanes) to afford 99.0 mg (58% yield) of **1b** as a white solid. Characterization data matches previous reports.[5]

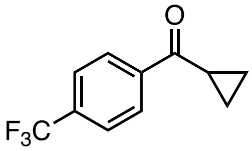

4-(trifluoromethyl)phenyl cyclopropyl ketone (**1c**)

Following general procedure C, 4-bromobenzotrifluoride (2.5 mmol, 1350 μL) was employed. The resulting residue was purified by flash chromatography (silica, 5% Et_2O in hexanes, ***Rf*** = 0.3 in 5% Et_2O in hexanes) and then distilled under mild vacuum to afford 225.3 mg (42% yield) of **1c** as a colorless oil. Characterization data matches previous reports.[2]

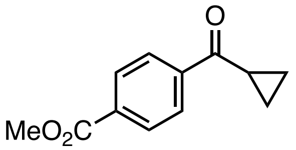

methyl 4-(cyclopropanecarbonyl)benzoate (**1d**)

Following general procedure E, methyl 4-bromobenzoate (1.5 mmol, 270.0 mg) was employed. The resulting residue was purified by flash chromatography (silica, 10% EtOAc in hexanes, ***Rf*** = 0.22 in 10% EtOAc in hexanes) to afford 110.0 mg (36% yield) of **1d** as an off-white solid. Characterization data matches previous reports.[6]

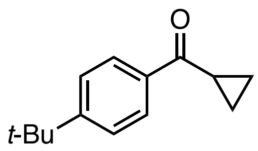

4-*tert*-butylphenyl cyclopropyl ketone (**1f**)

Following general procedure C, 1-bromo-4-*t*-butylbenzene (1.05 mmol, 182 μL) was employed. The resulting residue was purified by flash chromatography (silica, 5% Et_2O in hexanes, **R*f*** = 0.26 in 5% Et_2O in hexanes) to afford 93.2 mg (46% yield) of **1f** as a white solid. Characterization data matches previous reports.[7]

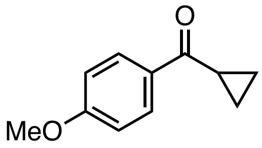

4-methoxyphenyl cyclopropyl ketone (**1g**)

Following general procedure C, 4-bromoanisole (1.0 mmol, 126 μL) was employed. The resulting residue was purified by flash chromatography (silica, 10% Et_2O in hexanes, **R*f*** = 0.1762 in 10% Et_2O in hexanes) to afford 119.0 mg (68% yield) of **1g** as a white solid. Characterization data matches previous reports.[7]

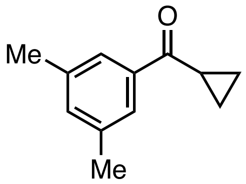

cyclopropyl(3,5-dimethylphenyl)methanone (**1h**)

Following general procedure C, 5-bromo-*m*-xylene (1.0 mmol, 185.1 mg) was employed. The resulting residue was purified by flash chromatography (silica, 2.5% Et_2O in hexanes, **R*f*** = 0.228 in 2.5% Et_2O in hexanes) to afford 122.8 mg (70% yield) of **1h** as a white solid. Characterization data matches previous reports.[7]

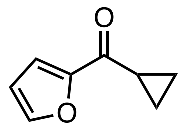

cyclopropyl(furan-2-yl)methanone (**1j**)

Following general procedure D, furan (2.0 mmol, 136.2 mg) was employed. The resulting residue was purified by flash chromatography (silica, 0-15% gradient EtOAc in hexanes, **R*f*** = 0.25 in 15% EtOAc in hexanes) to afford 62.9 mg (23% yield) of **1j** as a yellow oil. **^{1}H NMR** (600 MHz, $CDCl_3$) δ 7.61 (d, *J* = 1.7 Hz, 1H), 7.22 (d, *J* = 3.5 Hz, 1H), 6.55 (dd, *J* = 3.5, 1.7 Hz, 1H), 2.58 (tt, *J* = 7.9, 4.6 Hz, 1H), 1.23 (dt, *J* = 7.2, 3.4 Hz, 2H), 1.02 (dq, *J* = 7.2, 3.4 Hz, 2H). **^{13}C{^{1}H} NMR** (151 MHz, $CDCl_3$) δ 189.4, 153.3, 146.3, 116.5, 112.2, 17.2, 11.3. **HRMS** (ESI-TOF) m/z calculated for $C_8H_8O_2$ $(M+H)^+$: 137.0797 found 137.0602.

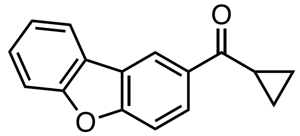

cyclopropyl(dibenzo[*b*,*d*]furan-2-yl)methanone (**1k**)

Following general procedure E, dibenzo[*b*,*d*]furan-2-ylboronic acid (1.0 mmol, 212.1 mg) was employed with $Pd(PPh_3)_2Cl_2$ (2 mol%, 14.0 mg) as the palladium source with no added ligand. The resulting residue was purified by flash chromatography (silica, 0-20% gradient EtOAc in hexanes, **R*f*** = 0.28 in 20% EtOAc in hexanes) to afford 60.6 mg (26% yield) of **1k** as a white solid (**mp** = 80.2-83.5 °C). **^{1}H NMR** (400 MHz, $CDCl_3$) δ 8.66 (dd, *J* = 1.9, 0.7 Hz, 1H), 8.19 (dd, *J* = 8.6, 1.9 Hz, 1H), 8.02 (ddd, *J* = 7.7, 1.4, 0.7 Hz, 1H), 7.66 – 7.58 (m, 2H), 7.51 (ddd, *J* = 8.4, 7.3, 1.4 Hz, 1H), 7.40 (td, *J* = 7.5, 1.0 Hz, 1H), 2.81 (tt, *J* = 7.8, 4.5 Hz, 1H), 1.34 – 1.28 (m, 2H), 1.14 – 1.07 (m, 2H). **^{13}C{^{1}H} NMR** (101 MHz, $CDCl_3$) δ 199.9, 158.9, 157.0, 133.5, 128.0, 127.9, 124.7, 124.0, 123.5, 121.5, 121.1, 112.1, 111.7, 17.4, 11.8. **HRMS** (ESI-TOF) m/z calculated for $C_{16}H_{12}O_2$ $(M+H)^+$: 237.0910 found 237.0909.

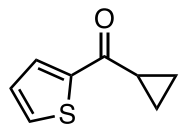

cyclopropyl(thiophen-2-yl)methanone (**1l**)

Following general procedure D, thiophene (2.0 mmol, 168.3 mg) was employed. The resulting residue was purified by flash chromatography (silica, 5% gradient EtOAc in hexanes, **R*f*** = 0.25 in 5% EtOAc in hexanes) to afford 65.6 mg (66% yield) of **1l** as a yellow solid. Characterization data matches previous reports.[8]

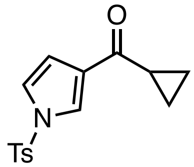

cyclopropyl(1-tosyl pyrrol-3-yl)methanone (**1m**)

Prepared according to a modified procedure D.[9] $AlCl_3$ (1.5 mmol, 200.0 mg) was added to an oven-dried two-neck 50 mL round bottom flask with a stir bar, followed by 20 mL DCM. The flask was cooled with a room temperature water bath and cyclopropane carboxylic acid chloride (1.36 mmol, 129 μL) was added dropwise, and the solution was stirred for 1 h or until clear. *N*-tosylpyrrole (1.36 mmol, 554.3 mg) was then added and stirred for 1 h. Off-gassing of HCl was observed. The reaction was cautiously quenched with methanol and washed with Rochelle's salt 2x and saturated Na_2CO_3 3x. The organic layer was dried with $MgSO_4$, filtered, and concentrated under vacuum. The resulting residue was purified by flash chromatography (silica, 10% EtOAc in hexanes, **R*f*** = 3.3 in 10% EtOAc in hexanes) to afford 193.0 mg (49% yield) of **1m** as a yellow oil. **^{1}H NMR** (400 MHz, C_6D_6) δ 7.90 (t, *J* = 2.1 Hz, 1H), 7.61 (d, *J* = 8.2 Hz, 2H), 7.00 (dd, *J* = 3.3, 2.1 Hz, 1H), 6.76 – 6.65 (m, 3H), 1.91 – 1.76 (m, 4H), 1.13 (p, *J* = 3.5 Hz, 2H), 0.54 (dq, *J* = 7.1, 3.5 Hz, 2H). **^{13}C{^{1}H} NMR** (101 MHz, C_6D_6) δ 194.2, 145.7, 135.8, 130.5, 130.3, 127.3, 124.4, 121.8, 112.8, 21.2, 18.1, 10.9. **HRMS** (ESI-TOF) m/z calculated for $C_{15}H_{15}NO_3S$ $(M+H)^+$: 290.0845 found 290.0840.

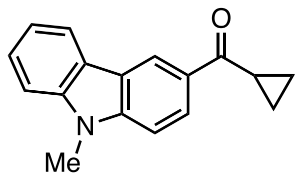

cyclopropyl(9-methyl-carbazol-3-yl)methanone (**1n**)

Following general procedure D, *N*-methyl carbazole (1.0 mmol, 181.2 mg) was employed. The resulting residue was purified by flash chromatography (silica, 0-15% gradient EtOAc in hexanes, **R*f*** = 0.25 in 15% EtOAc in hexanes) to afford 157.8 mg (63% yield) of **1n** as a yellow solid (**mp** = 106.8-108.8 °C). **^{1}H NMR** (400 MHz, $CDCl_3$) δ 8.83 (dd, *J* = 1.7, 0.7 Hz, 1H), 8.22 (dd, *J* = 8.6, 1.7 Hz, 1H), 8.16 (ddd, *J* = 7.8, 1.2, 0.7 Hz, 1H), 7.53 (ddd, *J* = 8.1, 7.1, 1.0 Hz, 1H), 7.47 – 7.41 (m, 2H), 7.31 (ddd, *J* = 8.1, 7.1, 1.0 Hz, 1H), 3.89 (s, 3H), 2.87 (tt, *J* = 7.8, 4.6 Hz, 1H), 1.33 – 1.28 (m, 2H), 1.11 – 1.04 (m, 2H). **^{13}C{^{1}H} NMR** (101 MHz, $CDCl_3$) δ 200.0, 143.7, 141.8, 129.7, 126.6, 126.4, 123.3, 122.7, 121.6, 120.7, 120.1, 109.1, 108.2, 29.5, 17.0, 11.4. **HRMS** (ESI-TOF) m/z calculated for $C_{17}H_{15}NO$ $(M+H)^+$: 250.1227 found 250.1230.

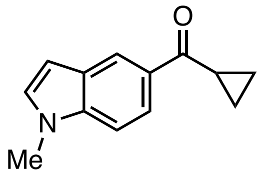

cyclopropyl(1-methyl-indol-5-yl)methanone (**1o**)

Following general procedure C, 5-bromo-*N*-methylindole (1.35 mmol, 284.2 mg) was employed. The resulting residue was purified by flash chromatography (silica, 0-10% gradient EtOAc in hexanes, **R*f*** = 0.14 in 10% EtOAc in hexanes) to afford 192.0 mg (71% yield) of **1o** as a yellow solid (**mp** = 137.8-142.4 °C). **^{1}H NMR** (400 MHz, $CDCl_3$) δ 8.41 (dd, *J* = 1.7, 0.8 Hz, 1H), 7.95 (dd, *J* = 8.8, 1.7 Hz, 1H), 7.35 (dt, *J* = 8.8, 0.8 Hz, 1H), 7.12 (d, *J* = 3.2 Hz, 1H), 6.62 (dd, *J* = 3.2, 0.9 Hz, 1H), 3.83 (d, *J* = 1.4 Hz, 3H), 2.80 (tt, *J* = 7.9, 4.6 Hz, 1H), 1.30 – 1.20 (m, 2H), 1.08 – 0.95 (m, 2H). **^{13}C{^{1}H} NMR** δ (101 MHz, $CDCl_3$) 200.6, 139.2, 130.4, 130.4, 128.1, 122.9, 121.9, 109.2, 103.1, 33.2, 17.0, 11.2. **HRMS** (ESI-TOF) m/z calculated for $C_{13}H_{13}NO$ $(M+H)^+$: 200.1070 found 200.1064.

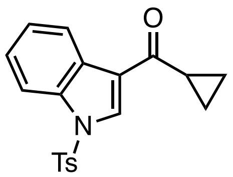

cyclopropyl(1-tosylindol-3-yl)methanone (**1p**)

Following general procedure D, indole (15 mmol, 1.757 g) was employed in 3x excess to the acid chloride. The resulting residue was purified by flash chromatography (silica, 20% EtOAc in hexanes, ***Rf*** = 0.5 in 30% EtOAc in hexanes) to afford the product as a pink and brown solid. The material was then recrystallized from hexanes/acetone to give 340.0 mg (37% yield) of the unprotected 3-acyl indole as white needles. Characterization data matches previous reports.[3]

The free acylated indole was then protected according to literature procedure.[10] 3-Acyl indole (0.6 mmol, 111.1 mg) was added to an oven-dried two-neck 25 mL round bottom flask equipped with a stir bar and dissolved in 10 ml dry THF using air-free techniques. The solution was cooled to 0 °C, and NaH (60% dispersion in mineral oil, 0.6 mmol, 24.0 mg) was added in portions. The reaction was allowed to warm to room temperature and was stirred for 1 h. Tosyl chloride (0.6 mmol, 114.4 mg) was added, and the reaction was allowed to stir for 24 h under nitrogen. Once complete, the solvent was evaporated under reduced pressure, and the resulting residue was washed with 20 mL water and extracted with EtOAc 3x. The combined organic layers were washed with brine, dried with $MgSO_4$, filtered, and concentrated under vacuum. The resulting solid was azeotropically dried with toluene to give 194.0 mg (95% yield) of **1p** as an analytically pure brown solid. Characterization data matches previous reports.[11]

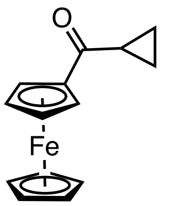

cyclopropyl ferrocenyl ketone (**1q**)

Prepared following the same modified procedure as **1m**. Ferrocene (1.0 mmol, 186.0 mg) was employed. The resulting residue was purified by flash chromatography (silica, 10% EtOAc in hexanes, ***Rf*** = 0.23 in 10% EtOAc in hexanes) to afford 153.5 mg (50% yield) of **1q** as a brown-orange solid. Characterization data matches previous reports.[12]

Synthesis of (1-methylcyclopropyl)(phenyl)methanone (1r)

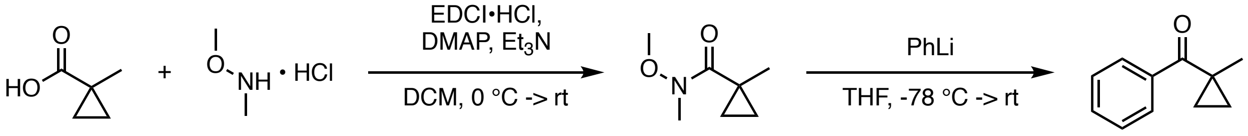

The Weinreb amide intermediate was synthesized according to literature procedure.[13] An oven-dried 50 mL round bottom flask equipped with a magnetic stir bar was charged with 1-methyl cyclopropane carboxylic acid (5.0 mmol, 476.0 mg), DMAP (1.0 mmol, 122.0 mg), *N*,*O*-dimethylhydroxylamine·HCl (7.5 mmol, 732.0 mg). The solids were suspended in 25 mL of dry DCM and the mixture was cooled to 0 °C. Freshly distilled triethylamine (16.5 mmol, 2.3 mL) was added dropwise, resulting in white precipitate. EDCI·HCl (6.0 mmol, 1.150 g) was added, and the reaction was allowed to warm to room temperature and stir overnight. Once completed, the reaction was poured into 1 M HCl, washed with 1 M HCl 2x, and washed with sat Na_2CO_3 2x. The organic phase was dried with $MgSO_4$, filtered, and concentrated under mild vacuum to afford 419.3 mg (59% yield) of analytically pure Weinreb amide as a colorless oil. Characterization data matches previous reports.[14] The amide was vacuum distilled under nitrogen prior to use.

1r was synthesized analogously to literature procedure.[15] Using air-free techniques, the previously synthesized Weinreb amide (1.5 mmol, 215.0 mg) was charged to an oven-dried two-neck 25 mL round bottom flask equipped with a magnetic stir bar. 15 mL of dry THF was added and the solution was cooled to -78 °C, then PhLi solution in Bu_2O (1.9 M, 1.575 mmol) was added dropwise by syringe. The reaction was then allowed to warm to room temperature and stir over night under nitrogen. To quench, the reaction was poured into 1 M HCl and extracted with Et_2O 3x. The combined organic layers were washed with brine, dried with $MgSO_4$, filtered, and concentrated under vacuum. The resulting residue was purified by flash chromatography (silica, 5% Et_2O in hexanes, ***Rf*** = 0.3 in 10% Et_2O in hexanes) to give 138.0 mg (57% yield) of **1r** as a colorless oil. Characterization data matches previous reports.[16]

Synthesis of (±)-*trans*-2-methylcyclopropyl(phenyl)methanone (1t)

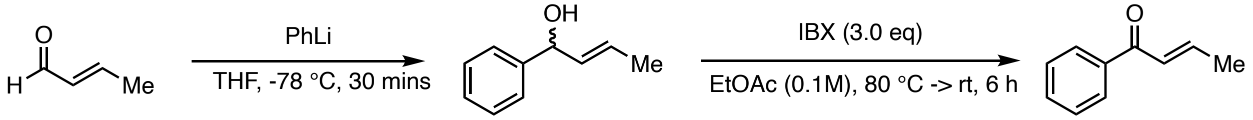

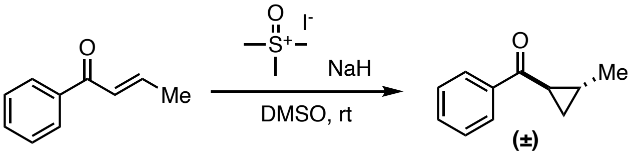

1t was synthesized adapting the following literature precedents.[17,18] IBX was synthesized according to known procedures.[19] To an oven-dried, nitrogen flushed, round bottom flask equipped with a PTFE-coated stir bar was added 20 mL dry THF then chilled to -78 °C (acetone/dry ice bath). Then a PhLi solution in Bu_2O (1.9 M, 5.25 mmol) was added, followed by slow dropwise addition of crotonaldehyde (5 mmol, 414.3 μL). The reaction was stirred for an additional 30 min at -78 °C once the addition was complete. The reaction was then quenched with 10 mL of saturated NH_4Cl. The volatile organics were removed under vacuum and the remaining solution was extracted with Et_2O 2x. The combined organics were dried with Na_2SO_4, filtered, and concentrated under vacuum to yield the alcohol, which was used without further purification.

The alcohol was transferred to a two-neck round bottom flask and dissolved with EtOAc (55 mL, 0.10 M), followed by the addition of IBX (15.0 mmol, 4.20 g). The reaction was left to reflux for 6 h under nitrogen. Once completed, the solution was concentrated under vacuum and the resulting

residue was purified by flash chromatography (silica, 4% TBME in hexanes, **Rf** = 0.19 in 4% TBME in hexanes) to afford 264.9 mg (36% yield, E/Z = 98:2) of the enone, as a yellow oil. Characterization data matches previous reports.[20]

Following the last step of general procedure B, the previously synthesized enone (1.78 mmol, 264.9 mg) was employed. The resulting residue was purified by flash chromatography (silica, 2.5% Et_2O in hexanes, **Rf** = 0.147 in 2.5% Et_2O in hexanes) to afford 51.0 mg (18% yield, d.r. = 97:3) of **1t** as a yellow oil. Characterization data matches previous reports.[1]

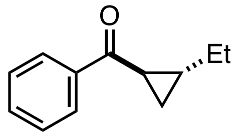

(±)-*trans*-2-ethylcyclopropyl(phenyl)methanone (**1u**)

Following general procedure B, propionaldehyde (10.0 mmol, 717 μL) was employed. The resulting residue was purified by flash chromatography (silica, 5% Et_2O in hexanes, **Rf** = 0.27 in 5% Et_2O in hexanes) to afford 1.135 g (70% yield, E/Z = 99:1) of the enone, as a yellow oil. Characterization data matches previous reports.[20]

Still following general procedure B, the previously synthesized enone (3.61 mmol, 579.0 mg) was employed. The resulting residue was purified by flash chromatography (silica, 0-4% TBME in hexanes, **Rf** = 0.29 in 4% TBME in hexanes) to afford 490.9 mg (78% yield, d.r. = 94:6) of **1u** as a pale-yellow oil. Characterization data matches previous reports.[1]

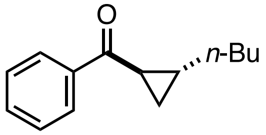

(±)-*trans*-2-butylcyclopropyl(phenyl)methanone (**1v**)

Following general procedure B, pentanal (1.5 mmol, 160 µL) was employed. The resulting residue was purified by flash chromatography (silica, hexanes, **R*f*** = 0.21 in hexanes) to afford 134.1 mg (70% yield, E/Z = 94:6) of the enone, as a yellow oil. Characterization data matches previous reports.[21]

Still following general procedure B, the previously synthesized enone (0.711 mmol, 134.1 mg) was employed. The resulting residue was purified by flash chromatography (silica, in hexanes, **R*f*** = 0.11 in hexanes) to afford 105.4 mg (73% yield, d.r. = 98:2) of **1v** as a yellow oil. Characterization data matches previous reports.[22]

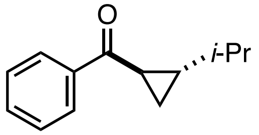

(±)-*trans*-2-isopropylcyclopropyl(phenyl)methanone (**1w**)

Following general procedure B, isobutyraldehyde (20 mmol, 7.609 g) was employed. The resulting residue was purified by flash chromatography (silica, 0-2% TBME in hexanes, **R*f*** = 0.19 in 2% TBME in hexanes) to afford 1.584 g (46% yield, E/Z = 99:1) of the enone, as a yellow oil.

Still following general procedure B, the previously synthesized enone (10.7 mmol, 1.584 g) was employed. The resulting residue was purified by flash chromatography (silica, 0-3% Et_2O in hexanes, **R*f*** = 0.21 in 3% Et_2O in hexanes) to afford 1.109 g (55% yield, d.r = 96:4) of **1w** as a yellow oil. **^{1}H NMR** (600 MHz, $CDCl_3$) δ 8.05 – 8.03 (m, 2H), 7.61 – 7.57 (m, 1H), 7.51 (t, *J* = 7.7 Hz, 2H), 2.52 (dt, *J* = 8.2, 4.3 Hz, 1H), 1.51 – 1.47 (m, 2H), 1.21 (p, *J* = 7.2 Hz, 1H), 1.06 (t, *J* = 7.2 Hz, 6H), 0.99 (dq, *J* = 7.9, 2.5 Hz, 1H). **^{13}C{^{1}H} NMR** (151 MHz, $CDCl_3$) δ 200.3, 138.2, 132.8, 128.6, 128.1, 35.4, 32.8, 24.5, 22.2, 21.9, 18.2.

5. Kinetic experiments

Kinetic experiments were conducted in 10 mL identical Schlenk tubes with four Schlenk tubes running simultaneously in a preheated silicone oil bath set to 110 ºC with an RPM setting of 440. The Schlenk tubes were positioned side by side on additional stands surrounding the hot plate. Among the kinetic setups, one Schlenk tube contained the prototypical control reaction, and the additional three contained a variation of a modified new reaction condition were tested. Each reaction vessel was rotated within the Schlenk line setup to validate the results of each condition. If the control reaction among each additional reaction condition displayed consistent reaction profiles, then the kinetic profiles of experiments testing the new conditions would be considered valid data. Stock solutions were used and handled in the glovebox to set up the reactions. Small aliquots (100 µL) were taken at indicated times by opening the reaction vessel under positive N_2 pressure to ensure an inert pressure atmosphere inside the Schlenk tubes were maintained. Each aliquot was immediately injected and mixed into a prefilled 1-dram vial with a PTFE-lined cap containing DCM (250 µL) and H_2O (2-3 drops). For every plot depicted, all reactions were carried out on the same day using the same stock solution. This procedure was later repeated on a different day to ensure reproducibility and accuracy of the results.

Model reaction and general procedures

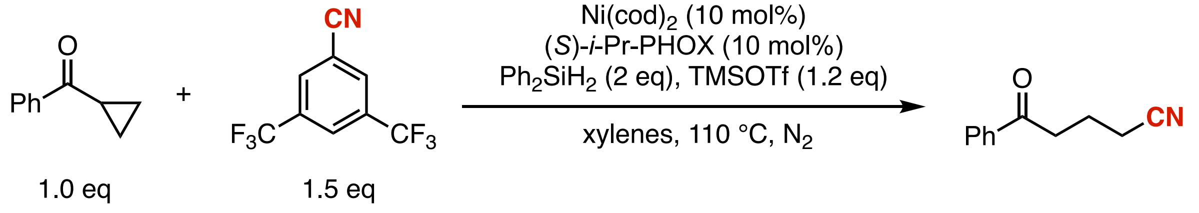

Stock solutions were used to setup the reactions.

Stock solution A: (*S*)-*i*-Pr-PHOX (0.082 mmol, 30.8 mg) was dissolved in 2.5 mL xylenes (0.033 M). Subsequently, $Ni(cod)_2$ (0.082 mmol, 22.7 mg) was weighed into a separate 1-dram vial. Once (*S*)-*i*-Pr-PHOX is fully dissolved, the ligand solution was pipetted into the vial containing the $Ni(cod)_2$.

Stock solution B: Cyclopropyl phenyl ketone (0.75 mmol, 104 µL) was added and dissolved with 5.0 mL xylenes in a 20 mL scintillation vial. Then 3,5-bis(trifluoromethyl)benzonitrile (1.125 mmol, 186 µL), and diphenyl silane (1.5 mmol, 279 µL) were added.

Oven-dried Schlenk tubes were brought into the glovebox with septum stoppers, and 1.1 mL stock solution B was added. Additional xylenes (0.84 mL) were added to ensure the total solvent volume was 2.25 mL (0.67 M) and followed by the addition of TMSOTf (33 μL) to the Schlenk tube. Lastly, stock solution A, 0.41 mL (0.015 mmol, 10 mol% Ni and ligand) was added. Once stock solution B, additional xylenes, TMSOTf, and stock solution A were mixed, they were immediately brought out of the glovebox, cycled onto the Schlenk line, and lowered into the oil bath within 5-7 min. Aliquots were taken at the indicated times and worked up before ^{1}H NMR analysis. The yields of the product were determined by cyclopropyl ketone conversion from ^{1}H NMR analysis. These conditions account for the following amounts: Ni(cod)$_2$ (0.015 mmol), (*S*)-*i*-Pr-PHOX (0.015 mmol), cyclopropyl phenyl ketone (0.15 mmol), 3,5-bis(trifluoromethyl)benzonitrile (0.225 mmol), diphenyl silane (0.30 mmol), and TMSOTf (0.18 mmol), and 2.25 mL xylenes.

Rate dependence with respect to Ni(cod)$_2$ and (*S*)-*i*-Pr-PHOX loading:

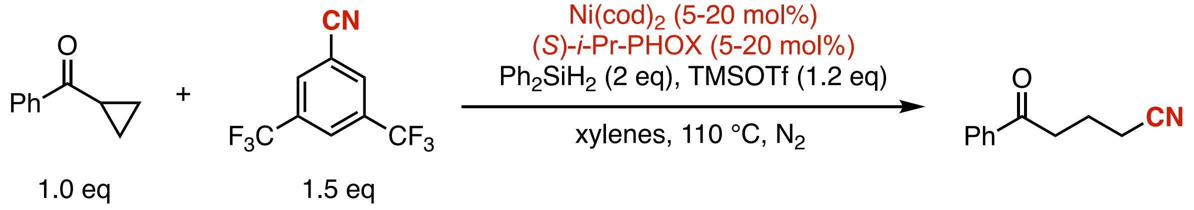

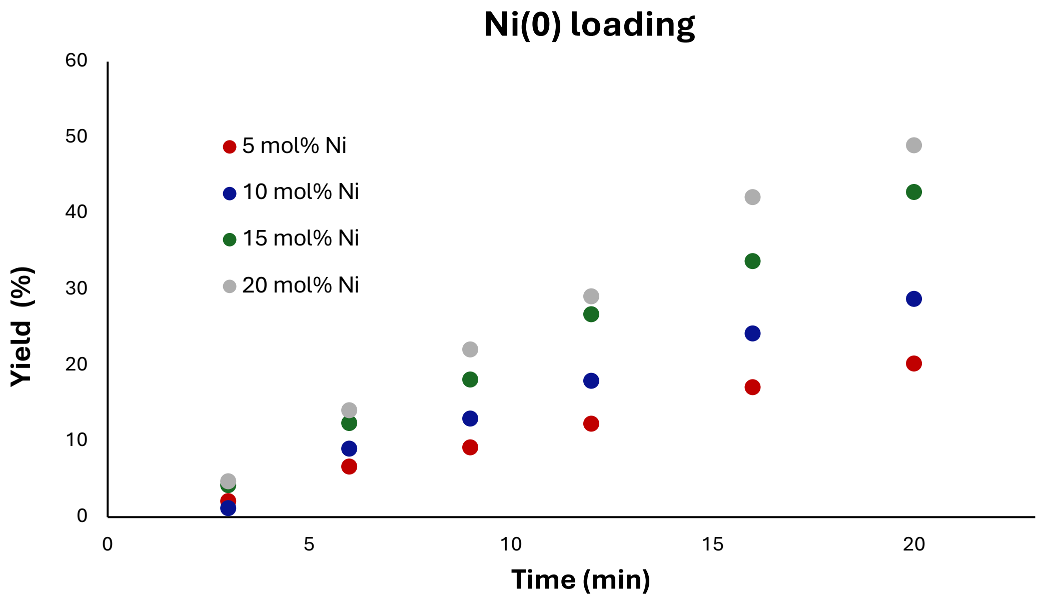

Figure S3: Reaction profiles for different Ni(cod)$_2$ concentrations.

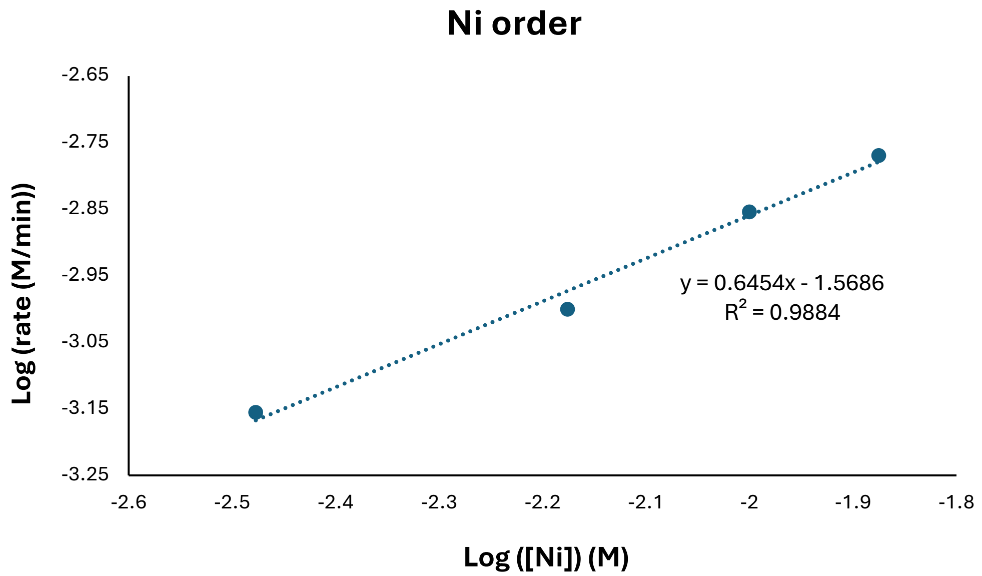

Figure S4: Logarithm of rate *vs* logarithm of $Ni(cod)_2$ concentration displaying 0.6 order with respect to $Ni(cod)_2$.

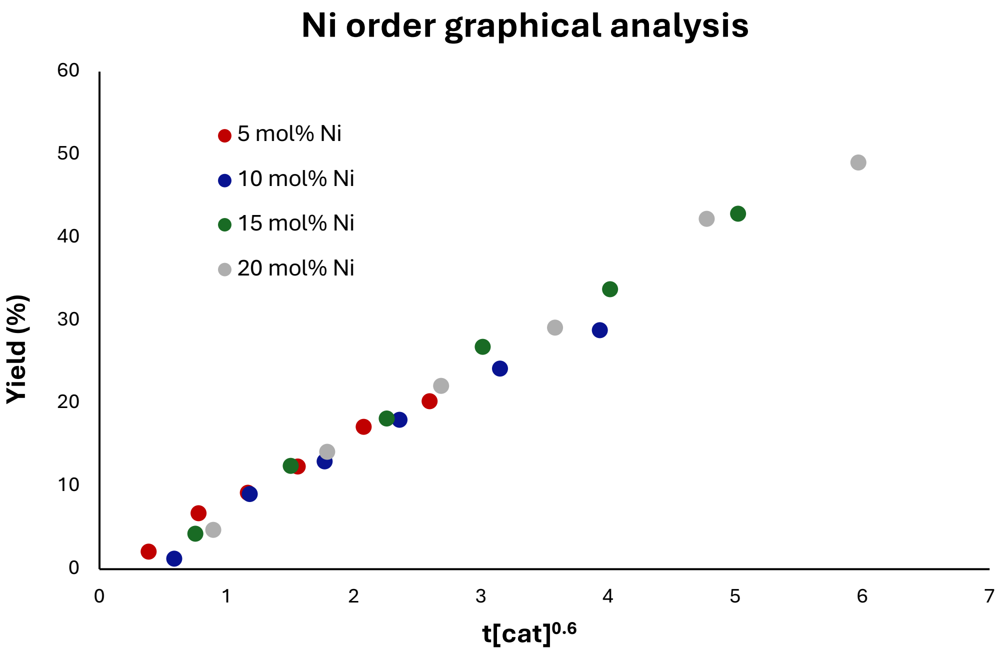

Figure S5: Yield *vs* $t\,[Ni(cod)_2]^{0.6}$ displaying 0.6 order with respect to $Ni(cod)_2$.[28]

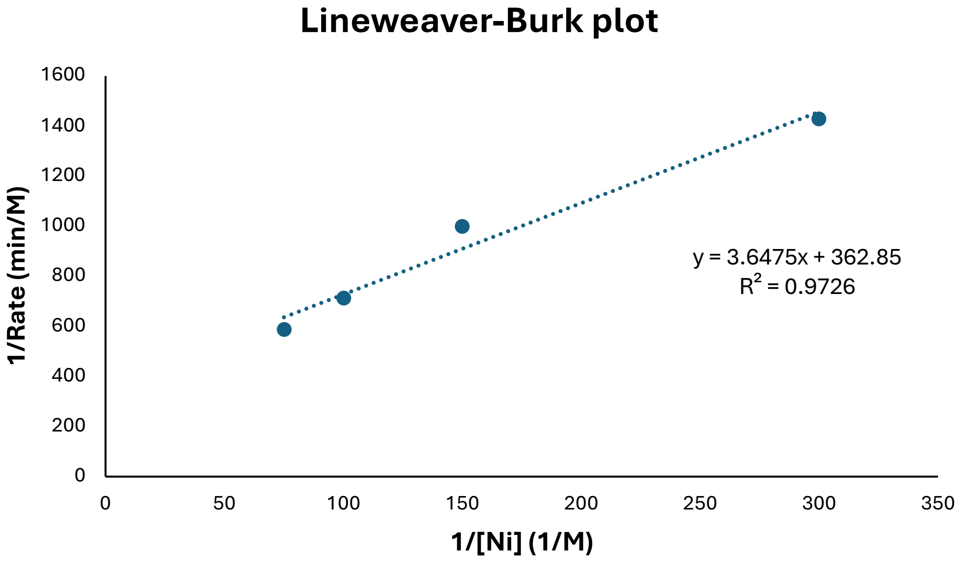

Figure S6: Lineweaver-Burk plot from raw data depicting saturation kinetic behavior with respect to Ni(cod)$_2$ concentration.

Experiment setup:

<u>Stock solution A:</u> (*S*)-*i*-Pr-PHOX (0.082 mmol, 30.8 mg) was dissolved in 2.5 mL xylenes (0.033 M). Subsequently, Ni(cod)$_2$ (0.082 mmol, 22.7 mg) was weighed into a separate 1-dram vial. Once (*S*)-*i*-Pr-PHOX is fully dissolved, the ligand solution was pipetted into the vial containing the Ni(cod)$_2$.

<u>Stock solution B:</u> Cyclopropyl phenyl ketone (0.75 mmol, 104 µL) was added and dissolved with 5.0 mL xylenes in a 20 mL scintillation vial. Then 3,5-bis(trifluoromethyl)benzonitrile (1.125 mmol, 186 µL), and diphenyl silane (1.5 mmol, 279 µL) were added.

Oven-dried Schlenk tubes were brought into the glovebox with septum stoppers, and 1.11 mL stock solution B was added to all four Schlenk tubes labeled: 5 mol% Ni, 10 mol% Ni, 15 mol% Ni, and 20 mol% Ni. Additional xylenes was added to ensure the total solvent volume was 2.25 mL (0.67 M): 1.05 mL (5 mol% Ni), 0.84 mL (10 mol% Ni), 0.64 mL (15 mol% Ni), and 0.43 mL (20 mol% Ni), followed by the addition of TMSOTf (33 µL) to each reaction vessel. Lastly, stock solution A was added: 0.20 mL (5 mol% Ni), 0.21 mL (10 mol% Ni), 0.61 mL (15 mol% Ni), and 0.82 mL (20 mol% Ni) making the total volume of every flask 2.25 mL. Once stock solution B, additional xylenes, TMSOTf, and stock solution A were mixed, they were immediately brought

out of the glovebox, cycled onto the Schlenk line, and lowered into the oil bath within 5-7 min. Aliquots were taken at the indicated times and worked up before ^{1}H NMR analysis. The yields of the product were determined by cyclopropyl phenyl ketone conversion from ^{1}H NMR analysis.

<u>Rate dependence with respect to cyclopropyl phenyl ketone:</u>

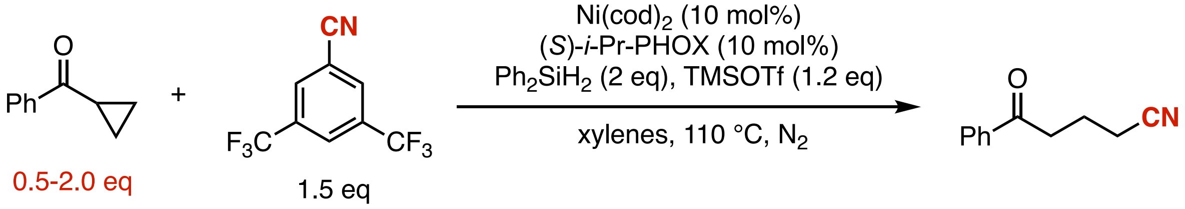

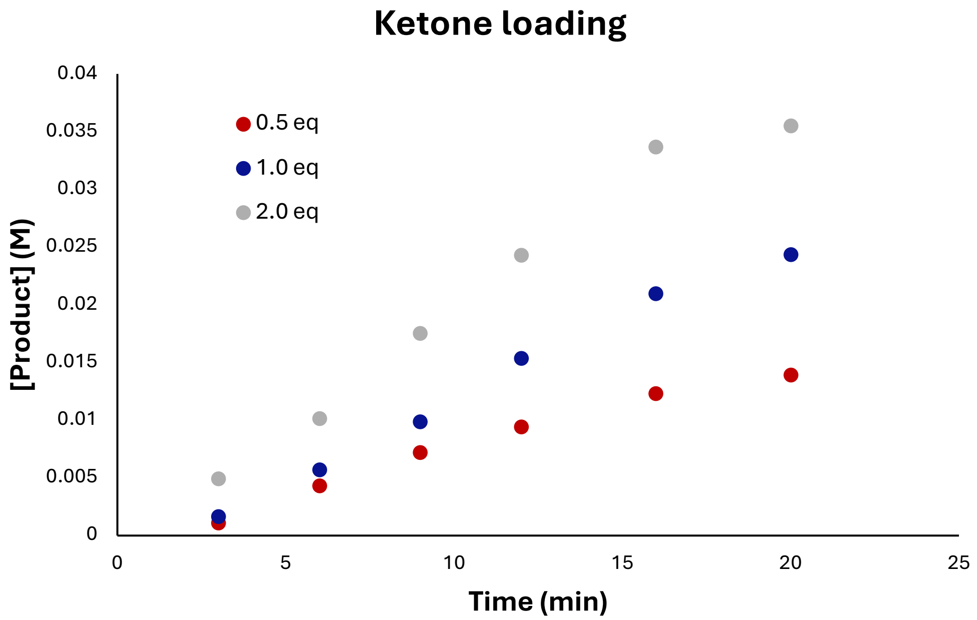

Figure S7: Reaction profiles for different ketone concentrations.

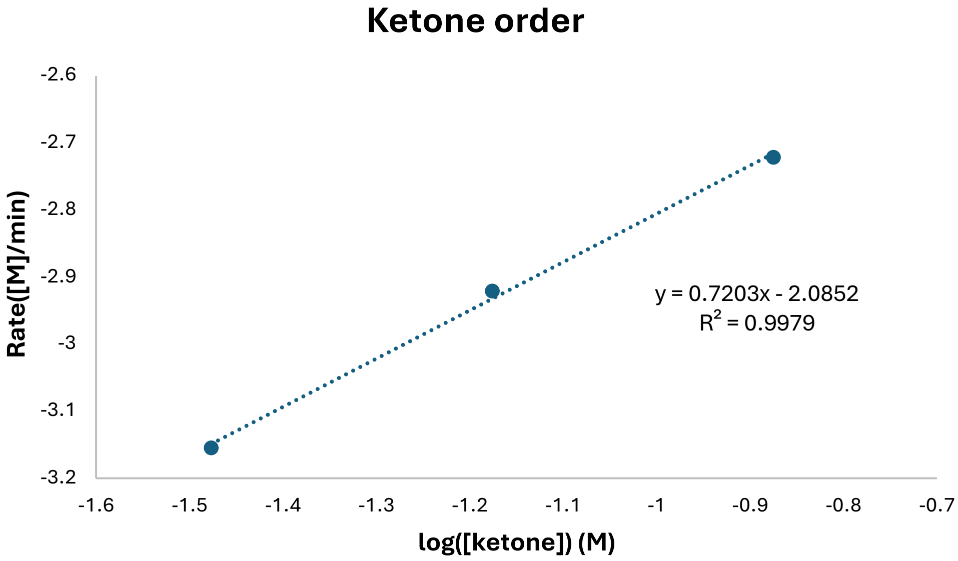

Figure S8: Logarithm of rate *vs* logarithm of ketone concentration displaying 0.7 order with respect to ketone.

Experiment setup:

Stock solution A: (*S*)-*i*-Pr-PHOX (0.060 mmol, 22.4 mg) was dissolved in 4.0 mL xylenes (0.15 M). Subsequently, $Ni(cod)_2$ (0.060 mmol, 16.5 mg) was weighed into a separate 1-dram vial. Once (*S*)-*i*-Pr-PHOX is fully dissolved, the ligand solution was pipetted into the vial containing the $Ni(cod)_2$.

Stock solution B: 3,5-bis(trifluoromethyl)benzonitrile (0.90 mmol, 148 µL) was added and dissolved with 4.0 mL xylenes in a 20 mL scintillation vial. Followed by the addition of diphenyl silane (1.2 mmol, 222 µL).

Oven-dried Schlenk tubes were brought into the glovebox with septum stoppers, and 1.10 mL stock solution B was added to all three Schlenk tubes labeled: 0.5 eq ketone, 1.0 eq ketone, and 2.0 eq ketone. Different amount of ketone were added to the reactions: 41 µL cyclopropyl phenyl ketone (2.0 eq), 21 µL (1.0 eq) and 10 µL (0.5 eq). Additional xylenes 0.25 mL was added to ensure the total solvent volume was 2.25 mL followed by the addition of TMSOTf (33 µL) to each reaction vessel. Lastly, 1.0 mL of stock solution A was added. Once stock solution B, ketone, additional xylenes, TMSOTf, and stock solution A were mixed, they were immediately brought out of the glovebox, cycled onto the Schlenk line, and lowered into the oil bath within 5-7 min.

Aliquots were taken at the indicated times and worked up before ^{1}H NMR analysis. The yields of the product were determined by cyclopropyl phenyl ketone conversion from ^{1}H NMR analysis.

Rate dependence with respect to benzonitrile:

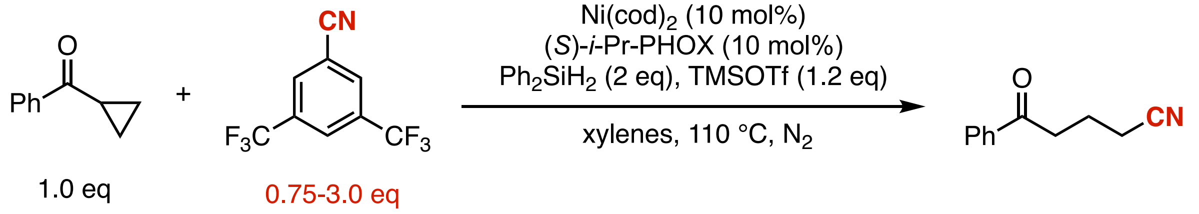

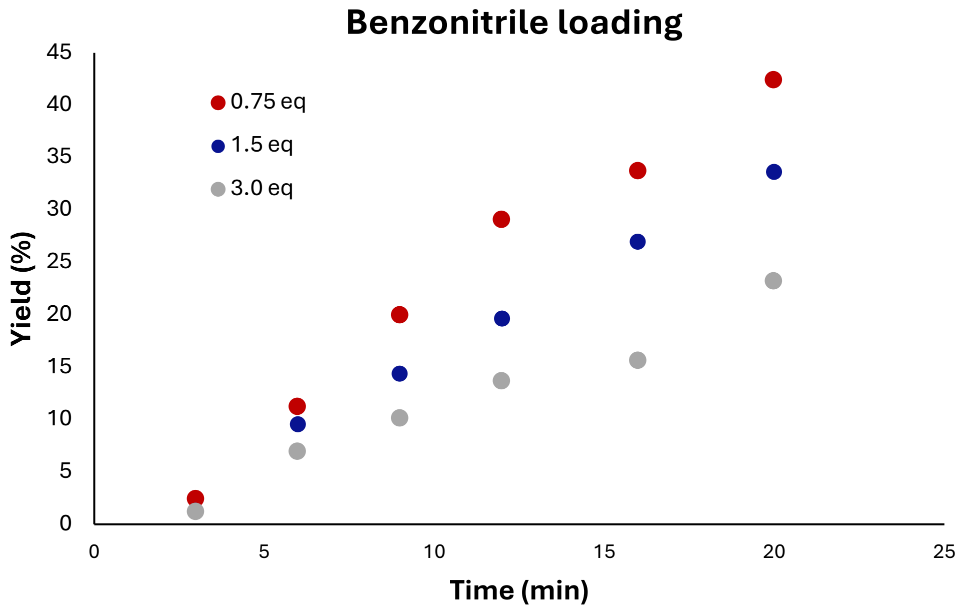

Figure S9: Reaction profiles for different benzonitrile concentrations.

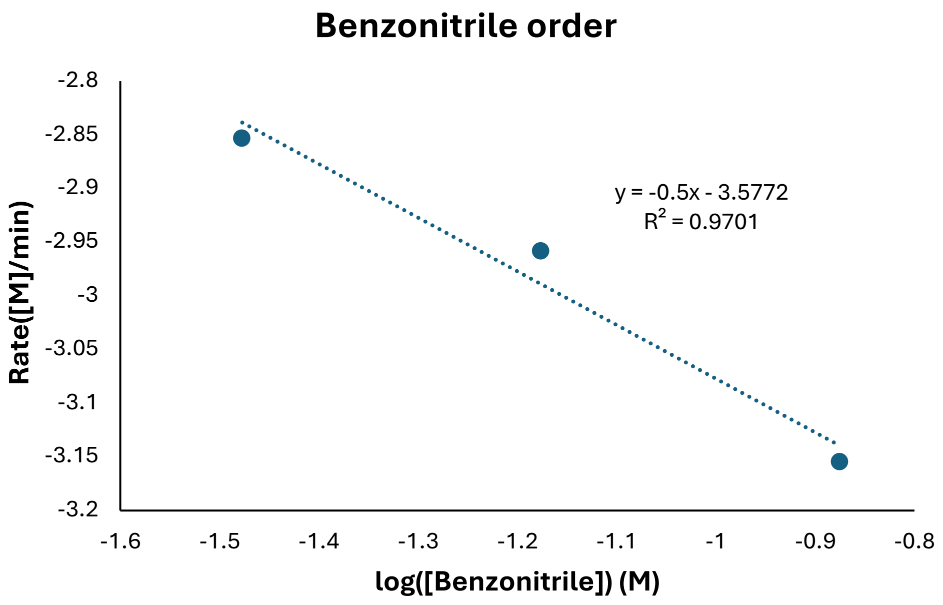

Figure S10: Logarithm of rate *vs* logarithm of benzonitrile concentration displaying -0.5 order with respect to benzonitrile.

Experiment setup:

Stock solution A: (*S*)-*i*-Pr-PHOX (0.060 mmol, 22.4 mg) was dissolved in 4.0 mL xylenes (0.15 M). Subsequently, $Ni(cod)_2$ (0.060 mmol, 16.5 mg) was weighed into a separate 1-dram vial. Once (*S*)-*i*-Pr-PHOX is fully dissolved, the ligand solution was pipetted into the vial containing the $Ni(cod)_2$.

Stock solution B: Cyclopropyl phenyl ketone (0.60 mmol, 83 μL) was added and dissolved with 4.0 mL xylenes in a 20 mL scintillation vial. Followed by the addition of diphenyl silane (1.2 mmol, 222 μL).

Oven-dried Schlenk tubes were brought into the glovebox with septum stoppers, and 1.08 mL stock solution B was added to all three Schlenk tubes labeled: 0.75 eq ArCN, 1.5 eq ArCN, and 2.0 eq ArCN. Different amount of benzonitrile were added to the reactions: 19 μL 3,5-bis(trifluoromethyl)benzonitrile (0.75 eq), 37 μL (1.5 eq) and 74 μL (3.0 eq). Additional xylenes 0.25 mL was added to ensure the total solvent volume was 2.25 mL followed by the addition of TMSOTf (33 μL) to each reaction vessel. Lastly, 1.0 mL of stock solution A was added. Once stock solution B, benzonitrile, additional xylenes, TMSOTf, and stock solution A were mixed,

they were immediately brought out of the glovebox, cycled onto the Schlenk line, and lowered into the oil bath within 5-7 min. Aliquots were taken at the indicated times and worked up before ^{1}H NMR analysis. The yields of the product were determined by cyclopropyl phenyl ketone conversion from ^{1}H NMR analysis.

Rate dependence with respect to silane:

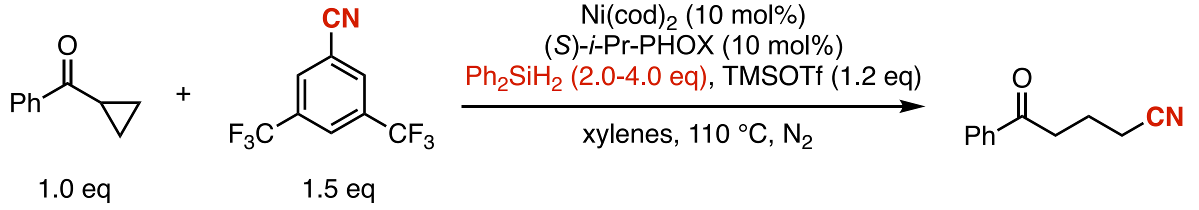

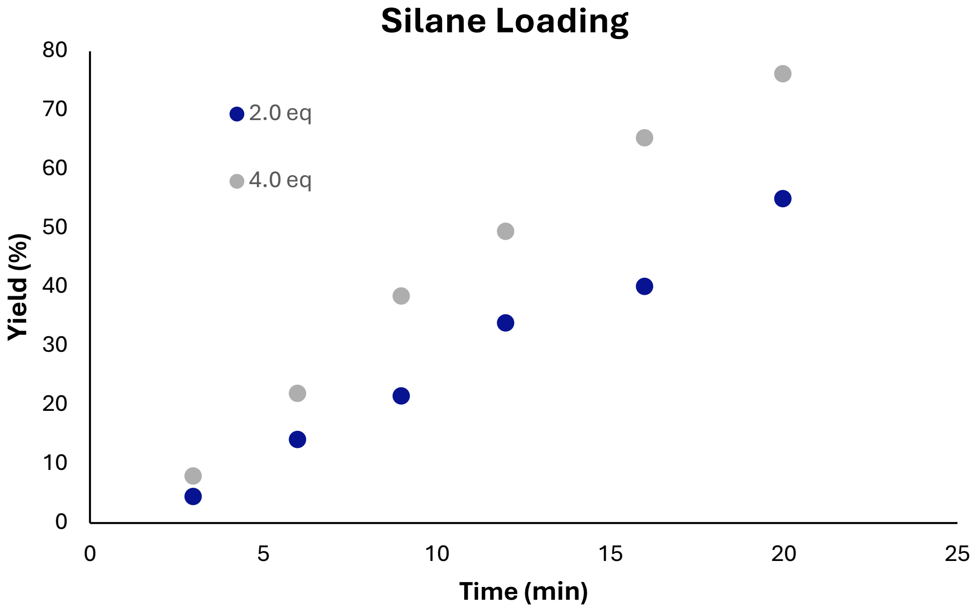

Figure S11: Reaction profiles for different silane concentrations.

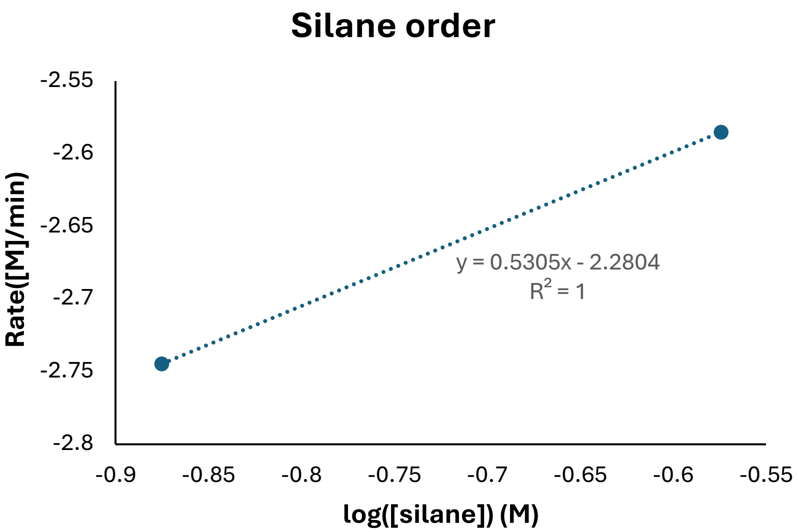

Figure S12: Logarithm of rate *vs* logarithm of silane concentration displaying 0.5 order with respect to silane.

Experiment setup:

<u>Stock solution A:</u> (*S*)-*i*-Pr-PHOX (0.045 mmol, 16.8 mg) was dissolved in 3.0 mL xylenes (0.15 M). Subsequently, $Ni(cod)_2$ (0.045 mmol, 12.4 mg) was weighed into a separate 1-dram vial. Once (*S*)-*i*-Pr-PHOX is fully dissolved, the ligand solution was pipetted into the vial containing the $Ni(cod)_2$.

<u>Stock solution B:</u> Cyclopropyl phenyl ketone (0.45 mmol, 62 μL) was added and dissolved with 3.0 mL xylenes in a 20 mL scintillation vial. Then followed by the 3,5-bis(trifluoromethyl) benzonitrile (0.675 mmol, 111 μL).

Oven-dried Schlenk tubes were brought into the glovebox with septum stoppers, and 1.06 mL stock solution B was added to two Schlenk tubes labeled: 2.0 eq silane, and 4.0 eq silane. Different amount of silane were added to the reactions: diphenyl silane 56 μL (2.0 eq) and 111 μL (4.0 eq). Additional xylenes 0.25 mL was added to ensure the total solvent volume was 2.25 mL followed by the addition of TMSOTf (33 μL) to each reaction vessel. Lastly, 1.0 mL of stock solution A was added. Once stock solution B, silane, additional xylenes, TMSOTf, and stock solution A were mixed, they were immediately brought out of the glovebox, cycled onto the Schlenk line, and lowered into the oil bath within 5-7 min. Aliquots were taken at the indicated times and worked

up before ^{1}H NMR analysis. The yields of the product were determined by cyclopropyl phenyl ketone conversion from ^{1}H NMR analysis.

Rate dependence with respect to Lewis acid (LA):

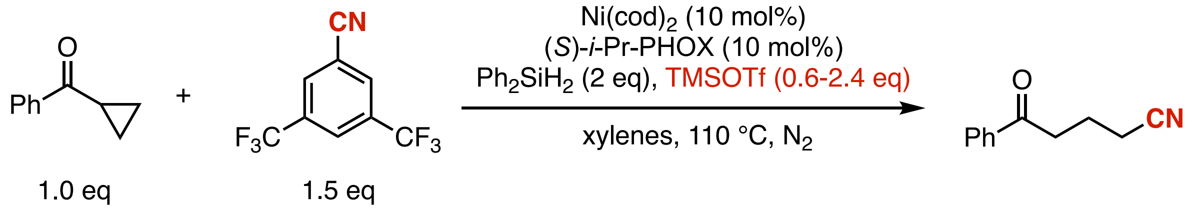

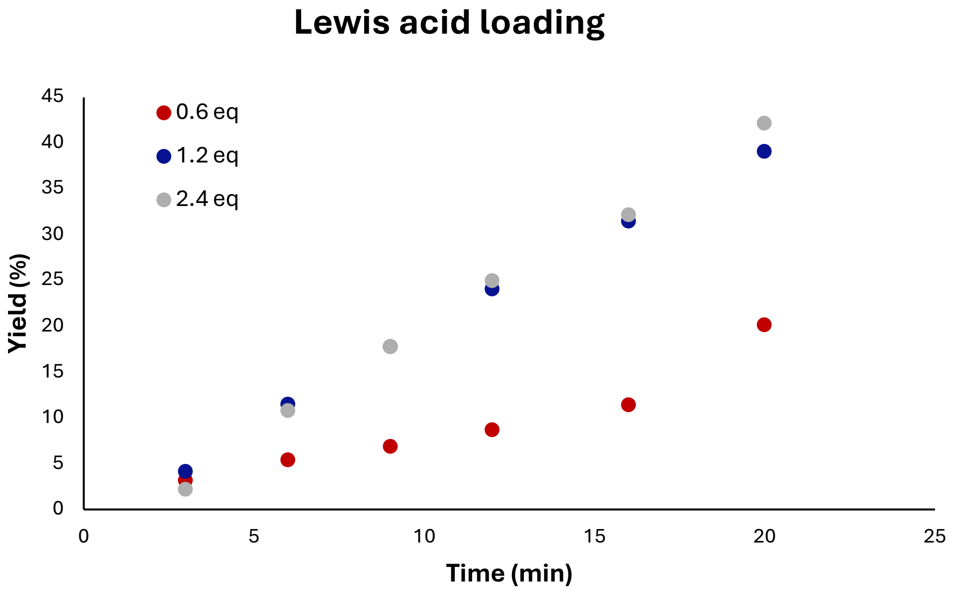

Figure S13: Reaction profiles for different Lewis acid concentrations.

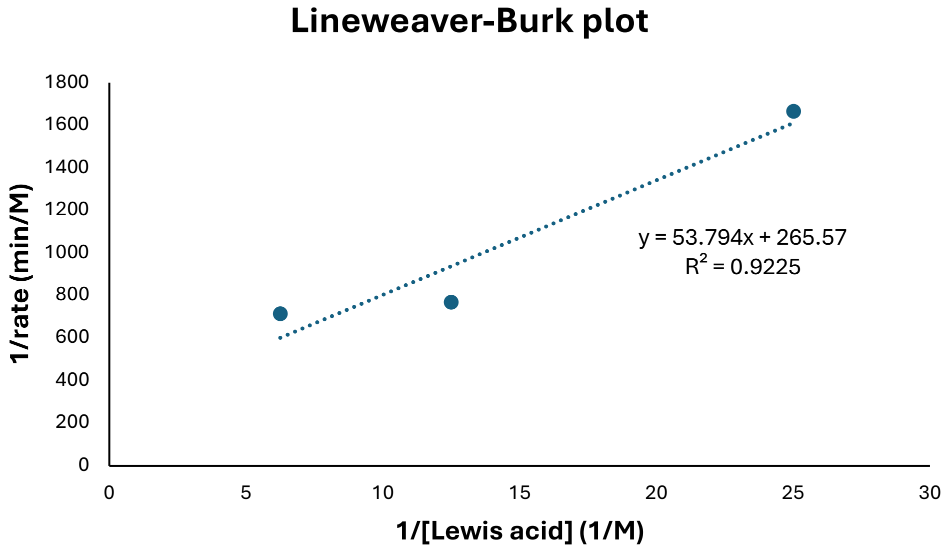

Figure S14: Lineweaver-Burk plot from raw data depicting saturation kinetic behavior with respect to Lewis acid concentration.

Experiment setup:

Stock solution A: (*S*)-*i*-Pr-PHOX (0.060 mmol, 22.4 mg) was dissolved in 4.0 mL xylenes (0.15 M). Subsequently, Ni(cod)$_2$ (0.060 mmol, 16.5 mg) was weighed into a separate 1-dram vial. Once (*S*)-*i*-Pr-PHOX is fully dissolved, the ligand solution was pipetted into the vial containing the Ni(cod)$_2$.

Stock solution B: Cyclopropyl phenyl ketone (0.60 mmol, 83 μL) was added and dissolved with 4.0 mL xylenes in a 20 mL scintillation vial. Then 3,5-bis(trifluoromethyl)benzonitrile (0.90 mmol, 148 μL), and diphenyl silane (1.2 mmol, 222 μL) were added.

Oven-dried Schlenk tubes were brought into the glovebox with septum stoppers, and 1.06 mL stock solution B was added to three Schlenk tubes labeled: 0.6 eq LA, 1.2 eq LA, and 2.4 eq LA. Additional xylenes 0.25 mL was added to ensure the total solvent volume was 2.25 mL. Different amounts of Lewis acid were added to the reactions: TMSOTf 16 μL (0.6 eq), 33 μL (1.2 eq) and 65 μL (2.4 eq). Lastly, 1.0 mL of stock solution A was added. Once stock solution B, additional xylenes, TMSOTf, and stock solution A were mixed, they were immediately brought out of the glovebox, cycled onto the Schlenk line, and lowered into the oil bath within 5-7 min. Aliquots

were taken at the indicated times and worked up before ^{1}H NMR analysis. The yields of the product were determined by cyclopropyl phenyl ketone conversion from ^{1}H NMR analysis.

6. **Reactions using TMSCN**

Reacting **1a** with TMSCN as CN source, with and without silane

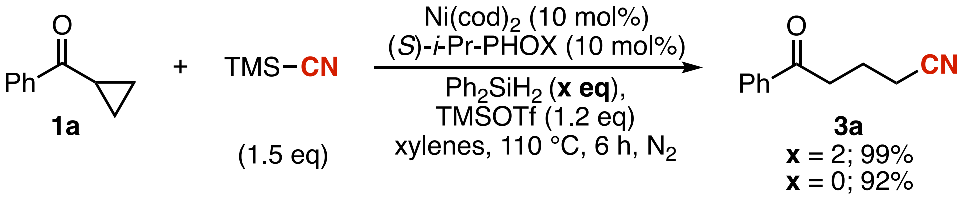

Reaction carried out following a modified general procedure A, $Ni(cod)_2$ (0.015 mmol), ligand (0.015 mmol), **1a** (0.15 mmol), TMSCN (0.225 mmol), diphenyl silane (0.3 mmol), TMSOTf (0.18 mmol), and xylenes (2.25 mL). The reaction was heated to 110 °C for 6 h. The formation of **3a** in >95% yield was measured by NMR yield with dimethyl fumarate as an NMR standard. The same reaction was carried out in the absence of diphenyl silane. The formation of **3a** in 92% yield was measured by NMR yield with dimethyl fumarate as an NMR standard.

Comparison of the kinetic profile of the model reaction and using TMSCN as CN source

TMSCN reaction:

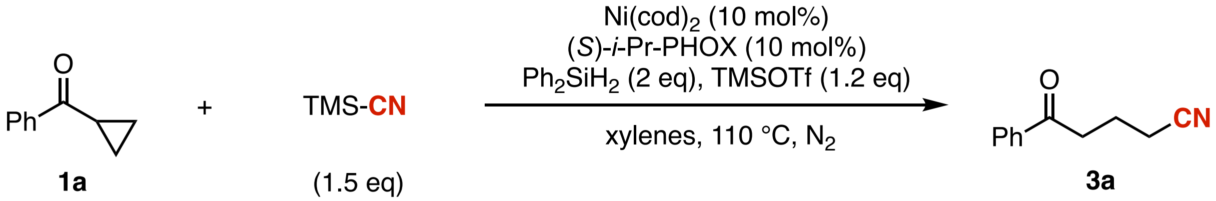

An oven-dried Schlenk tube was brought into the glovebox with a septum stopper. Reaction was carried out following a modified general procedure **A**, $Ni(cod)_2$ (0.015 mmol), ligand (0.015 mmol), **1a** (0.15 mmol), TMSCN (0.225 mmol), diphenyl silane (0.3 mmol), TMSOTf (0.18 mmol), and xylenes (2.25 mL). Were mixed into the Schlenk tube, and brought immediately out of the glovebox, cycled onto the Schlenk line, and lowered into the oil bath within 5-7 min.

Model reaction:

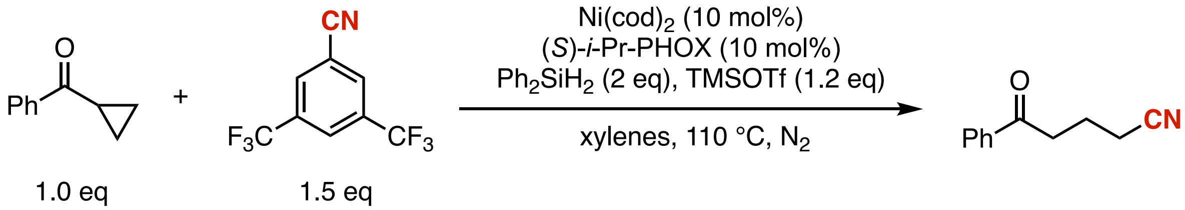

The model reaction was set up according to the procedure detailed in Section 2.

Both reactions were run in parallel in the same oil bath and aliquots (100 µL) were taken at 3, 6, 9, 12, and 20 minutes. Each aliquot was immediately injected and mixed into a prefilled 1-dram vial with a PTFE-lined cap containing DCM (250 µL) and H_2O (2-3 drops) before ^{1}H NMR analysis. The yields of the product were determined by cyclopropyl phenyl ketone conversion from ^{1}H NMR analysis.

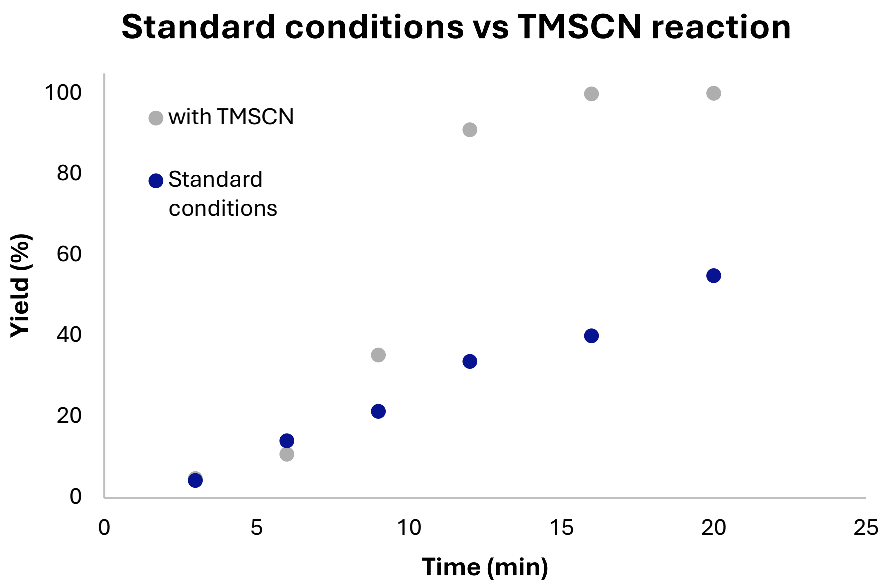

Figure S15: Comparison of the reaction profile for the standard conditions with a reaction using TMSCN as a CN source.

Comparison of the reaction of different cyclopropyl ketones with TMSCN as CN source to the optimized conditions

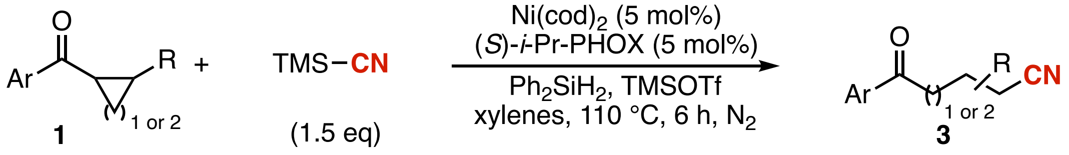

The reactions with TMSCN were carried out following a modified general procedure A, $Ni(cod)_2$ (0.015 mmol), ligand (0.015 mmol), **1** (0.15 mmol), TMSCN (0.225 mmol), diphenyl silane (0.3 mmol), TMSOTf (0.18 mmol), and xylenes (2.25 mL) were used. The reaction was heated to 110 °C for 6 h. The reaction was quenched with DCM and water, filtered through a silica plug, and then analyzed by ^{1}H NMR with dimethyl fumarate as an internal standard to determine the yield.

Table S3: Comparison of the use of TMSCN and the standard conditions

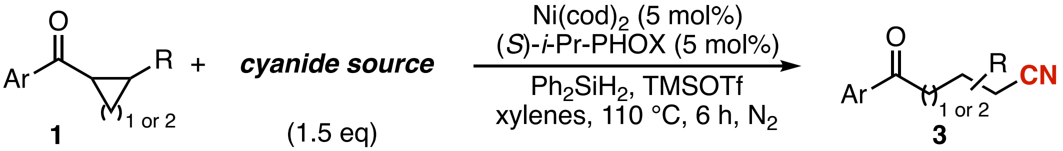

Entry	Product	Yield of 3 optimal conditions:**w/TMSCN**
1	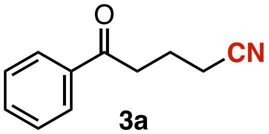 **3a**	95% : 99%
2	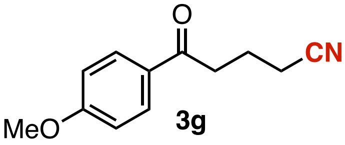 **3g**	71% : 70%
3	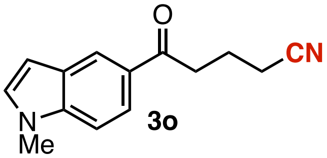 **3o**	47% : 76%
4	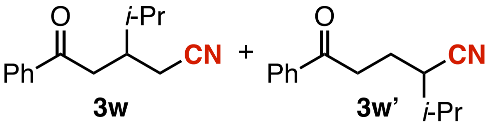 **3w** + **3w’**	70% (68:32 r.r.) : 68% (66:34 r.r.)[a]
5	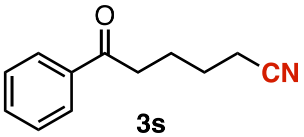 **3s**	41% : 44%

Reactions carried out following general procedure A and the procedure just described. All yields were determined by ^{1}H NMR using dimethyl fumarate as an NMR standard. [a] The products were purified by column chromatography prior to quantification.

7. NMR experiments of stability of reaction components

Reaction of Ph_2SiH_2 and TMSOTf in presence and absence of Ni, monitored by NMR

In a N_2-filled glovebox, Ph_2SiH_2 (7 μL, 0.038 mmol, 1 eq) and TMSOTf (8 μL, 0.044 mmol, 1.1 eq) were directly added to an NMR tube by syringe, followed by 0.55 mL toluene-d_8, and the NMR tube was capped with a septum. A ^{1}H NMR spectrum was acquired (red spectrum). Then the NMR tube was placed in a 100 °C oil bath, and an argon balloon was added. The reaction was heated for one hour before ^{1}H NMR analysis (blue spectrum).

Another solution of the same reagents and concentrations was also prepared and kept at room temperature overnight before ^{1}H NMR analysis (green spectrum).

All these spectra show no reaction between Ph_2SiH_2 and TMSOTf.

In the glovebox Ni(cod)$_2$ (8.3 mg) and (*S*)-*i*-Pr-PHOX (11.2 mg) were weighed into a vial and dissolved in toluene-d_8 (2 mL). 0.25 mL of this Ni stock solution (0.0075 mmol Ni, 20 mol%) were added to the NMR tube containing the previously detailed mixture: Ph_2SiH_2 (7 μL, 0.038 mmol, 1 eq) and TMSOTf (8 μL, 0.044 mmol, 1.1 eq) in 0.55 mL toluene-d_8. The mixture was vortexed prior to analysis. Approximately 5 minutes after mixing Ni(cod)$_2$, (*S*)-*i*-Pr-PHOX (11.2 mg), Ph_2SiH_2, and TMSOTf at room temperature, a series of new silicon-ligated species are observed with the new major species presenting a single at -0.02 ppm (purple spectrum). The data indicates scrambling among the silicon-bonded species in the presence of Ni.

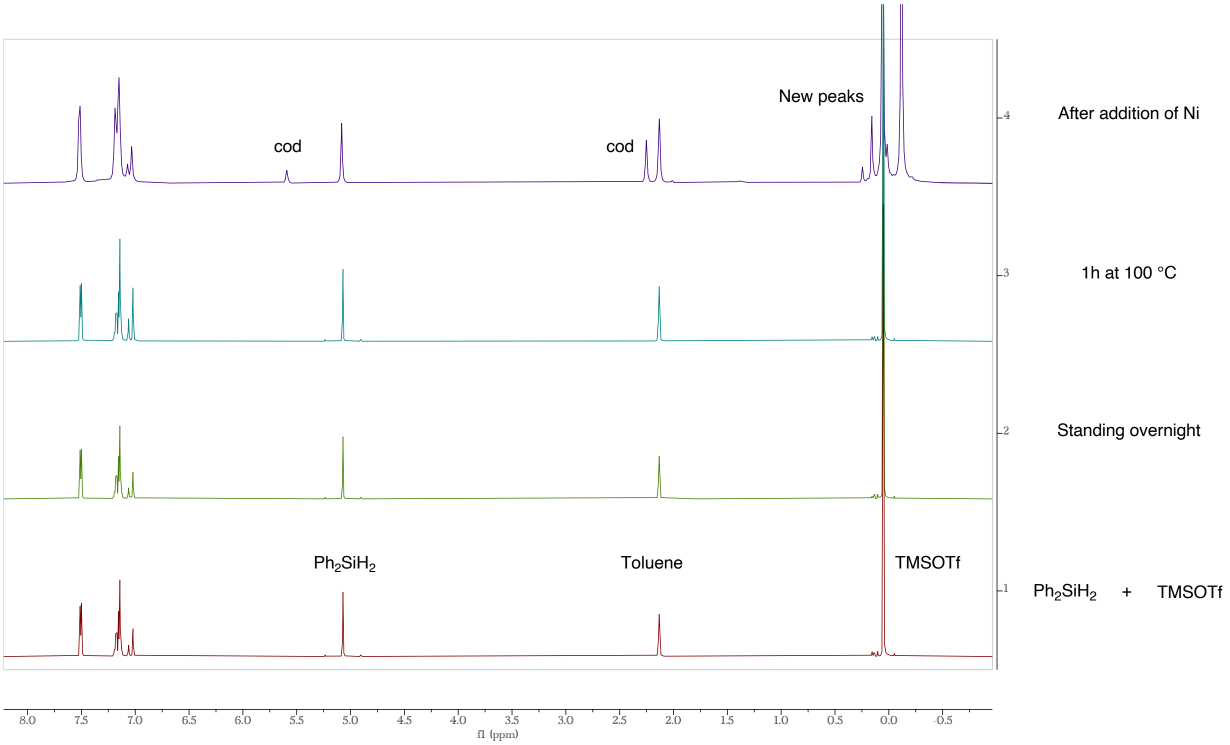

Figure S16: ^{1}H NMR (600 MHz, Tol) spectra of the reaction mixture. From the bottom up: Ph_2SiH_2 and TMSOTf 5 min after mixing (red spectrum), same mixture after standing overnight (green spectrum), same mixture after 1 hour heating at 100 °C (blue spectrum), 5 min after the addition of nickel and ligand stock solution (purple spectrum).

<u>Reaction in the absence of cyclopropyl ketone monitored by NMR</u>

In a N_2-filled glovebox, Ni(cod)$_2$ (27.5 mg, 0.10 mmol) and (*S*)-*i*-Pr-PHOX (37.3 mg, 0.10 mmol) were weighed into a dram vial and dissolved in benzene-d$_6$ (1.9 mL). Then **2e** (247 µL, 1.5 mmol) and Ph_2SiH_2 (371 µL, 2.0 mmol) were added via syringe giving 2.5 mL of solution. The blood-red solution (stock A) was shaken and allowed to dissolve for 30 min, after which it was added to NMR tubes in 0.5 mL increments which were sealed with septa caps and electrical tape (0.02 mmol Ni each). One NMR tube was used to conduct the experiment, and a second tube was spiked with two drops of TMSCN for signal assignment. The peak at –0.10 ppm was assigned to TMSCN (second spectrum from the bottom). To ensure that the TMSCN signal did not shift in the presence of the Lewis acid, TMSOTf (43.5 µL, 0.24 mmol) was added to the solution, and ^{1}H NMR spectra were recorded 5 minutes and 1 hour after mixing. No significant shift of the TMSCN signal (in the presence of TMSOTf) was observed in either of these spectral time points (as shown in the bottom spectrum).

For the reaction conducted in the absence of cyclopropyl ketone, TMSOTf (43.5 µL, 0.24 mmol) was added to stock solution A, and ^{1}H NMR spectra were recorded after 5 minutes, 15 minutes, and 1 hour; the mixture was kept room temperature for this experiment. As the reaction proceeds, the formation of TMSCN is observed, as evidenced by the appearance and growth of the signal at –0.10 ppm.

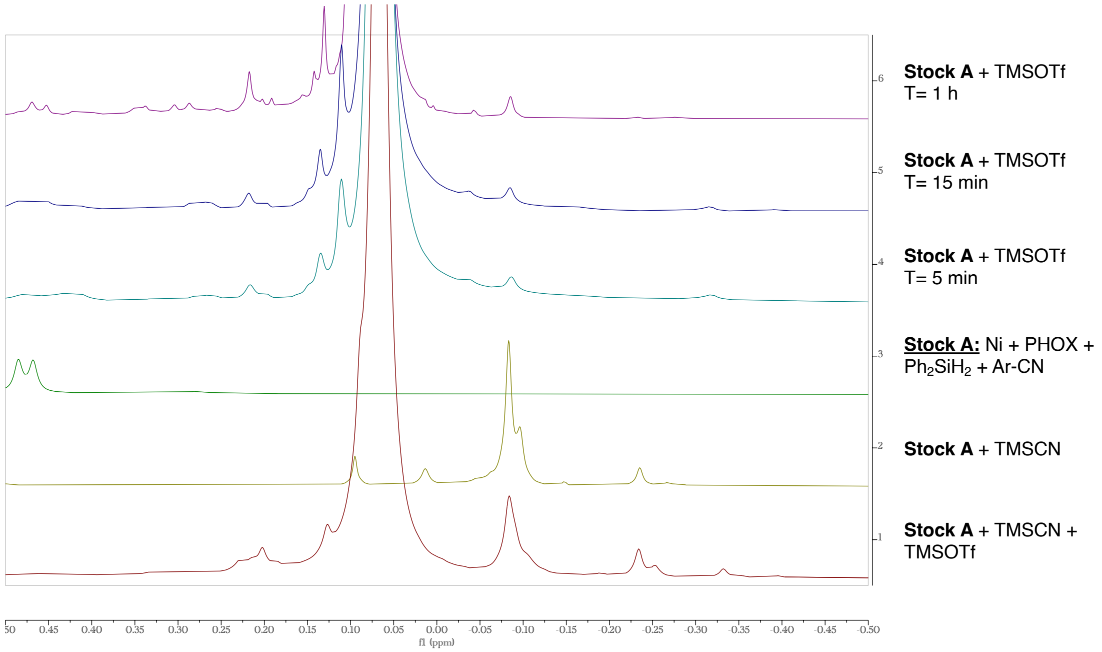

Figure S17: ^{1}H NMR (400 MHz, C_6D_6). From the bottom up: stock A spiked with TMSCN and treated with TMSOTf 5 min after mixing (red spectrum), same mixture before the addition of TMSOTf (yellow spectrum), stock A with no additions (green spectrum) same mixture after 5 min after the addition of TMSOTf (turquoise spectrum), after 15 min (dark purple spectrum) and 1 hour (light purple spectrum).

8. Bimetallic bridging cyanide complex 4: synthesis and reactivity

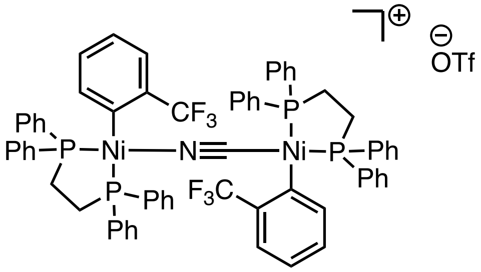

4

$Ni(cod)_2$ (220 mg, 0.8 mmol) and bis(diphenylphosphino)ethane (318.7 mg, 0.8 mmol) were weighed into a 20 ml scintillation vial and suspended in 16 ml of toluene. 2-(trifluoromethyl)benzonitrile (1.060 mL, 8.0 mmol) was then added and the suspension turned from orange to wine red. A PTFE-coated stir bar was added, and the solution was allowed to stir at room temperature to fully dissolve over 25 min. Once dissolved, TMSOTf (108.5 µL, 0.6 mmol) was added and the color darkened to a red-brown. After 15 min of stirring, a dark green solid precipitated, and the reaction was allowed to continue to react overnight. The solids were then vacuum filtered and collected on a glass frit, then washed with toluene then pentane. The crude complex was scraped off the frit and purified. Purification was accomplished by suspending in THF and heating to 70 °C, followed by hot filtration through a glass frit. The solids were collected and the process was repeated. The combined filtrates were partially concentrated under vacuum to reduce the volume, and precipitating at room temperature by adding pentane. The solid was collected on a frit, washed with minimal THF and pentane, then dried under vacuum to afford 355.6 mg (65% yield) of **4** as a yellow solid. **^{1}H NMR** (600 MHz, CD_3CN) δ 8.17 – 6.83 (brm, 41H), 6.76 – 6.55 (brm, 2H), 6.55 – 6.36 (brm, 5H), 3.03 – 2.25 (brm, 5H), 1.47 – 1.19 (brm, 3H). **^{13}C{^{1}H} NMR** (151 MHz, CD_3CN) δ 158.4 – 157.7 (m), 156.6 – 154.7 (m), 137.4 (d, J = 38.7 Hz), 136.5 – 135.9 (m), 135.6 (t, J = 10.4 Hz), 134.1 (dd, J = 25.1, 11.3 Hz), 133.7 (t, J = 10.6 Hz), 133.5 (d, J = 17.7 Hz), 132.5 (t, J = 9.8 Hz), 132.3 (d, J = 8.6 Hz), 132.0 (s), 131.6 (s), 131.4 (s), 130.6 (s), 130.4 (d, J = 10.5 Hz), 130.25 – 129.95 (m), 129.7 (s), 129.4 (brs), 129.0 (brs), 128.9 (dd, J = 13.8, 10.2 Hz), 128.7 (brs), 128.3 (brs), 127.5 (brs), 127.0 (d, J = 11.8 Hz), 125.90 (dd, J = 39.9, 18.1 Hz), 125.2 (d, J = 11.3 Hz), 123.9 (d, J = 60.7 Hz), 122.18 (q, J = 321.2 Hz), 29.2 (dd, J = 30.6, 17.5 Hz), 28.0 (dd, J= 29.1, 16.3 Hz), 23.5 (dd, J = 29.1, 13.9 Hz), 21.7 (dd, J = 29.7, 11.1 Hz). **^{19}F NMR** (376 MHz, CD_3CN) δ -61.02, -62.28, -80.69. **^{31}P{^{1}H} NMR** (162 MHz,

CD_3CN) δ 53.40 (dd, J = 32.4, 4.9 Hz), 52.44 (dd, J = 33.6, 5.0 Hz), 51.82 (d, J = 33.3 Hz), 44.95 (d, J = 32.4 Hz).

Monitoring complex **4** formation by NMR

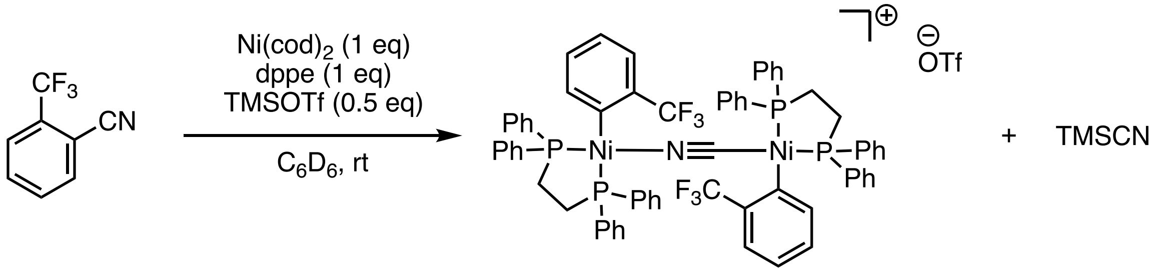

Inside a N_2 filled glovebox, $Ni(cod)_2$ (137.5 mg, 0.5 mmol) and dppe (199.2 mg, 0.5 mmol) were weighed out into a vial 20 mL scintillation vial and dissolved in benzene-d_6 (2 mL). Once dissolved, 2-(trifluoromethyl)benzonitrile (330 µL, 2.5 mmol) was added and the color darkened. 0.5 mL (0.1 mmol Ni) of the stock solution was then transferred to up to 5 NMR tubes. One tube was spiked with a drop of TMSCN and set aside. The tubes were then capped with septa caps and removed from the box. Before the addition of TMSOTf, ^{1}H NMR spectra were taken of the spiked NMR sample and the stock samples, then TMSOTf (9 µL, 0.05 mmol) was injected into the NMR tube, which was shaken and quickly injected into the NMR probe. ^{1}H spectra were acquired roughly every 1.5 min showing the formation of TMSCN under the reaction conditions.

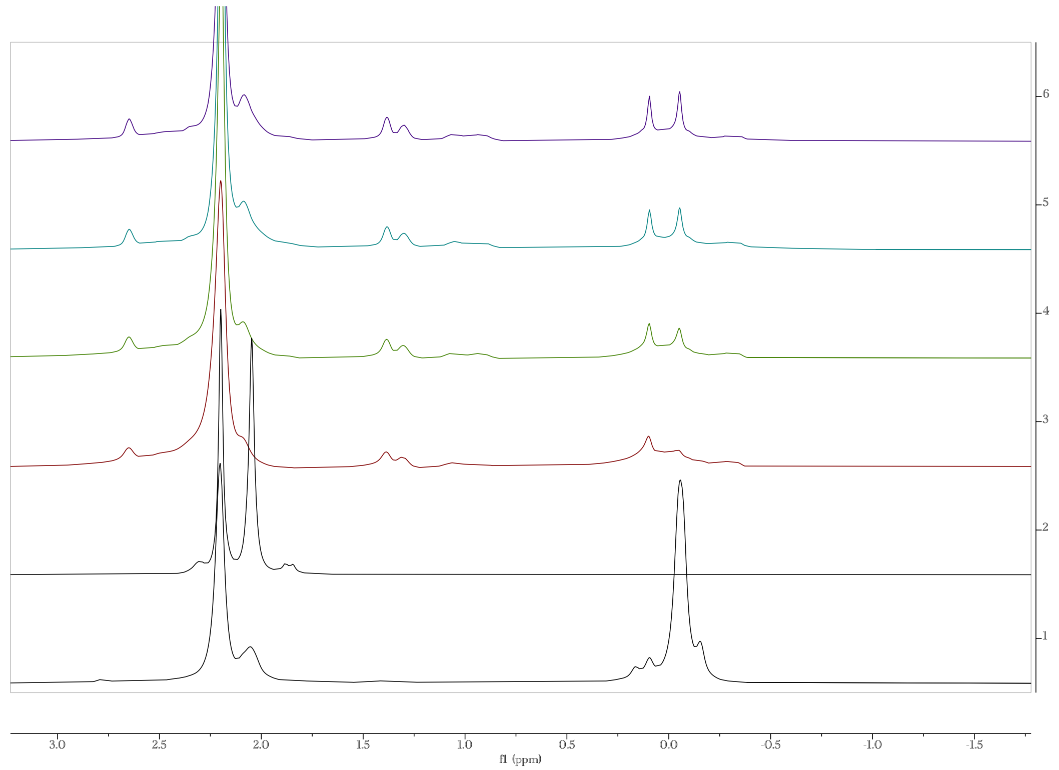

Figure S18: ^1H NMR (400 MHz, C_6D_6) spectra over time. From the bottom up: (1) stock solution spiked with TMSCN, (2) clean stock solution, (3) stock solution TMSOTf added, 2 min post addition, (3) 3.5 min post addition, (4) 5 min post addition, (5) 6.5 min post addition, (6) 8 min post addition.

<u>Reaction of **complex 4** with TMSOTf, monitored by NMR</u>

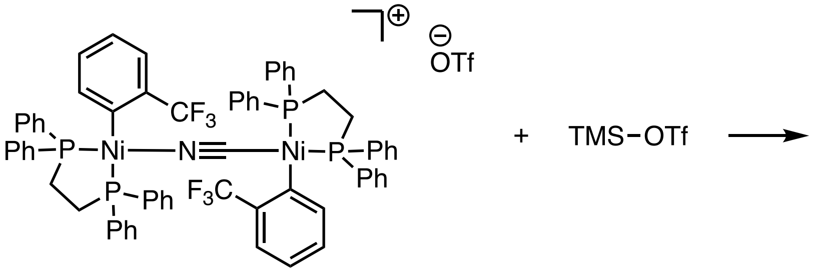

In a nitrogen filled glovebox complex **4** (138 mg, 0.1 mmol) was dissolved in acetonitrile-d$_3$ (1 mL). 0.5 mL (0.05 mmol **4**) of that solution were added to an NMR tube. Subsequently, TMSOTf (9 µL, 0.05 mmol) was added, and the NMR tube capped with a septum. Fluorine and phosphorus spectra were acquired, then the NMR tube was placed in a 70 °C oil bath, bearing an argon balloon. The reaction was tracked over time, showing immediate reaction, and complete conversion of the complex to a new species upon heating. Decomposition is observed after heating overnight.

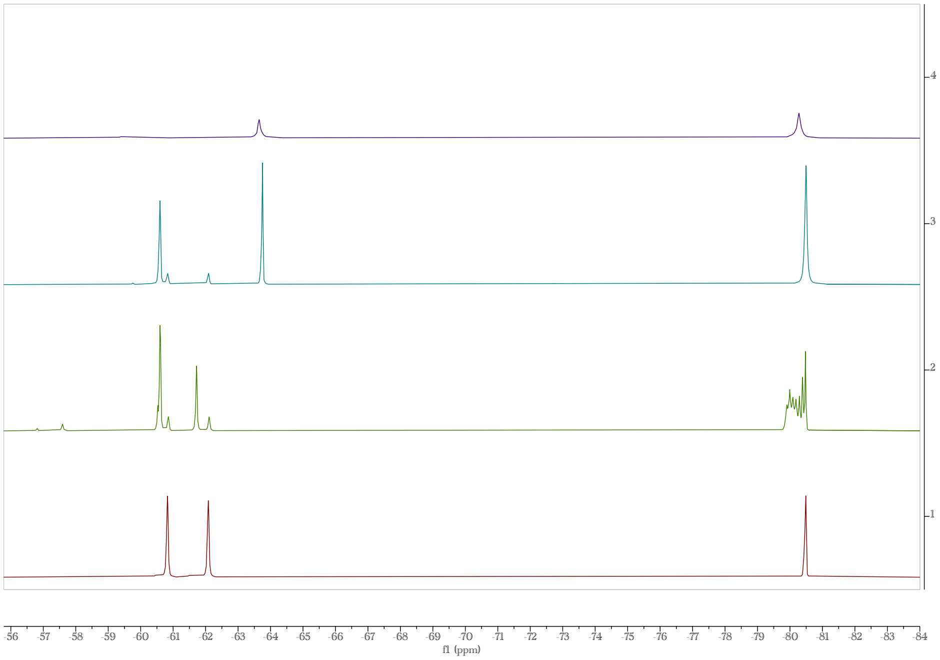

Figure S19: ^{19}F NMR (376 MHz, CD_3CN) spectra of the reaction mixture. From the bottom up: (1) complex **4** soution, (2) reaction with TMSOTf, 5 min post addition, (3) after 30 min heating at 70 °C, (4) overnight at 70 °C.

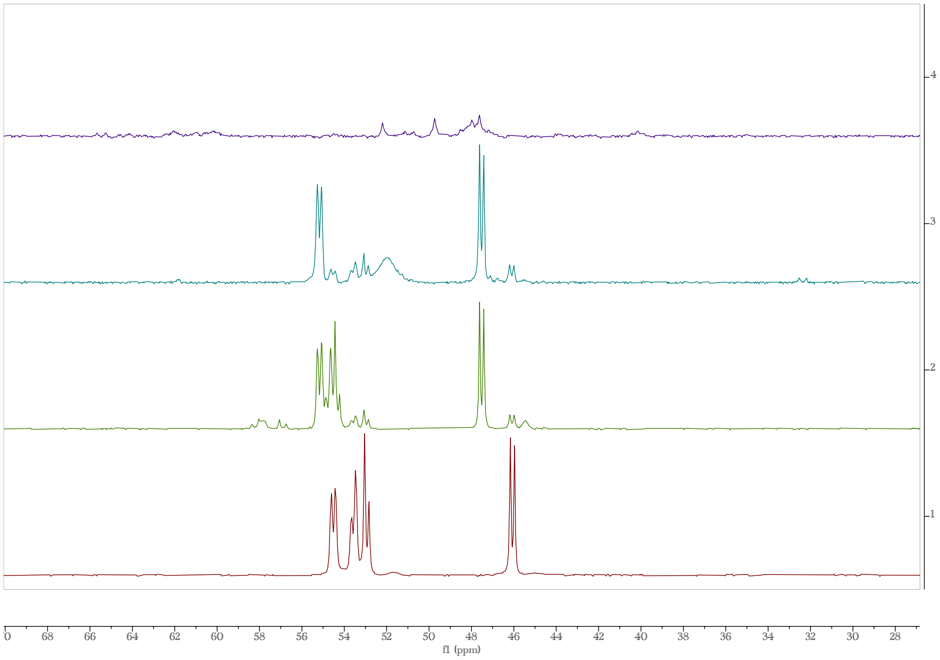

Figure S20: ^{31}P NMR (162 MHz, CD_3CN) spectra of the reaction mixture. From the bottom up: (1) pure complex, (2) reaction with TMSOTf, 5 min post addition, (3) after 30 min heating at 70 °C, (4) overnight at 70 °C.

Reaction of complex **4** with Ph_2SiH_2, monitored by NMR

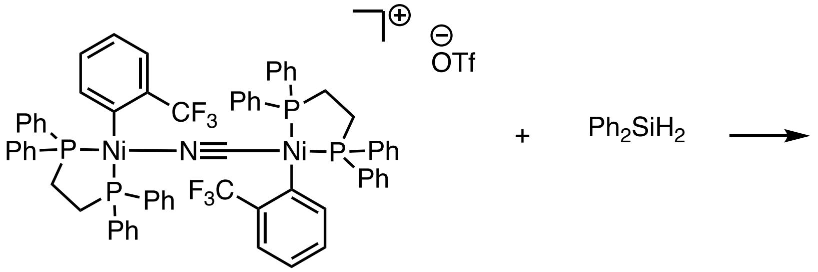

Complex **4** (138 mg, 0.1 mmol) was dissolved in acetonitrile-d_3 (1 mL). To an NMR tube was added 0.5 mL (0.05 mmol **4**) of the stock solution, and Ph_2SiH_2 (19 µL, 0.1 mmol) was added, and the NMR tube capped with a septum. Fluorine and phosphorus spectra were acquired, then the NMR tube was placed in a 70 °C oil bath, and an argon balloon was added. The reaction was tracked over time showing no reaction at room temperature and slow conversion to multiple new species at 70 °C. Two new species are observed in the ^{19}F NMR, as opposed to only one when reacting with TMSOTf. The major species in the ^{19}F NMR is the same when reacting with TMSOTf or Ph_2SiH_2.

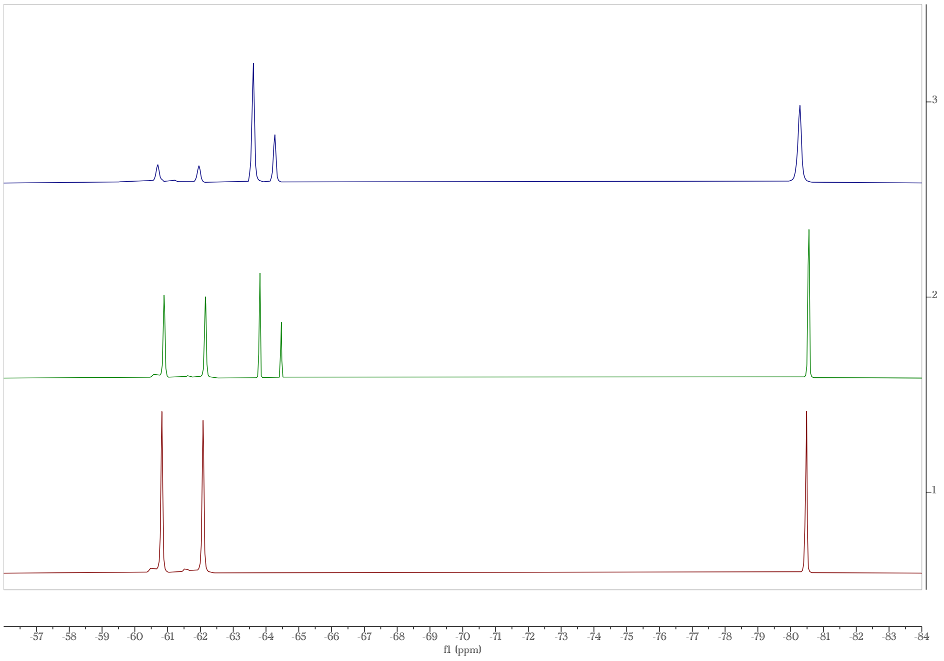

Figure S21: ^{19}F NMR (376 MHz, CD_3CN) spectra of the reaction mixture. From the bottom up: (1) pure complex, no reaction with Ph_2SiH_2 5 min post addition, (2) after 30 min heating at 70 °C, (3) overnight at 70 °C.

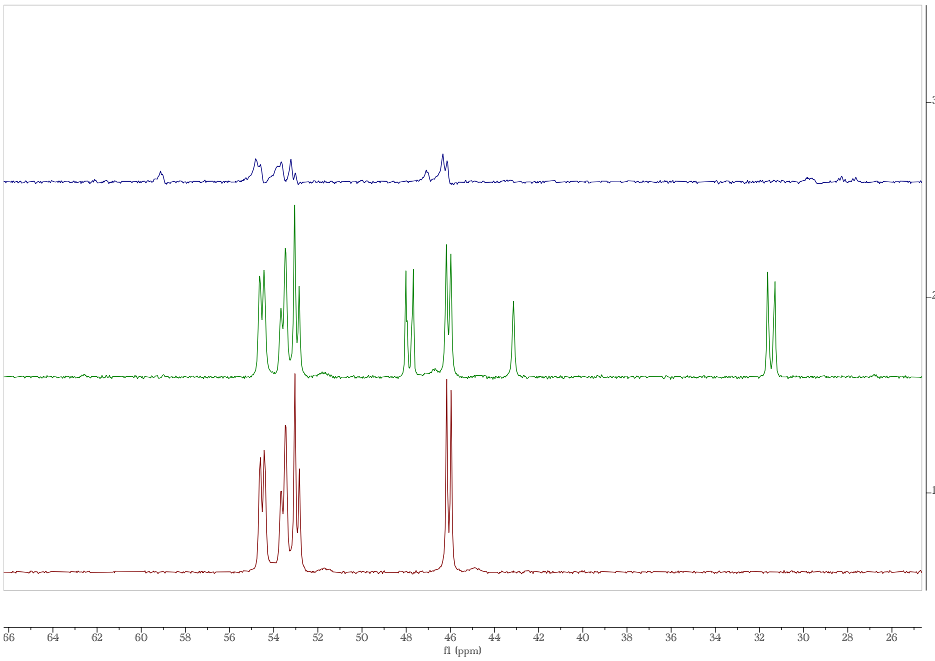

Figure S22: ^{31}P NMR (162 MHz, CD_3CN) spectra of the reaction mixture. From the bottom up: (1) pure complex, no reaction with Ph_2SiH_2 5 min post addition, (2) after 30 min heating at 70 °C, (3) overnight at 70 °C.

Utilizing **4** as precatalyst under standard conditions

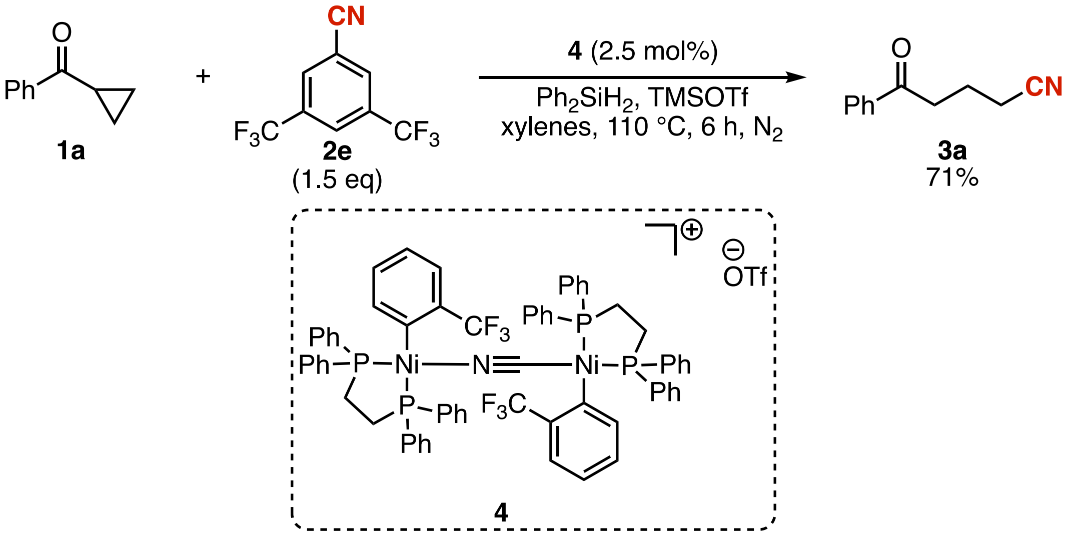

In a N_2 filled glove box, **4** (5.2 mg, 0.00375 mmol, 2.5 mol%) was charged to a 2-dram vial equipped with a PTFE-coated lid and stir bar, then suspended in xylenes (1 mL). In a separate 1-dram vial a 2x stock solution of the reaction components was created by adding xylenes (2.5 mL), **1a** (41.4 µL, 0.30 mmol), **2e** (0.45 mmol, 74.2 µL), diphenyl silane (0.600 mmol, 111.4 µL), and

TMSOTf (0.360 mmol, 32.7 μL). The mixture was gently swirled after each addition. At this stage, 1.25 mL (0.15 mmol **1a**) of the reaction reagents stock solution was added to the suspension of **4**. The reaction vial was then capped, electrical taped, and placed in an aluminum block heated to 110 °C for 6 h, with stirring. Solids slowly dissolved over the first 30 min of the reaction. A 71% yield of **3a** was determined by ^{1}H NMR using dimethyl fumarate as an internal standard.

<u>Utilizing **4** as precatalyst with TMSCN as CN source</u>

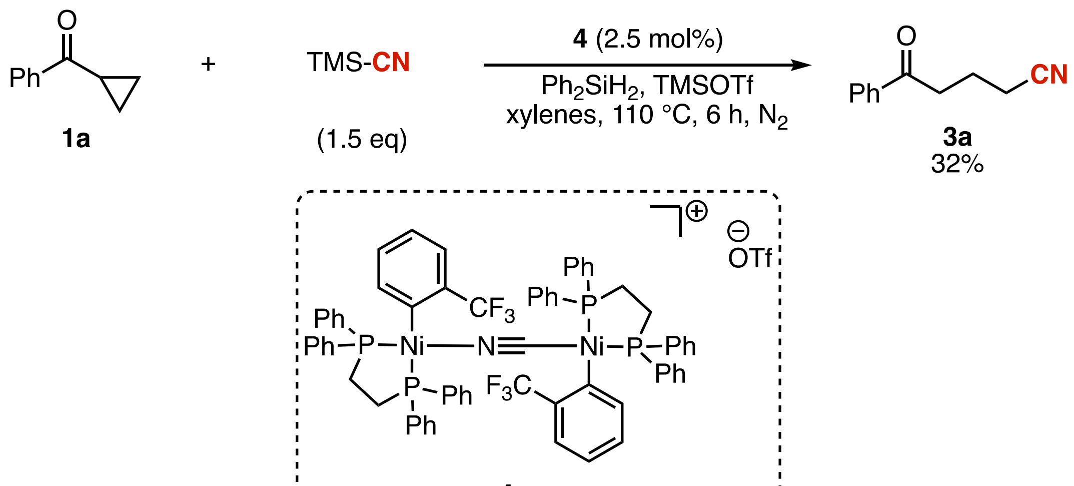

4

In a N_2 filled glove box, **4** (5.2 mg, 0.00375 mmol, 2.5 mol%) was charged to a 2-dram vial equipped with a PTFE-coated lid and stir bar, then suspended in xylenes (1 mL). In a separate 1-dram vial a 2x stock solution of the reaction components was created by adding xylenes (2.5 mL), **1a** (41.4 μL, 0.30 mmol), TMSCN (0.45 mmol, 56.2 μL), diphenyl silane (0.600 mmol, 111.4 μL), and TMSOTf (0.360 mmol, 32.7 μL). The mixture was gently swirled after each addition. At this stage, 1.25 mL (0.15 mmol **1a**) of the reaction reagents stock solution was added to the suspension of **4** which dissolved and began to turn yellow. The reaction vial was then capped, electrical taped, and placed in an aluminum block heated to 110 °C for 6 h, with stirring. Solids slowly dissolved over the first 30 min of the reaction. The product distribution was analyzed by ^{1}H NMR using dimethyl fumarate as an internal standard showing the formation of **3a** in 32% and butyrophenone in 23% yield.

9. X-ray crystallographic data

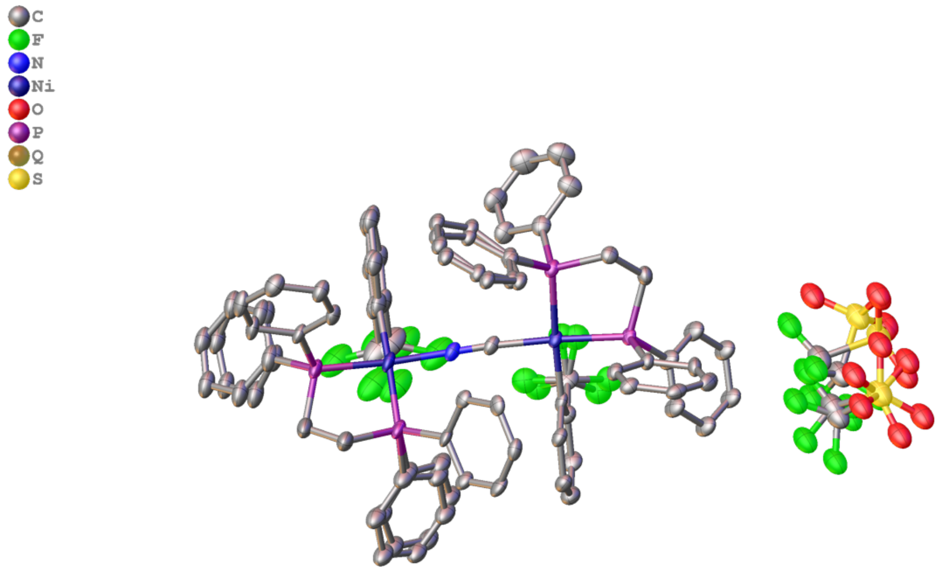

X-ray structure of **4**

Chemical formula: $C_{68}H_{56}F_9NNi_2O_3P_4S$

Data collection

Single crystals suitable for X-ray diffraction were grown by vapor diffusion of pentane into acetonitrile. A yellow crystal (block, approximate dimensions 0.13 × 0.12 × 0.11 mm^3) was placed onto the tip of a MiTeGen pin and mounted on a Bruker Venture D8 diffractometer equipped with a PhotonIII detector at 100.00 K. The data collection was carried out using Cu Kα radiation (λ = 1.54178 Å, IμS micro-source) with a frame time of 3 seconds and a detector distance of 40 mm. A collection strategy was calculated and complete data to a resolution of 0.82 Å were collected. The frames were integrated with the Bruker SAINT[23] software package using a narrow-frame algorithm to 0.83 Å resolution. Data were corrected for absorption effects using the multi-scan method (SADABS).[24] Please refer to Table S4 for additional crystal and refinement information.

Structure solution and refinement

The space group P-1 was determined based on intensity statistics and systematic absences. The structure was solved using the SHELX suite of programs[25] and refined using full-matrix least-squares on F^2 within the OLEX2 suite.[26] An intrinsic phasing solution was calculated, which provided most non-hydrogen atoms from the E-map. Full-matrix least squares / difference Fourier

cycles were performed, which located the remaining non-hydrogen atoms. All non-hydrogen atoms were refined with anisotropic displacement parameters. The hydrogen atoms were placed in ideal positions and refined as riding atoms with relative isotropic displacement parameters. Disorder was modelled for several aromatic rings and for the CF_3 groups. The crystals suffer from solvent loss and disordered solvent molecules (acetonitrile and pentane) are present, as well as disordered triflates. The triflates were modelled by splitting the crystallographic occupancy into 4 parts. The disorder of solvent molecules could not be modelled and a solvent mask that accounted for 30 electrons per asymmetric unit was used.[27] The final full matrix least squares refinement converged to R1 = 0.1085 and wR2 = 0.2410 (F^2, all data). The goodness-of-fit was 1.133. Based on the final model, the calculated density was 1.350 g/cm^3 and F(000), 1418 e$^-$.

Table S4. Crystal data and structure refinement for **4**.

Empirical formula	C68.01 H56 F9.05 N Ni2 O3.05 P4 S1.01	
Formula weight	1381.73	
Crystal color, shape, size	yellow block, 0.13 × 0.12 × 0.11 mm^3	
Temperature	100.00 K	
Wavelength	1.54178 Å	
Crystal system, space group	Triclinic, P-1	
Unit cell dimensions	a = 12.7731(6) Å	α = 95.769(3)°.
	b = 13.3159(7) Å	β = 96.793(3)°.
	c = 20.2755(10) Å	γ = 93.067(3)°.
Volume	3399.5(3) Å^3	
Z	2	
Density (calculated)	1.350 g/cm^3	
Absorption coefficient	2.461 mm^{-1}	
F(000)	1418	
**Data collection**		
Diffractometer	Bruker D8 Venture	
Theta range for data collection	2.207 to 68.243°.	
Index ranges	-15<=h<=15, -16<=k<=16, -24<=l<=24	
Reflections collected	90616	
Independent reflections	12429 [R_{int} = 0.0636]	
Observed Reflections	10870	
Completeness to theta = 67.679°	99.8 %	
**Solution and Refinement**		
Absorption correction	Semi-empirical from equivalents	

Max. and min. transmission	0.7533 and 0.6392
Solution	Intrinsic methods
Refinement method	Full-matrix least-squares on F^2
Weighting scheme	$w = [\sigma^2 Fo^2 + AP^2 + BP]^{-1}$, with
	$P = (Fo^2 + 2 Fc^2)/3$, A = 0.00, B = 39.2
Data / restraints / parameters	12429 / 519 / 715
Goodness-of-fit on F^2	1.133
Final R indices [$I>2\sigma(I)$]	R1 = 0.1085, wR2 = 0.2331
R indices (all data)	R1 = 0.1206, wR2 = 0.2410
Largest diff. peak and hole	1.556 and -1.162 e.Å^{-3}

10. Product characterization

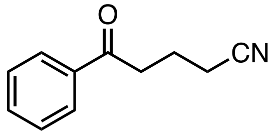

5-oxo-5-phenylpentanenitrile (**3a**)

Following general procedure A, modified that **1a** (0.15 mmol, 21.9 mg), 5% Ni(cod)$_2$, and 5% (*S*)-*i*-Pr-PHOX were employed. The reaction was heated to 110 °C for 6 h. The resulting residue was purified by flash chromatography (silica, 10-20% gradient EtOAc in hexanes, **R*f*** = 0.24 in 20% EtOAc in hexanes) to afford 24.7 mg (95% yield) of **3a** as a yellow solid (**mp** = 37.8-38.2 °C). **^{1}H NMR** (600 MHz, $CDCl_3$) δ 7.99 – 7.94 (m, 2H), 7.62 – 7.56 (m, 1H), 7.48 (t, *J* = 7.8 Hz, 2H), 3.18 (t, *J* = 6.9 Hz, 2H), 2.53 (t, *J* = 6.9 Hz, 2H), 2.12 (p, *J* = 6.9 Hz, 2H). **^{13}C{^{1}H} NMR** (151 MHz, $CDCl_3$) δ 198.3, 136.5, 133.6, 128.9, 128.0, 119.5, 36.4, 19.8, 16.8. **HRMS** (ESI-TOF) m/z calculated for $C_{11}H_{11}NO$ $(M+H)^+$: 174.0914 found 174.0906. The characterization data matches a previous report.[29]

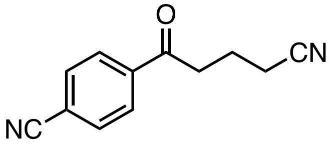

4-(4-cyanobutanoyl)benzonitrile (**3b**)

Following general procedure A, **1b** (0.15 mmol, 25.7 mg) was employed. The reaction was heated to 110 °C for 6 h. The resulting residue was purified by flash chromatography (silica, 0-30% gradient EtOAc in hexanes, **R*f*** = 0.16 in 30% EtOAc in hexanes) to afford 24.9 mg (83% yield) of **3b** as a white solid (**mp** = 97.5-100.4 °C). **^{1}H NMR** (400 MHz, $CDCl_3$) δ 8.12 – 8.02 (m, 2H), 7.83 – 7.76 (m, 2H), 3.20 (t, *J* = 6.8 Hz, 2H), 2.55 (t, *J* = 6.8 Hz, 2H), 2.14 (p, *J* = 6.8 Hz, 2H). **^{13}C{^{1}H} NMR** (151 MHz, $CDCl_3$) δ 196.9, 139.3, 132.8, 128.5, 119.3, 117.9, 116.9, 36.8, 19.5, 16.7. **HRMS** (ESI-TOF) m/z calculated for $C_{12}H_{10}N_2O$ $(M+H)^+$: 197.0721 found 197.0724. The characterization data matches a previous report.[30]

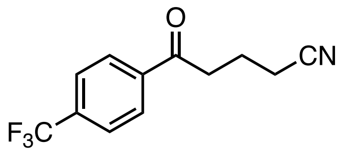

5-oxo-5-(4-(trifluoromethyl)phenyl)pentanenitrile (**3c**)

Following general procedure A, **1c** (0.15 mmol, 32.1 mg) was employed. The reaction was heated to 110 °C for 6 h. The resulting residue was purified by flash chromatography (silica, 10-15% gradient EtOAc in hexanes, **R*f*** = 0.13 in 10% EtOAc in hexanes) to afford 23.4 mg (65% yield) of **3c** as a pale yellow oil. **^{1}H NMR** (400 MHz, $CDCl_3$) δ 8.07 (d, *J* = 8.2 Hz, 2H), 7.75 (d, *J* = 8.2 Hz, 2H), 3.21 (t, *J* = 6.9 Hz, 2H), 2.54 (t, *J* = 6.9 Hz, 2H), 2.14 (p, *J* = 6.9 Hz, 2H). **^{13}C{^{1}H} NMR** (101 MHz, $CDCl_3$) δ 197.3, 139.1, 134.9 (q, *J* = 31.7 Hz), 128.5, 126.0 (q, *J* = 3.8 Hz), 123.6 (q, *J* = 272.9 Hz), 119.3, 36.8, 19.6, 16.7. **^{19}F NMR** (376 MHz, $CDCl_3$) δ -64.16. **HRMS** (ESI-TOF) m/z calculated for $C_{12}H_{10}F_3NO$ $(M-H)^-$: 240.0642 found 240.0644. The characterization data matches a previous report.[29]

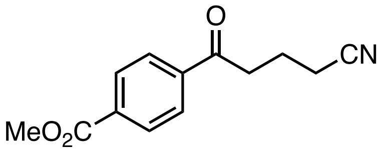

methyl 4-(4-cyanobutanoyl)benzoate (**3d**)

Following general procedure A, **1d** (0.15 mmol, 30.6 mg) was employed. The reaction was heated to 110 °C for 6 h. The resulting residue was purified by flash chromatography (silica, 20% EtOAc in hexanes, **R*f*** = 0.24 in 30% EtOAc in hexanes) to afford 20.7 mg (60% yield) of **3d** as a white solid (**mp** = 64.6-67.1 °C). **^{1}H NMR** (400 MHz, $CDCl_3$) δ 8.16 – 8.09 (m, 2H), 8.04 – 7.97 (m, 2H), 3.94 (s, 3H), 3.20 (t, *J* = 6.9 Hz, 2H), 2.53 (t, *J* = 6.9 Hz, 2H), 2.12 (p, *J* = 6.9 Hz, 2H). **^{13}C{^{1}H} NMR** (101 MHz, $CDCl_3$) δ 197.8, 166.2, 139.6, 134.3, 130.1, 128.0, 119.4, 52.6, 36.8, 19.7, 16.7. **HRMS** (ESI-TOF) m/z calculated for $C_{13}H_{13}NO_3$ $(M-H)^-$: 230.0823 found 230.0816. The characterization data matches a previous report.[31]

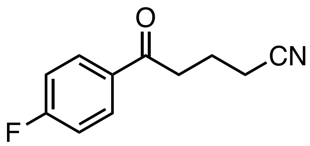

5-(4-fluorophenyl)-5-oxopentanenitrile (**3e**)

Following general procedure A, **1e** (0.15 mmol, 24.6 mg) was employed. The reaction was heated to 110 °C for 6 h. The resulting residue was purified by flash chromatography (silica, 10-12% gradient EtOAc in hexanes, **R*f*** = 0.23 in 20% EtOAc in hexanes) to afford 19.7 mg (69% yield) of **3e** as a pale yellow solid (**mp** = 50.6-53.1 °C). **^{1}H NMR** (400 MHz, $CDCl_3$) δ 8.04 – 7.94 (m, 2H), 7.20 – 7.09 (m, 2H), 3.15 (t, *J* = 6.9 Hz, 2H), 2.52 (t, *J* = 6.9 Hz, 2H), 2.11 (p, *J* = 6.9 Hz, 2H). **^{13}C{^{1}H} NMR** (101 MHz, $CDCl_3$) δ 196.6, 166.1 (d, *J* = 255.5 Hz), 133.0 (d, *J* = 3.0 Hz), 130.8 (d, *J* = 9.3 Hz), 119.5, 116.0 (d, *J* = 22.0 Hz), 36.3, 19.8, 16.8.**^{19}F NMR** (376 MHz, $CDCl_3$) δ -106.32 – -106.76 (m). **HRMS** (ESI-TOF) m/z calculated for $C_{11}H_{10}FNO$ $(M-H)^-$: 190.0674 found 190.0667. The characterization data matches a previous report.[29]

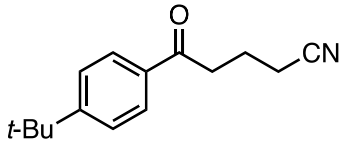

5-(4-(*tert*-butyl)phenyl)-5-oxopentanenitrile (**3f**)

Following general procedure A, **1f** (0.15 mmol, 30.3 mg) was employed. The reaction was heated to 110 °C for 6 h. The resulting residue was purified by flash chromatography (silica, 5-10% gradient EtOAc in hexanes, **R*f*** = 0.16 in 10% EtOAc in hexanes) to afford 21.7 mg (63% yield) of **3f** as a yellow solid (**mp** = 53.3-54.9 °C). **^{1}H NMR** (600 MHz, $CDCl_3$) δ 7.93 – 7.88 (m, 2H), 7.49 (d, *J* = 8.2 Hz, 2H), 3.16 (t, *J* = 6.9 Hz, 2H), 2.52 (t, *J* = 6.9 Hz, 2H), 2.11 (p, *J* = 6.9 Hz, 2H), 1.34 (s, 9H). **^{13}C{^{1}H} NMR** (151 MHz, $CDCl_3$) δ 197.9, 157.4, 134.0, 128.1, 125.8, 119.6, 36.3, 35.3, 31.2, 19.9, 16.8. **HRMS** (ESI-TOF) m/z calculated for $C_{15}H_{19}NO$ $(M-H)^-$: 230.1539 found 230.1531. The characterization data matches a previous report.[29]

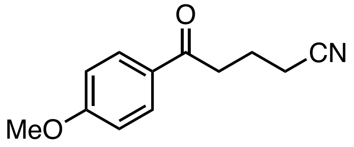

5-(4-methoxyphenyl)-5-oxopentanenitrile (**3g**)

Following general procedure A, **1g** (0.172 mmol, 30.3 mg) was employed. The reaction was heated to 110 °C for 6 h. The resulting residue was purified by flash chromatography (silica, gradient 0% to 20% EtOAc in hexanes, **R*f*** = 0.3 in 20% EtOAc in hexanes) to afford 21.7 mg (71% yield) of **3g** as a light brown solid (**mp** = 69.4-71.7 °C). **^{1}H NMR** (600 MHz, $CDCl_3$) δ 7.94 (d, *J* = 8.9 Hz, 2H), 6.94 (d, *J* = 8.9 Hz, 2H), 3.88 (s, 3H), 3.13 (t, *J* = 6.9 Hz, 2H), 2.52 (t, *J* = 6.9 Hz, 2H), 2.10 (p, *J* = 6.9 Hz, 2H). **^{13}C{^{1}H} NMR** (151 MHz, $CDCl_3$) δ 196.9, 163.9, 130.4, 129.7, 119.7, 55.7, 36.0, 20.0, 16.8. **HRMS** (ESI-TOF) m/z calculated for $C_{12}H_{14}NO_2$ $(M+H)^+$: 204.1019 found 204.1016. The characterization data matches a previous report.[29]

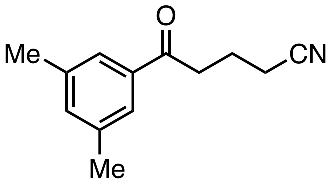

5-(3,5-dimethylphenyl)-5-oxopentanenitrile (**3h**)

Following general procedure A, **1h** (0.15 mmol, 26.1 mg) was employed. The reaction was heated to 110 °C for 6 h. The resulting residue was purified by flash chromatography (silica, 10% EtOAc in hexanes, **R*f*** = 0.10 in 10% EtOAc in hexanes) to afford 21.2 mg (70% yield) of **3h** as a yellow solid (**mp** = 35.2-36.6 °C). **^{1}H NMR** (600 MHz, $CDCl_3$) δ 7.57 (s, 2H), 7.22 (s, 1H), 3.15 (t, *J* = 6.9 Hz, 2H), 2.52 (t, *J* = 6.9 Hz, 2H), 2.38 (s, 6H), 2.10 (p, *J* = 6.9 Hz, 2H). **^{13}C{^{1}H} NMR** (151 MHz, $CDCl_3$) δ 198.7, 138.5, 136.7, 135.2, 125.9, 119.6, 36.5, 21.4, 19.9, 16.8. **HRMS** (ESI-TOF) m/z calculated for $C_{13}H_{15}NO$ $(M+H)^+$: 202.1229 found 202.1224. The characterization data matches a previous report.[29]

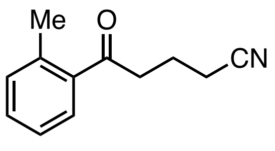

5-oxo-5-(*o*-tolyl)pentanenitrile (**3i**)

Following general procedure A, **1i** (0.15 mmol, 24.0 mg) was employed. The reaction was heated to 110 °C for 6 h. The resulting residue was purified by flash chromatography (silica, 5-10% gradient EtOAc in hexanes, **R*f*** = 0.14 in 10% EtOAc in hexanes) to afford 15.0 mg (53% yield) of **3i** as a yellow oil. **^{1}H NMR** (400 MHz, $CDCl_3$) δ 7.69 (d, *J* = 7.8 Hz, 1H), 7.44 – 7.32 (m, 1H), 7.31 – 7.23 (m, 2H), 3.11 (t, *J* = 6.8 Hz, 2H), 2.57 – 2.48 (m, 5H), 2.09 (p, *J* = 6.9 Hz, 2H). **^{13}C{^{1}H} NMR** (101 MHz, $CDCl_3$) δ 202.0, 138.5, 137.2, 132.3, 131.9, 128.7, 126.0, 119.5, 39.1, 21.6, 20.0, 16.8. **HRMS** (ESI-TOF) m/z calculated for $C_{12}H_{13}NO$ $(M+H)^+$: 188.1070 found 188.1061. The characterization data matches a previous report.[29]

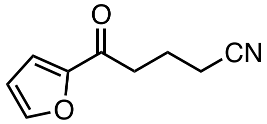

5-(furan-2-yl)-5-oxopentanenitrile (**3j**)

Following general procedure A, **1j** (0.15 mmol, 20.4 mg) was employed. The reaction was heated to 110 °C for 6 h. The resulting residue was purified by flash chromatography (silica, 0-30% gradient EtOAc in hexanes, **R*f*** = 0.23 in 30% EtOAc in hexanes) to afford 15.8 (65% yield) of **3j** as a yellow oil. **^{1}H NMR** (600 MHz, C_6D_6) δ 6.83 – 6.80 (m, 1H), 6.76 (d, *J* = 3.5 Hz, 1H), 5.84 (dd, *J* = 3.5, 1.7 Hz, 1H), 2.33 (t, *J* = 7.0 Hz, 2H), 1.49 (t, *J* = 7.0 Hz, 2H), 1.39 (p, *J* = 7.0 Hz, 2H). **^{13}C{^{1}H} NMR** (151 MHz, C_6D_6) δ 186.6, 153.0, 145.9, 119.1, 116.4, 112.2, 36.2, 19.5, 16.0. **HRMS** (ESI-TOF) m/z calculated for $C_9H_9NO_2$ $(M+H)^+$: 164.0706 found 164.0703. The characterization data matches a previous report.[32]

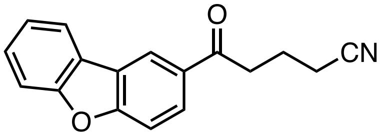

5-(dibenzo[*b*,*d*]furan-2-yl)-5-oxopentanenitrile (**3k**)

Following general procedure A, **1k** (0.15 mmol, 35.4 mg) was employed. The reaction was heated to 110 °C for 6 h. The resulting residue was purified by flash chromatography (silica, 0-20% gradient EtOAc in hexanes, **R*f*** = 0.28 in 20% EtOAc in hexanes) to afford 28.0 mg (70% yield) of **3k** as a pale yellow solid (**mp** = 122.1-124.7 °C). **^{1}H NMR** (400 MHz, $CDCl_3$) δ 8.60 (dd, *J* = 1.9, 0.7 Hz, 1H), 8.12 (dd, *J* = 8.6, 1.9 Hz, 1H), 8.02 (ddd, *J* = 7.7, 1.3, 0.7 Hz, 1H), 7.67 – 7.57 (m, 2H), 7.52 (ddd, *J* = 8.4, 7.3, 1.3 Hz, 1H), 7.45 – 7.37 (m, 1H), 3.31 (t, *J* = 6.9 Hz, 2H), 2.58 (t, *J* = 6.9 Hz, 2H), 2.18 (p, *J* = 6.9 Hz, 2H). **^{13}C{^{1}H} NMR** (101 MHz, $CDCl_3$) δ 197.4, 159.2, 157.0, 131.9, 128.3, 127.7, 124.9, 123.8, 123.6, 121.5, 121.1, 119.6, 112.1, 112.0, 36.6, 20.0, 16.9. **HRMS** (ESI-TOF) m/z calculated for $C_{17}H_{13}NO_2$ $(M+H)^+$: 264.1019 found 264.1022.

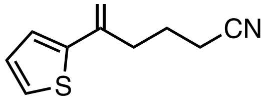

5-oxo-5-(thiophen-2-yl)pentanenitrile (**3l**)

Following general procedure A, **1l** (0.15 mmol, 22.8 mg) was employed. The reaction was heated to 110 °C for 6 h. The resulting residue was purified by flash chromatography (silica, 0-25% gradient EtOAc in hexanes, **R*f*** = 0.18 in 25% EtOAc in hexanes) to afford 17.6 mg (66% yield) of **3l** as a yellow solid (**mp** = 34.8-36.3 °C). **^{1}H NMR** (600 MHz, $CDCl_3$) δ 7.77 – 7.73 (m, 1H), 7.68 – 7.65 (m, 1H), 7.17 – 7.13 (m, 1H), 3.12 (t, *J* = 6.9 Hz, 2H), 2.52 (t, *J* = 6.9 Hz, 2H), 2.11 (p, *J* = 6.9 Hz, 2H). **^{13}C{^{1}H} NMR** (151 MHz, $CDCl_3$) δ 191.2, 143.7, 134.2, 132.3, 128.4, 119.4, 37.0, 20.0, 16.8. **HRMS** (ESI-TOF) m/z calculated for C_9H_9NOS $(M-H)^-$: 178.034 found 178.0324. The characterization data matches a previous report.[32]

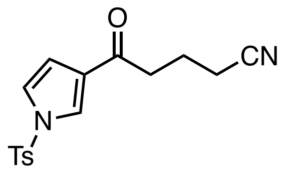

5-oxo-5-(1-tosyl-1*H*-pyrrol-3-yl)pentanenitrile (**3m**)

Following general procedure A, **1m** (0.15 mmol, 43.4 mg) was employed. The reaction was heated to 110 °C for 6 h. The resulting residue was purified by flash chromatography (silica, 10-30% gradient EtOAc in hexanes, **R*f*** = 0.18 in 30% EtOAc in hexanes) to afford 27.8 mg (59% yield) of **3m** as a brown oil. **^{1}H NMR** (400 MHz, $CDCl_3$) δ 7.84 – 7.78 (m, 2H), 7.75 (t, *J* = 2.0 Hz, 1H), 7.34 (d, *J* = 8.2 Hz, 2H), 7.14 (dd, *J* = 3.3, 2.0 Hz, 1H), 6.67 (dd, *J* = 3.3, 1.7 Hz, 1H), 2.93 (t, *J* = 6.9 Hz, 2H), 2.46 (t, *J* = 6.9 Hz, 2H), 2.42 (s, 3H), 2.03 (p, *J* = 6.9 Hz, 2H). **^{13}C{^{1}H} NMR** (101 MHz, $CDCl_3$) δ 193.3, 146.2, 135.0, 130.5, 128.6, 127.4, 124.4, 121.8, 119.5, 112.2, 37.3, 21.8, 19.6, 16.7. **HRMS** (ESI-TOF) m/z calculated for $C_{16}H_{16}N_2O_3S$ $(M+H)^+$: 317.0954 found 317.0938.

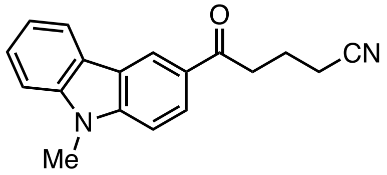

5-(9-methyl-9*H*-carbazol-3-yl)-5-oxopentanenitrile (**3n**)

Following general procedure A, **1n** (0.15 mmol, 37.4 mg) was employed. The reaction was heated to 110 °C for 6 h. The resulting residue was purified by flash chromatography (silica, 0-30% gradient EtOAc in hexanes, **R*f*** = 0.21 in 30% EtOAc in hexanes) to afford 28.0 mg (75% yield) of **3n** as an off-white solid (**mp** = 127.0-130.8 °C). **^{1}H NMR** (400 MHz, C_6D_6) δ 8.74 (s, 1H), 8.19 – 8.15 (m, 1H), 7.97 (d, *J* = 7.8 Hz, 1H), 7.37 (ddd, *J* = 8.2, 7.2, 1.2 Hz, 1H), 7.24 – 7.19 (m, 1H), 6.96 (d, *J* = 8.2 Hz, 1H), 6.88 (d, *J* = 8.6 Hz, 1H), 2.91 (s, 3H), 2.67 (t, *J* = 6.6 Hz, 2H), 1.75 – 1.58 (m, 4H). **^{13}C{^{1}H} NMR** (101 MHz, C_6D_6) δ 196.6, 143.8, 142.0, 128.9, 126.7, 126.3, 123.5, 122.9, 121.7, 120.9, 120.4, 119.4, 109.2, 108.4, 36.1, 28.4, 20.4, 16.3. **HRMS** (ESI-TOF) m/z calculated for $C_{18}H_{16}N_2O$ $(M+H)^+$: 277.1336 found 277.1333.

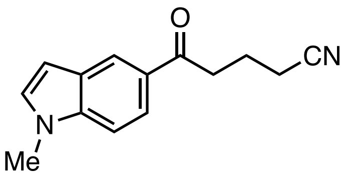

5-(1-methyl-1*H*-indol-5-yl)-5-oxopentanenitrile (**3o**)

Following general procedure A, **1o** (0.15 mmol, 29.9 mg) was employed. The reaction was heated to 110 °C for 6 h. The resulting residue was purified by flash chromatography (silica, 0-30% gradient EtOAc in hexanes, **R*f*** = 0.18 in 30% EtOAc in hexanes) to afford 12.9 mg (38% yield) of **3o** as an off-white solid (**mp** = 105.6-107.1 °C). **^{1}H NMR** (400 MHz, C_6D_6) δ 8.25 (dd, *J* = 1.7, 0.7 Hz, 1H), 8.07 (dd, *J* = 8.7, 1.7 Hz, 1H), 6.90 (d, *J* = 8.7 Hz, 1H), 6.48 (s, 2H), 2.81 (s, 3H), 2.63 (t, *J* = 6.6 Hz, 2H), 1.68 – 1.55 (m, 4H). **^{13}C{^{1}H} NMR** (101 MHz, C_6D_6) δ 197.1, 139.4, 130.2, 129.6, 123.1, 121.9, 119.4, 109.5, 103.3, 36.2, 32.1, 20.4, 16.3. One aromatic signal is missing, likely buried under $CDCl_3$ peak. **HRMS** (ESI-TOF) m/z calculated for $C_{14}H_{14}N_2O$ $(M+H)^+$: 227.1179 found 227.1180.

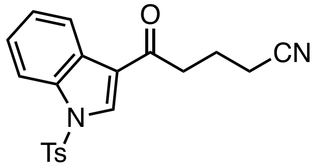

5-oxo-5-(1-tosyl-1*H*-indol-3-yl)pentanenitrile (**3p**)

Following general procedure A, **1p** (0.15 mmol, 50.9 mg) was employed. The reaction was heated to 110 °C for 6 h. The resulting residue was purified by flash chromatography (silica, 20-30% gradient EtOAc in hexanes, **R*f*** = 0.42 in 40% EtOAc in hexanes) to afford 41.5 mg (76% yield) of **3p** as a viscous brown oil. **^{1}H NMR** (600 MHz, $CDCl_3$) δ 8.29 (d, *J* = 7.9 Hz, 1H), 8.27 (s, 1H), 7.94 (d, *J* = 8.2 Hz, 1H), 7.85 (d, *J* = 8.0 Hz, 2H), 7.41 – 7.32 (m, 2H), 7.28 (d, *J* = 8.1 Hz, 2H), 3.12 (t, *J* = 6.9 Hz, 2H), 2.53 (t, *J* = 6.9 Hz, 2H), 2.36 (s, 3H), 2.13 (p, *J* = 6.9 Hz, 2H). **^{13}C{^{1}H} NMR** (151 MHz, $CDCl_3$) δ 194.0, 146.2, 134.9, 134.4, 132.0, 130.4, 127.5, 127.3, 126.0, 125.0, 123.0, 120.8, 119.6, 113.2, 37.5, 21.8, 19.8, 16.8. **HRMS** (ESI-TOF) m/z calculated for $C_{20}H_{18}N_2O_3S$ $(M+H)^+$: 367.1113 found 367.1103.

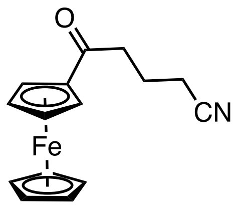

5-ferrocenyl-5-oxopentanenitrile (**3q**)

Following general procedure A, **1q** (0.15 mmol, 38.1 mg) was employed. The reaction was heated to 110 °C for 6 h. The resulting residue was purified by flash chromatography (silica, 0-20% gradient EtOAc in hexanes, **R*f*** = 0.27 in 30% EtOAc in hexanes) then recrystallized from toluene and pentane to afford 21.6 mg (51% yield) of **3q** as a crystalline red solid (**mp** = 68.5-69.4 °C). **^{1}H NMR** (600 MHz, $CDCl_3$) δ 4.80 (s, 2H), 4.54 (s, 2H), 4.22 (s, 5H), 2.93 (t, *J* = 6.8 Hz, 2H), 2.54 (t, *J* = 6.8 Hz, 2H), 2.06 (p, *J* = 6.8 Hz, 2H). **^{13}C{^{1}H} NMR** (151 MHz, $CDCl_3$) δ 202.6, 119.7, 72.7, 70.1, 69.4, 37.2, 19.9, 16.9. Ipso-ferrocene likely buried under $CDCl_3$ peak. **HRMS** (ESI-TOF) m/z calculated for $C_{15}H_{15}FeNO$ $(M+H)^+$: 282.0576 found 282.0572.

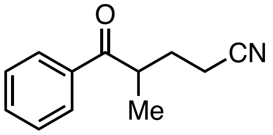

(±)-4-methyl-5-oxo-5-phenylpentanenitrile (**3r**)

Following general procedure A, **1r** (0.15 mmol, 24.0 mg) was employed. The reaction was heated to 110 °C for 6 h. The resulting residue was purified by flash chromatography (silica, 10% EtOAc in hexanes, **R*f*** = 0.13 in 10% EtOAc in hexanes) to afford 20.7 mg (79% yield) of **3r** as a yellow oil. **^{1}H NMR** (400 MHz, $CDCl_3$) δ 7.99 – 7.94 (m, 2H), 7.62 – 7.56 (m, 1H), 7.52 – 7.46 (m, 2H), 3.68 (dtd, *J* = 8.1, 7.1, 5.5 Hz, 1H), 2.49 – 2.29 (m, 2H), 2.22 (ddt, *J* = 13.6, 8.1, 6.7 Hz, 1H), 1.80 (dddd, *J* = 13.6, 8.1, 7.1, 5.5 Hz, 1H), 1.25 (d, *J* = 7.1 Hz, 3H). **^{13}C{^{1}H} NMR** (101 MHz, $CDCl_3$) δ 202.5, 135.9, 133.6, 129.0, 128.5, 119.5, 39.4, 28.4, 18.0, 15.3. **HRMS** (ESI-TOF) m/z calculated for $C_{12}H_{13}NO$ $(M+H)^+$: 188.1069 found 188.1065. The characterization data matches a previous report.[31]

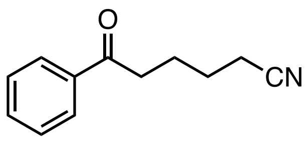

6-oxo-6-phenylhexanenitrile (**3s**)

Following general procedure A, **1s** (0.15 mmol, 24.0 mg) was employed. The reaction was heated to 110 °C for 6 h. The resulting residue was purified by flash chromatography (silica, 10-20% gradient EtOAc in hexanes, ***Rf*** = 0.22 in 20% EtOAc in hexanes) to afford 11.6 mg (41% yield) of **3s** as a pale yellow solid (**mp** = 64.5-67.0 °C). **^{1}H NMR** (600 MHz, $CDCl_3$) δ 7.97 – 7.93 (m, 2H), 7.60 – 7.54 (m, 1H), 7.47 (t, *J* = 7.7 Hz, 2H), 3.04 (t, *J* = 7.2 Hz, 2H), 2.41 (t, *J* = 7.2 Hz, 2H), 1.91 (p, *J* = 7.2 Hz, 2H), 1.76 (p, *J* = 7.2 Hz, 2H). **^{13}C{^{1}H} NMR** (151 MHz, $CDCl_3$) δ 199.2, 136.8, 133.4, 128.8, 128.1, 128.0, 119.7, 37.5, 25.1, 23.2, 17.3. **HRMS** (ESI-TOF) m/z calculated for $C_{12}H_{13}NO$ $(M+H)^+$: 188.1068 found 188.1065. The characterization data matches a previous report.[29]

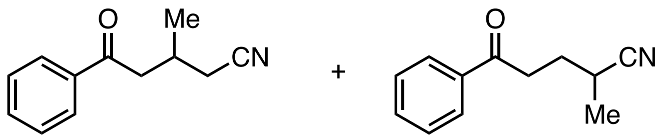

(±)-3-methyl-5-oxo-5-phenylpentanenitrile (**3t**)

&

(±)-2-methyl-5-oxo-5-phenylpentanenitrile (**3t')**

Following general procedure A, **1t** (0.15 mmol, 24.0 mg) was employed. The reaction was heated to 110 °C for 6 h. The resulting residue was purified by flash chromatography (silica, 10% EtOAc in hexanes, **R*f*** = 0.14 in 10% EtOAc in hexanes) to afford 19.9 mg (71% yield, 57:43 r.r.) of **3t** and **3t'** as a colorless oil (71% overall yield). **HRMS** (ESI-TOF) m/z calculated for $C_{12}H_{13}NO$ $(M+H)^+$: 188.1070 found 188.1071. The characterization data matches a previous report.[33,34]

(3t) ^{1}**H NMR** (400 MHz, $CDCl_3$) δ 8.01 – 7.93 (m, 2H), 7.64 – 7.54 (m, 1H), 7.51 – 7.46 (m, 2H), 3.12 (dd, *J* = 17.6, 7.3 Hz, 1H), 3.02 (dd, *J* = 17.6, 5.8 Hz, 1H), 2.69 – 2.55 (m, 1H), 2.52 (d, *J* = 5.8 Hz, 2H), 1.20 (d, *J* = 6.7 Hz, 3H). ^{13}**C{^{1}H} NMR** (101 MHz, $CDCl_3$) δ 198.2, 136.9, 133.62, 128.9, 128.1, 118.5, 43.7, 26.4, 24.2, 19.9.

(**3t'**) ^{1}**H NMR** (400 MHz, $CDCl_3$) δ 8.02 – 7.92 (m, 2H), 7.63 – 7.54 (m, 1H), 7.52 – 7.44 (m, 2H), 3.27 – 3.16 (m, 2H), 2.90 – 2.76 (m, 1H), 2.20 – 2.05 (m, 1H), 2.04 – 1.89 (m, 1H), 1.40 (d, *J* = 7.0 Hz, 3H). ^{13}**C{^{1}H} NMR** (101 MHz, $CDCl_3$) δ 198.5, 136.6, 133.6, 128.9, 128.1, 122.8, 35.6, 28.3, 25.2, 18.3.

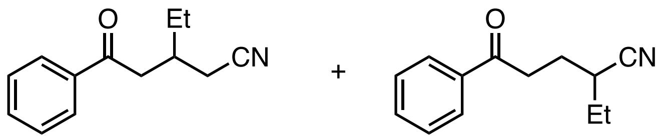

(±)-3-ethyl-5-oxo-5-phenylpentanenitrile (**3u**)

&

(±)-2-ethyl-5-oxo-5-phenylpentanenitrile (**3u'**)

Following general procedure A, **1u** (0.15 mmol, 26.1 mg) was employed. The reaction was heated to 110 °C for 6 h. The resulting residue was purified by flash chromatography (silica, 0-10% gradient EtOAc in hexanes, **R*f*** = 0.26 in 10% EtOAc in hexanes) to afford 21.3 mg (78% yield, 57:43 r.r.) of **3u** and **3u'** as a yellow oil. **HRMS** (ESI-TOF) m/z calculated for $C_{13}H_{15}NO$ $(M+H)^+$: 202.1226 found 202.1228.

(**3u**) **^{1}H NMR** (400 MHz, $CDCl_3$) δ 8.01 – 7.93 (m, 2H), 7.62 – 7.56 (m, 1H), 7.52 – 7.44 (m, 2H), 3.09 (d, *J* = 6.6 Hz, 2H), 2.56 (dd, *J* = 5.3, 2.1 Hz, 2H), 2.46 – 2.34 (m, 1H), 1.59 (p, *J* = 7.3 Hz, 2H), 0.99 (t, *J* = 7.3 Hz, 3H). **^{13}C{^{1}H} NMR** (101 MHz, $CDCl_3$) δ 198.5, 136.8, 133.6, 128.9, 128.1, 118.5, 41.5, 32.5, 26.6, 21.4, 11.4.

(**3u'**) **^{1}H NMR** (400 MHz, $CDCl_3$) 8.01 – 7.93 (m, 2H), 7.62 – 7.56 (m, 1H), 7.52 – 7.44 (m, 2H), 3.29 – 3.14 (m, 2H), 2.74 – 2.63 (m, 1H), 2.16 – 2.07 (m, 1H), 2.02 – 1.88 (m, 1H), 1.71 (pd, *J* = 7.5, 1.5 Hz, 2H), 1.12 (t, *J* = 7.5 Hz, 3H). **^{13}C{^{1}H} NMR** (101 MHz, $CDCl_3$) δ 198.6, 136.6, 133.6, 128.9, 128.1, 122.0, 35.7, 32.9, 26.2, 25.9, 11.7.

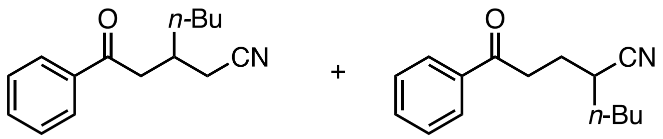

(±)-3-(2-oxo-2-phenylethyl)heptanenitrile (**3v**)

&

(±)-2-(3-oxo-3-phenylpropyl)hexanenitrile (**3v'**)

Following general procedure A, **1v** (0.15 mmol, 30.3 mg) was employed. The reaction was heated to 110 °C for 6 h. The resulting residue was purified by flash chromatography (silica, 0-5% gradient EtOAc in hexanes, **R*f*** = 0.13 in 5% EtOAc in hexanes) to afford 22.3 mg (71% yield, 62:38 r.r.) of **3v** and **3v'** as a yellow oil. **HRMS** (ESI-TOF) m/z calculated for $C_{15}H_{19}NO$ $(M+H)^+$: 230.1539 found 230.1526.

(**3v**) **^{1}H NMR** (600 MHz, $CDCl_3$) δ 8.01 – 7.93 (m, 2H), 7.61 – 7.56 (m, 1H), 7.51 – 7.45 (m, 2H), 3.15 – 3.06 (m, 2H), 2.58 (dd, *J* = 17.0, 5.4 Hz, 1H), 2.53 (dd, *J* = 17.0, 5.0 Hz, 1H), 2.50 – 2.42 (m, 1H), 1.58 – 1.50 (m, 2H), 1.40 – 1.31 (m, 4H), 0.97 – 0.88 (m, 3H). **^{13}C{^{1}H} NMR** (151 MHz, $CDCl_3$) δ 198.5, 136.8, 133.58, 128.8, 128.1, 118.6, 41.8, 33.4, 30.8, 29.1, 22.7, 21.8, 14.1.

(**3v'**) **^{1}H NMR** (600 MHz, $CDCl_3$) δ 8.01 – 7.93 (m, 2H), 7.61 – 7.56 (m, 1H), 7.51 – 7.45 (m, 2H), 3.28 – 3.15 (m, 2H), 2.72 (tt, *J* = 10.0, 5.3 Hz, 1H), 2.13 (dtd, *J* = 14.8, 7.6, 4.7 Hz, 1H), 1.99 – 1.89 (m, 1H), 1.72 – 1.60 (m, 2H), 1.50 – 1.41 (m, 1H), 1.48 – 1.36 (m, 3H), 0.97 – 0.88 (m, 3H). **^{13}C{^{1}H} NMR** (151 MHz, $CDCl_3$) δ 198.6, 136.6, 133.55, 128.8, 122.2, 122.1, 35.7, 32.3, 31.2, 29.4, 26.6, 22.3, 13.9.

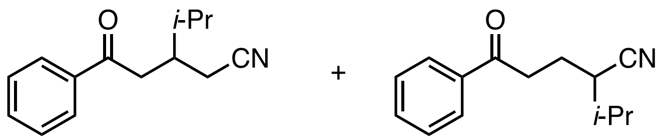

(±)-3-isopropyl-5-oxo-5-phenylpentanenitrile (**3w**)

&

(±)-2-isopropyl-5-oxo-5-phenylpentanenitrile (**3w'**)

Following general procedure A, **1w** (0.15 mmol, 28.2 mg) was employed. The reaction was heated to 110 °C for 6 h. The resulting residue was purified by flash chromatography (silica, 0-10% gradient EtOAc in hexanes, ***Rf*** = 0.24 in 10% EtOAc in hexanes) to afford 19.6 mg (70% yield, 68:32 r.r.) of **3w** and **3w'** as a yellow oil. **HRMS** (ESI-TOF) m/z calculated for $C_{14}H_{17}NO$ $(M+H)^+$: 216.1383 found 216.1382.

(**3w**) **^{1}H NMR** (400 MHz, $CDCl_3$) δ 8.00 – 7.95 (m, 2H), 7.62 – 7.56 (m, 1H), 7.52 – 7.45 (m, 2H), 3.23 – 3.14 (m, 1H), 3.07 (dd, *J* = 17.7, 8.6 Hz, 1H), 2.61 – 2.49 (m, 2H), 2.42 – 2.30 (m, 1H), 2.05 – 1.89 (m, 1H), 1.02 (t, *J* = 6.8 Hz, 6H). **^{13}C{^{1}H} NMR** (101 MHz, $CDCl_3$) δ 198.7, 136.8, 133.57, 128.9, 128.1, 119.0, 39.1, 36.8, 30.2, 19.8, 19.5.

(**3w'**) **^{1}H NMR** (400 MHz, $CDCl_3$) δ 8.00 – 7.95 (m, 2H), 7.62 – 7.56 (m, 1H), 7.52 – 7.45 (m, 2H), 3.31 – 3.14 (m, 2H), 2.65 – 2.58 (m, 1H), 2.16 – 2.06 (m, 1H), 2.02 – 1.86 (m, 2H), 1.10 (dd, *J* = 6.8, 3.5 Hz, 6H). **^{13}C{^{1}H} NMR** (101 MHz, $CDCl_3$) δ 198.6, 136.6, 133.54, 128.9, 128.1, 121.1, 38.7, 36.0, 30.5, 24.4, 21.0, 18.9.

11. References

[1] Yuan, B.; Ding, D.; Wang, C. *ACS Catal.* **2022**, *12*, 4261–4267.

[2] Cui, N.; Lin, T.; Wang, Y.-E.; Wu, J.; Han, Y.; Xu, X.; Xue, F.; Xiong, D.; Walsh, P. J.; Mao, J. *Org. Lett.* **2022**, *24*, 3987–3992.

[3] Ankade, S. B.; Samal, P. P.; Soni, V.; Gonnade, R. G.; Krishnamurty, S.; Punji, B. *ACS Catal.* **2021**, *11*, 12384–12393.

[4] Yang, S.; Wu, J.-Y.; Lin, S.; Pu, M.; Huang, Z.-S.; Wang, H.; Li, Q. *Chem. Asian J.* **2023**, *18*, e202300476.

[5] Stilz, H. U.; Guba, W.; Jablonka, B.; Just, M.; Klingler, O.; König, W.; Wehner, V.; Zoller, G. *J. Med. Chem.* **2001**, *44*, 1158–1176.

[6] Benoit, E.; Dansereau, J.; Gagnon, A. *Synlett* **2017**, *28*, 2833–2838.

[7] Sun, Y.; Huang, X.; Li, X.; Luo, F.; Zhang, L.; Chen, M.; Zheng, S.; Peng, B. *Adv. Synth. Catal.* **2018**, *360*, 1082–1087.

[8] He, Z.; Wang, Z.; Gao, Z.; Qian, H.; Ding, W.; Jin, H.; Liu, Y.; Zhou, B. *Org. Biomol. Chem.* **2023**, *21*, 6493–6497.

[9] Beutner, G. L.; Albrecht, J.; Fan, J.; Fanfair, D.; Lawler, M. J.; Bultman, M.; Chen, K.; Ivy, S.; Schild, R. L.; Tripp, J. C.; Murugesan, S.; Dambalas, K.; McLeod, D. D.; Sweeney, J. T.; Eastgate, M. D.; Conlon, D. A. *Org. Process Res. Dev.* **2017**, *21*, 1122–1130.

[10] Feng, L.; Chen, X.; Sheng, G.; Li, Y.; Li, Y.; Zhang, Y.; Yao, K.; Wu, Z.; Zhang, R.; Kiboku, T.; Kawasaki, A.; Horimoto, K.; Tang, Y.; Sun, M.; Han, F.; Chen, D. *J. Med. Chem.* **2023**, *66*, 14609–14622.

[11] Wang, J.-B.; Li, Y.-L.; Deng, J. *Adv. Synth. Catal.* **2017**, *359*, 3460–3467.

[12] Gilbert, M. M.; Trenerry, M. J.; Longley, V. R.; Castro, A. J.; Berry, J. F.; Weix, D. J. *ACS Catal.* **2023**, *13*, 11277–11290.

[13] Huo, H.; Shen, X.; Wang, C.; Zhang, L.; Röse, P.; Chen, L.-A.; Harms, K.; Marsch, M.; Hilt, G.; Meggers, E. *Nature* **2014**, *515*, 100–103.

[14] Shintani, R.; Nakatsu, H.; Takatsu, K.; Hayashi, T. *Chem. Eur. J.* **2009**, *15*, 8692–8694.

[15] Qi, X.; Yuan, F.; Yan, X.; Xia, Y. *Org. Lett.* **2024**, *26*, 10317–10321.

[16] Xu, J.; Samsuri, N. B.; Duong, H. A. *Chem. Commun.* **2016**, *52*, 3372–3375

[17] Nowrouzi, F.; Janetzko, J.; Batey, R. A. *Org. Lett.* **2010**, *12*, 5490–5493.

[18] Sani-Souna-Sido, A.; Chassaing, S.; Pale, P.; Sommer, J. *Appl. Catal. A Gen.* **2008**, *336*, 101–108.

[19] Frigerio, M.; Santagostino, M.; Sputore, S. *J. Org. Chem.* **1999**, *64*, 4537–4538.

[20] Ghosh, A.; Lipisa, Y. B.; Fridman, N.; Szpilman, A. M. *J. Org. Chem.* **2023**, *88*, 1977–1987.

[21] Tanaka, S.; Kunisawa, T.; Yoshii, Y.; Hattori, T. *Org. Lett.* **2019**, *21*, 8509–8513.

[22] Chen, H.; Deng, M.-Z. *Org. Lett.* **2000**, *2*, 1649–1651.

[23] SAINT, V8.30A, Bruker Analytical X-Ray Systems, Madison, WI, 2012.

[24] SADABS, 2.03, Bruker Analytical X-Ray Systems, Madison, WI, 2016.

[25] a) Sheldrick, G. M. *Acta Cryst*. **2008**, *A64*, 112. b) Sheldrick, G. M. *Acta Cryst*. **2015**, *A71*, 3.

[26] Dolomanov, O. V.; Bourhis, L. J.; Gildea, R. J.; Howard, J. A. K.; Puschmann, H. *J. Appl. Crystallogr.* **2009**, *42*, 339–341.
[27] P. van der Sluis, A. L. Spek, *Acta Cryst*. **1990**, *A46*, 194–201.
[28] Burés, J. *Angew. Chem. Int. Ed.* **2016**, *55,* 2028–2031.
[29] Qi, L.; Li, R.; Yao, X.; Zhen, Q.; Ye, P.; Shao, Y.; Chen, J. *J. Org. Chem.* **2020**, 85, 1097–1108.
[30] Mai, W.-P.; Liu, Y.; Sui, H.-D.; Xiao, Y.-M.; Mao, P.; Lu, K. *Eur. J. Org. Chem.* **2019**, *2019*, 7814–7819.
[31] Zhao, B.; Tan, H.; Chen, C.; Jiao, N.; Shi, Z. *Chin. J. Chem.* **2018**, *36*, 995–999.
[32] Ren, R.; Wu, Z.; Xu, Y.; Zhu, *Angew. Chem. Int. Ed.* **2016**, *55*, 2866–2869.
[33] Yasuda, M.; Ohigashi, N.; Baba, A. *Chem. Lett*., **2000**, *11*, 1266-1267.
[34] Gaspar, B.; Carreira, E. M. *Angew. Chem. Int. Ed.* **2007**, *46*, 4519–4522.

12. ^{1}H, ^{13}C, ^{19}F, and ^{31}P NMR spectra

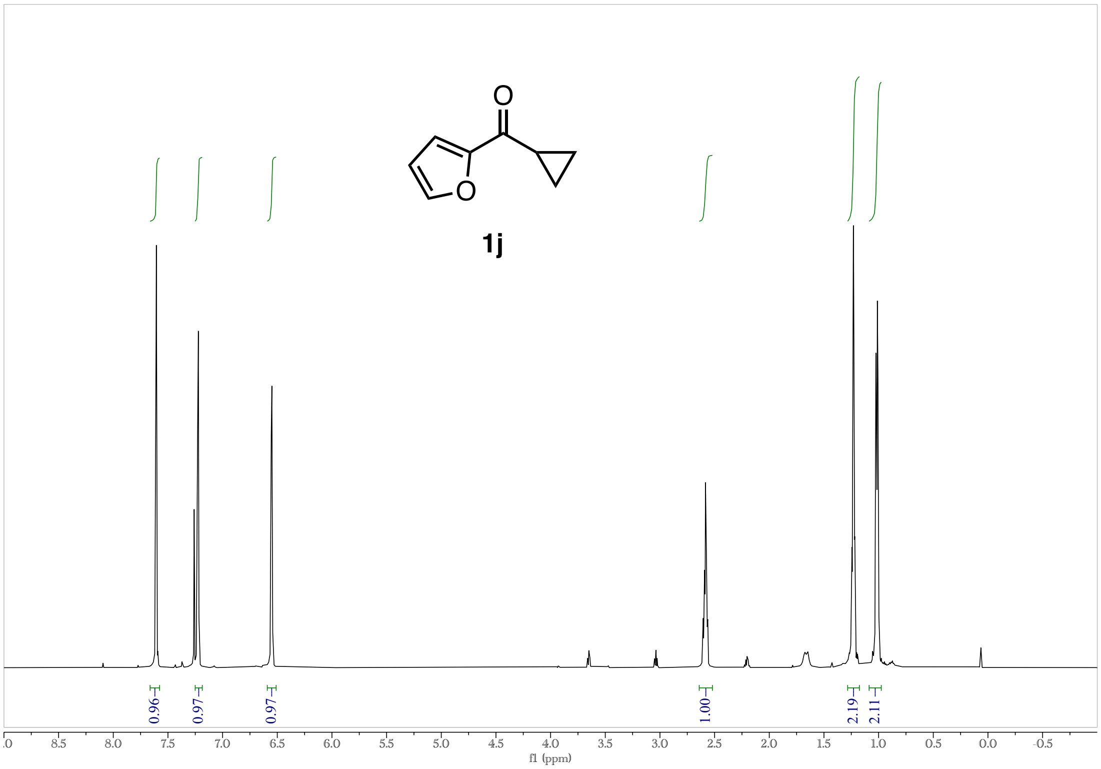

^{1}**H NMR** (600 MHz, $CDCl_3$)

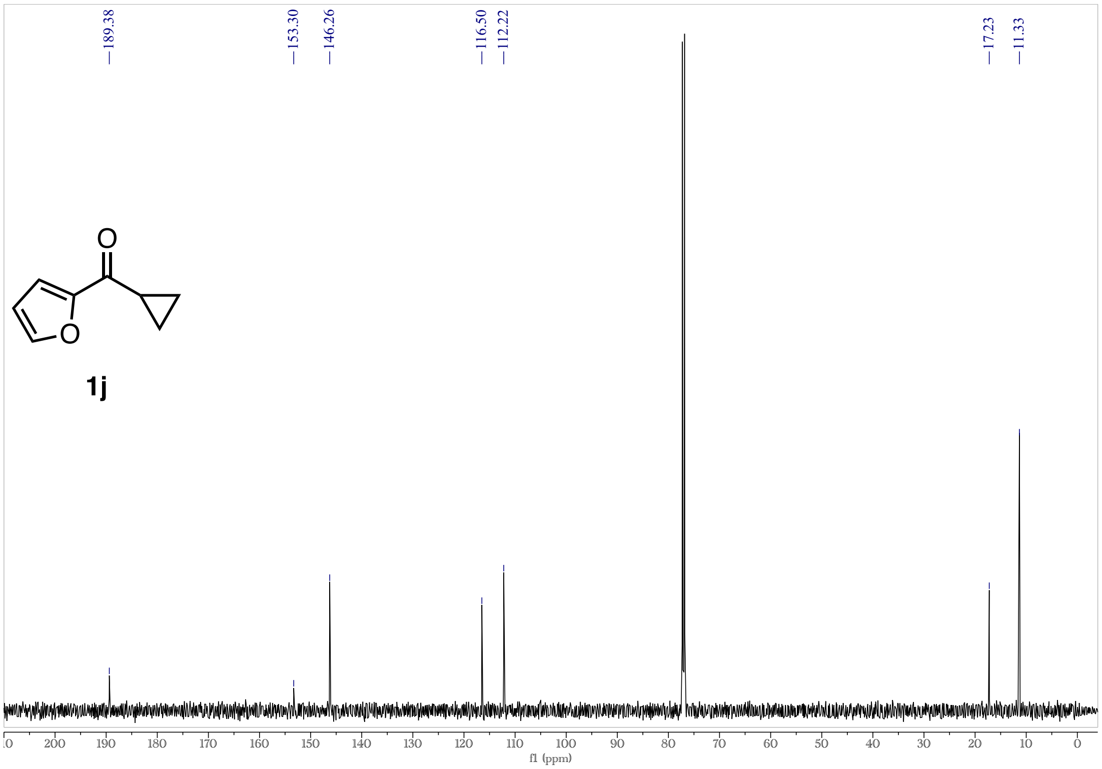

^{13}**C{**^{1}**H} NMR** (151 MHz, $CDCl_3$)

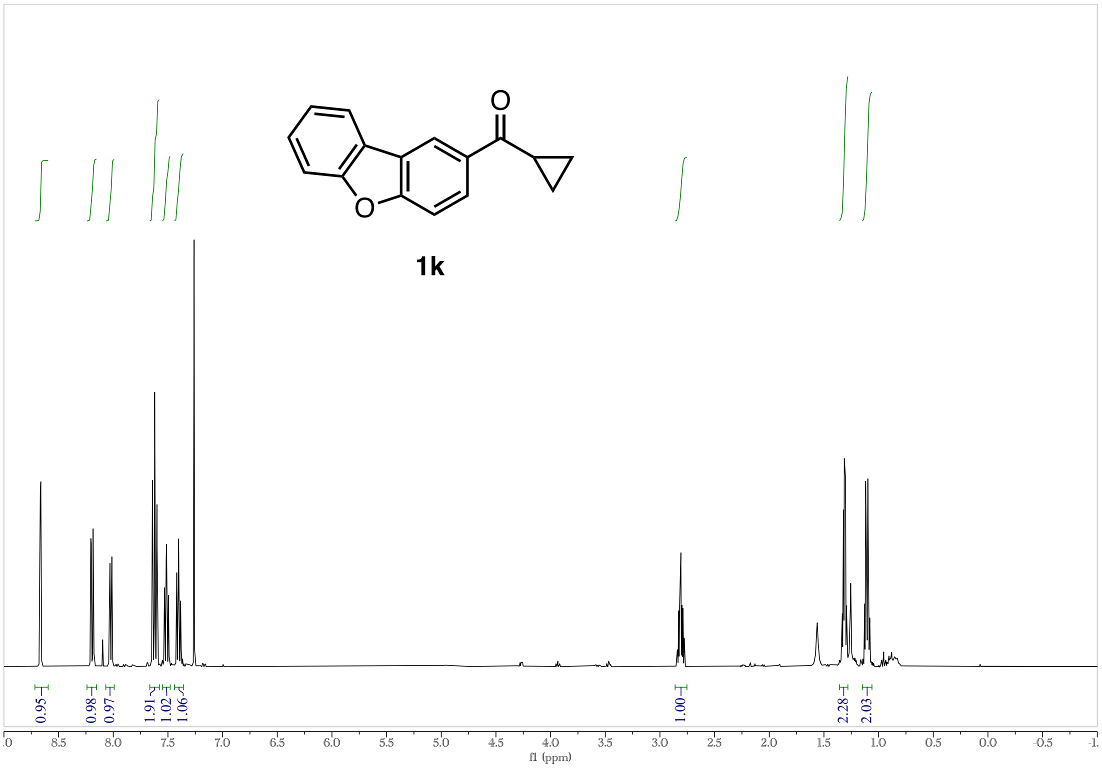

^{1}H NMR (400 MHz, $CDCl_3$)

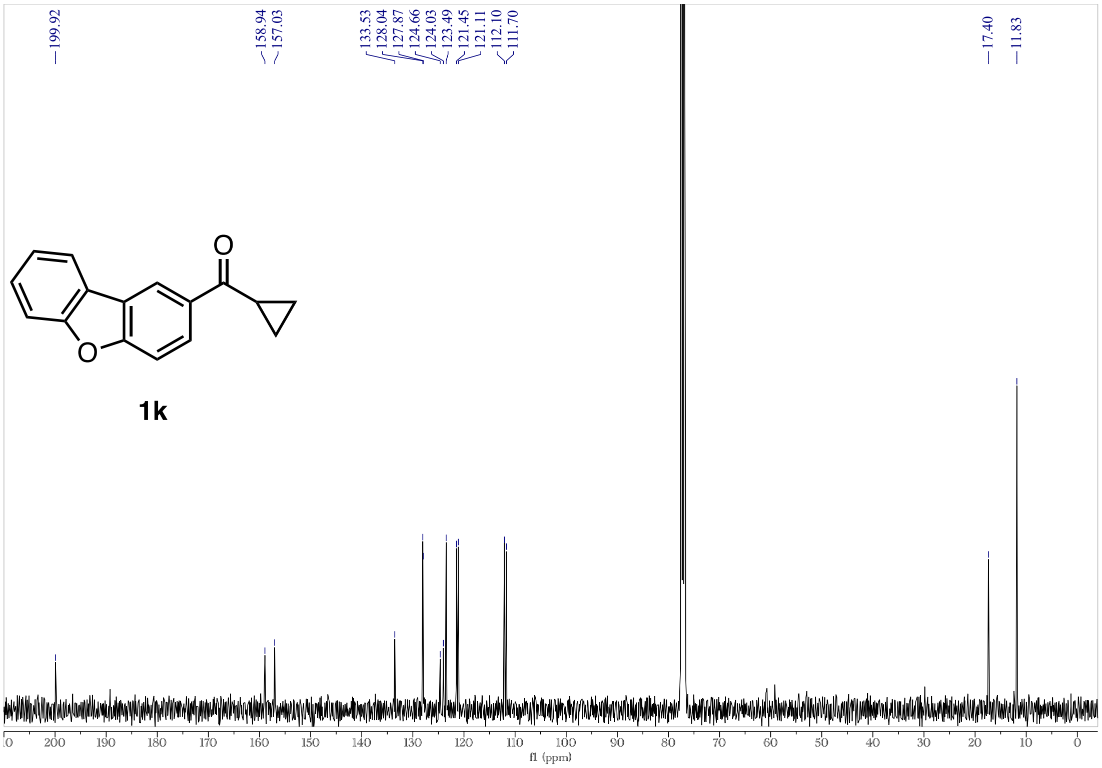

^{13}C{^{1}H} NMR (101 MHz, $CDCl_3$)

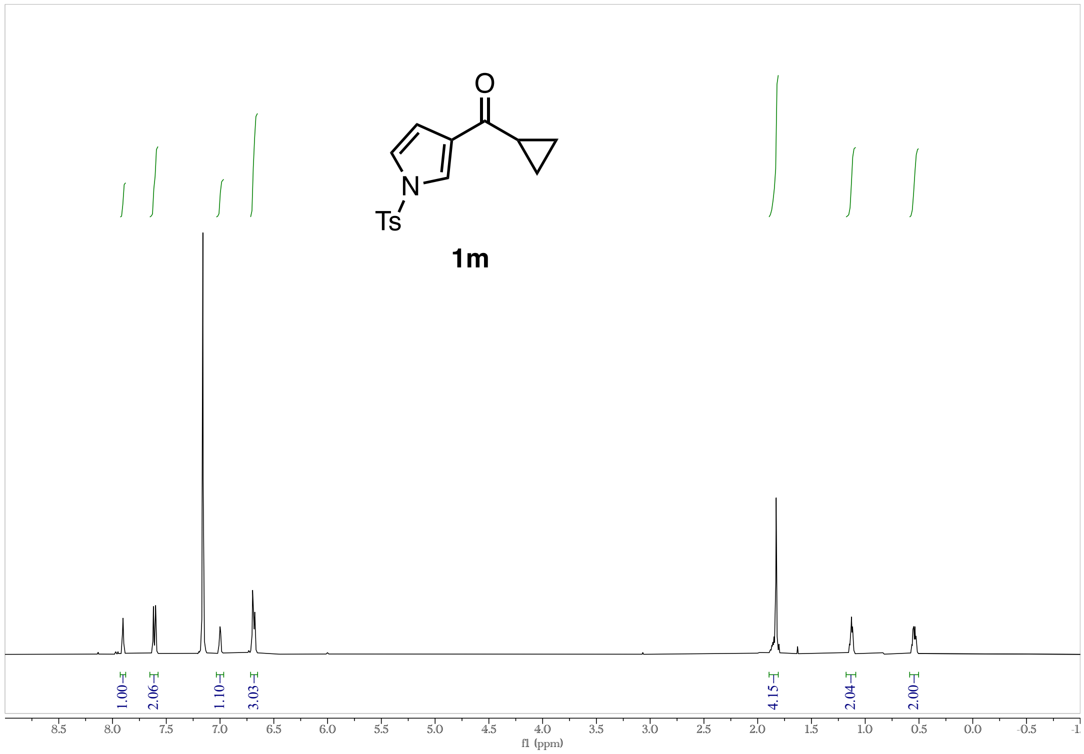

1H NMR (400 MHz, C_6D_6)

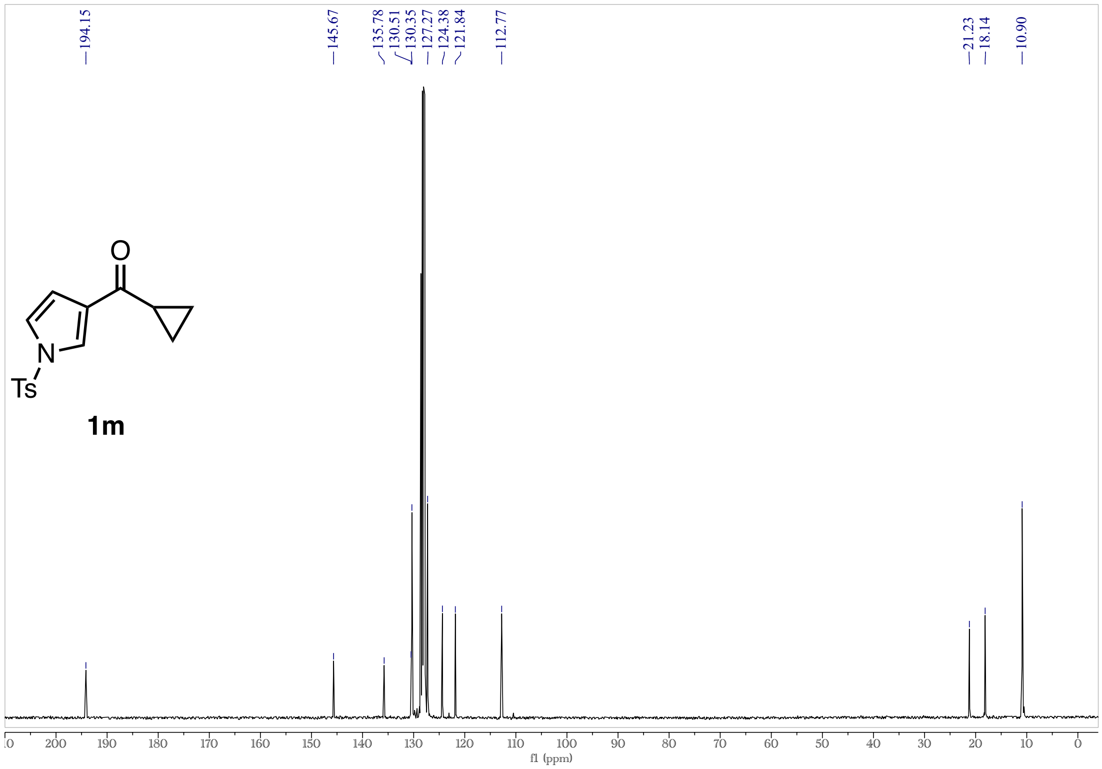

^{13}C{^{1}H} NMR (101 MHz, C_6D_6)

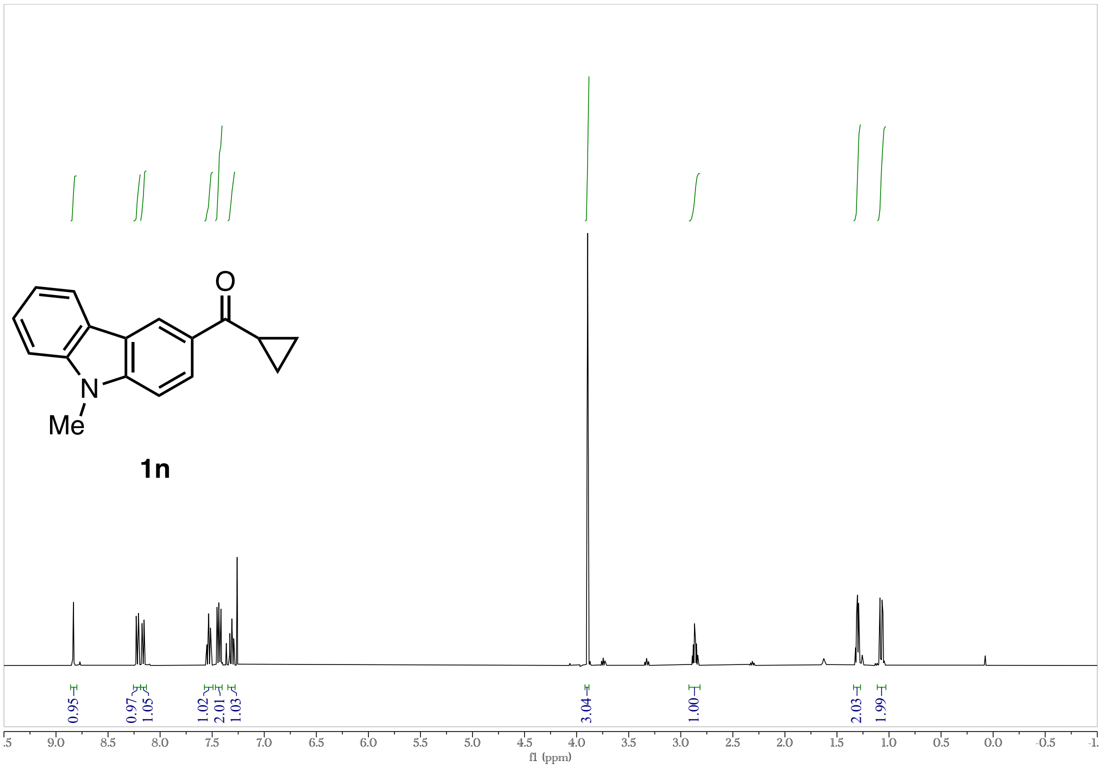

^{1}H NMR (400 MHz, $CDCl_3$)

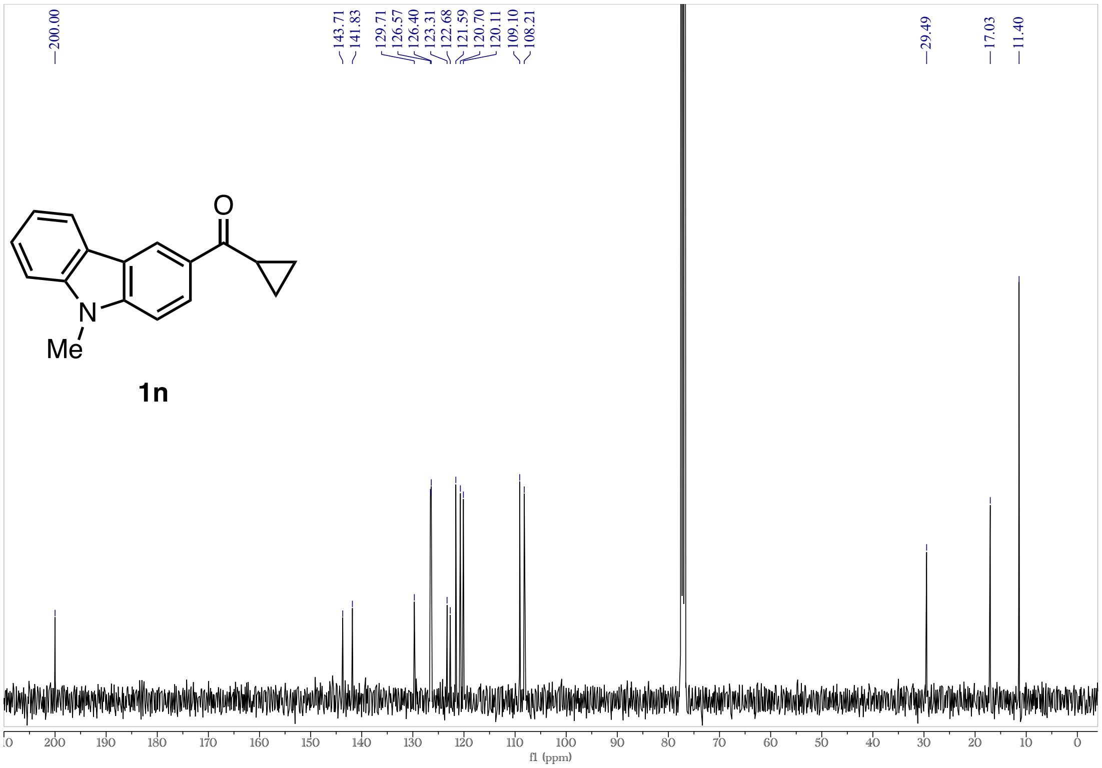

^{13}C{^{1}H} NMR (101 MHz, $CDCl_3$)

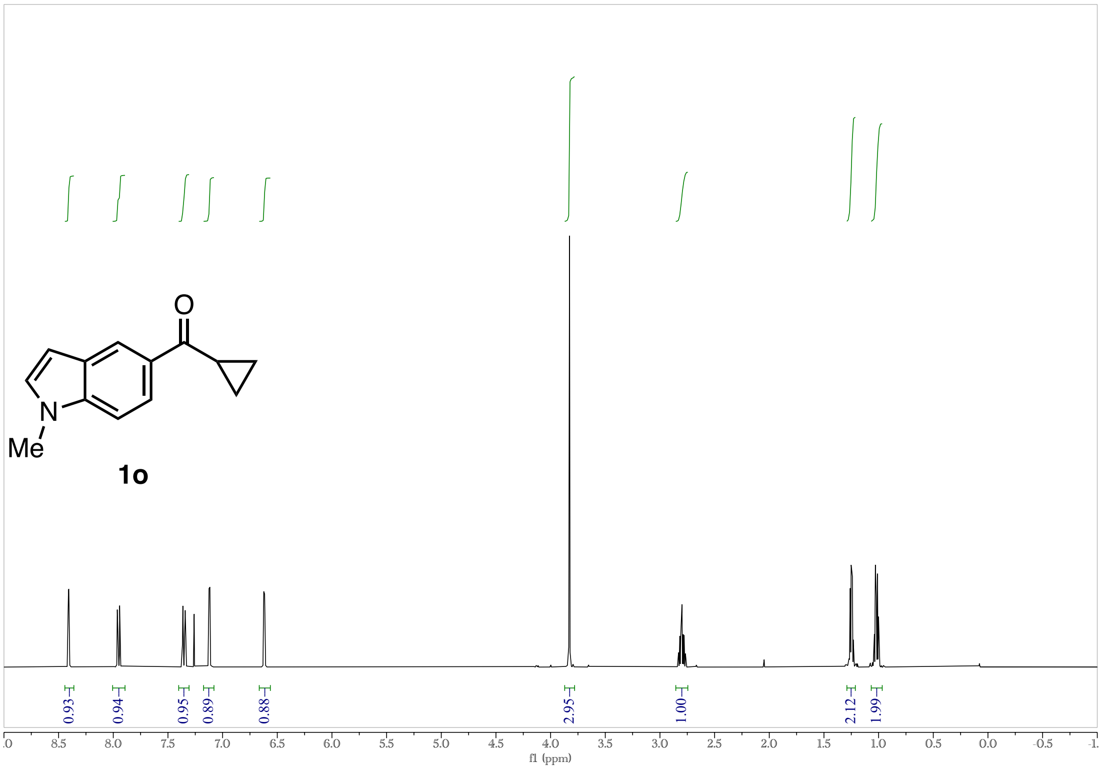

^{1}H NMR (400 MHz, $CDCl_3$)

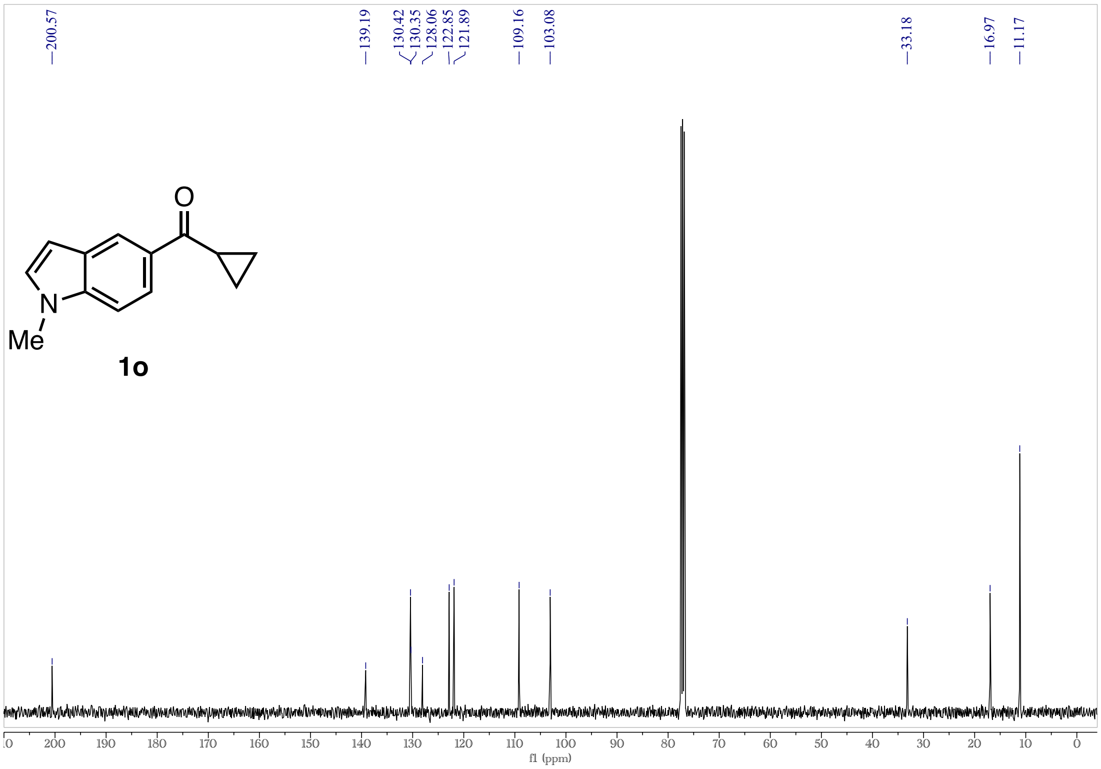

$^{13}C\{^{1}H\}$ NMR (101 MHz, $CDCl_3$)

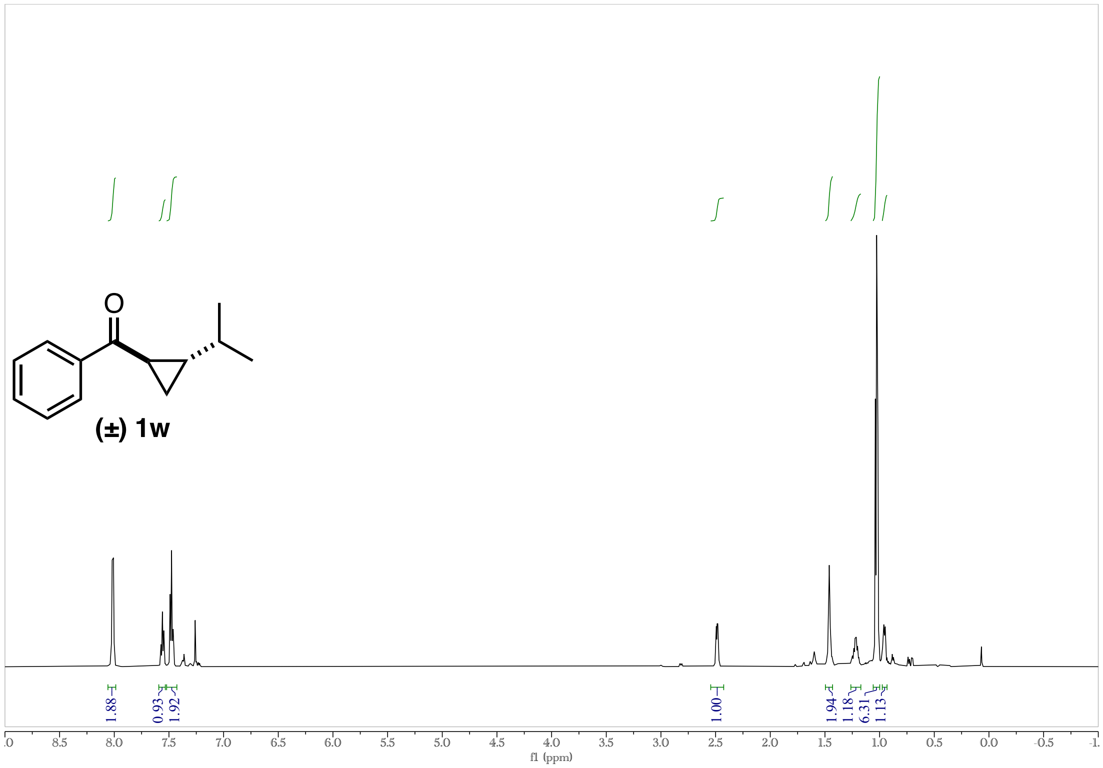

^{1}H NMR (600 MHz, $CDCl_3$)

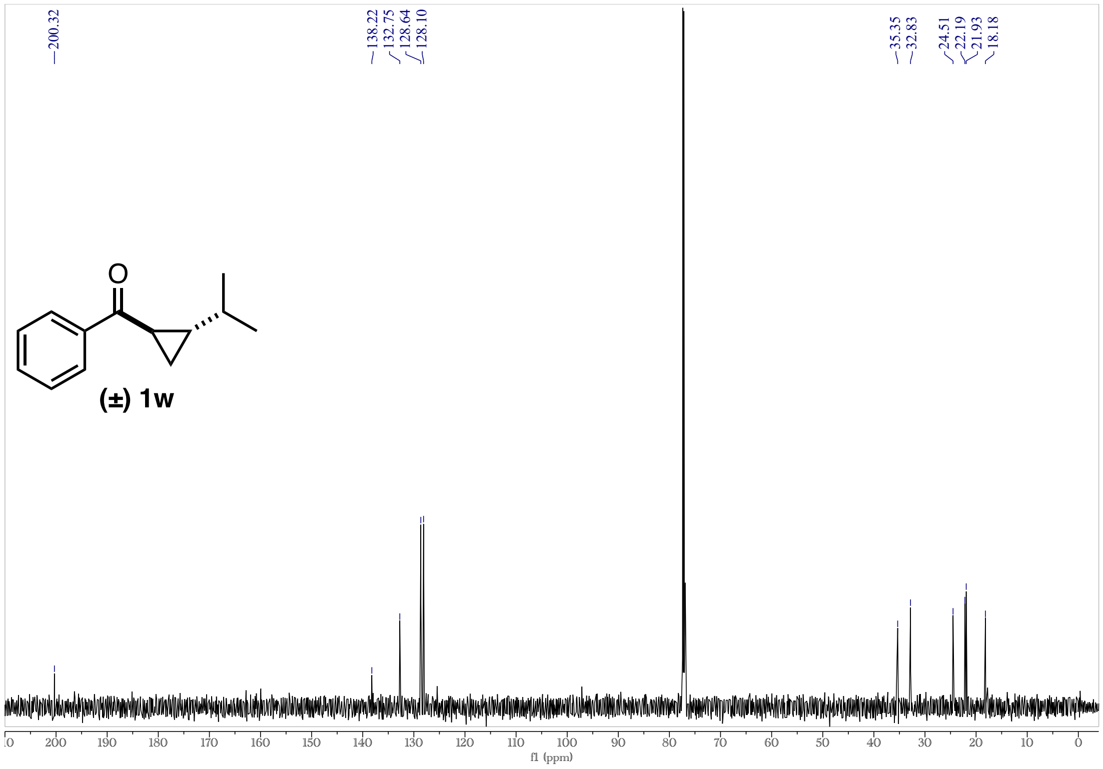

^{13}C{^{1}H} NMR (151 MHz, $CDCl_3$)

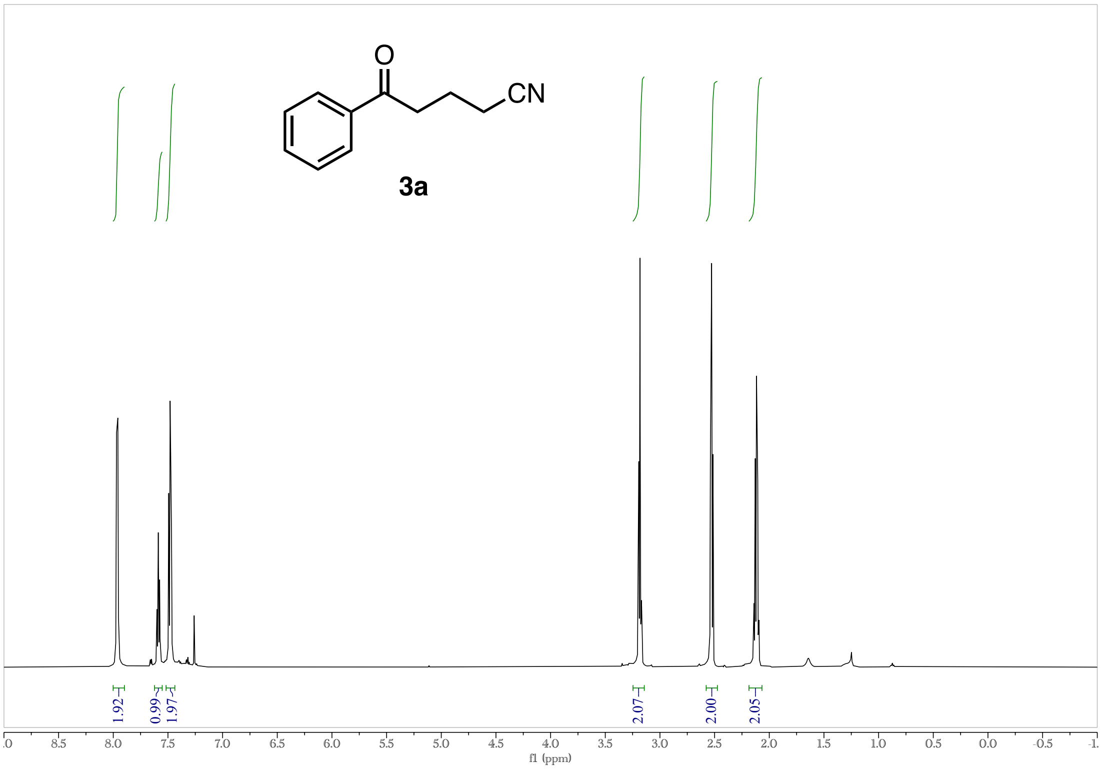

^{1}H NMR (600 MHz, $CDCl_3$)

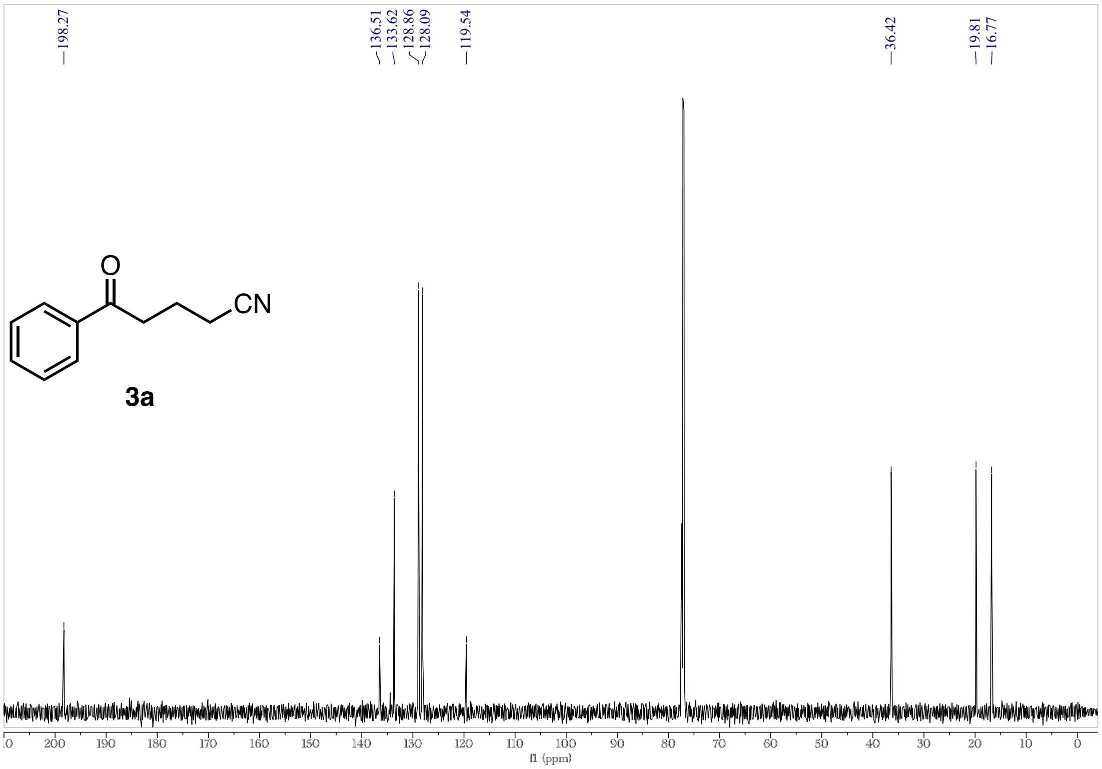

^{13}C{^{1}H} NMR (151 MHz, $CDCl_3$)

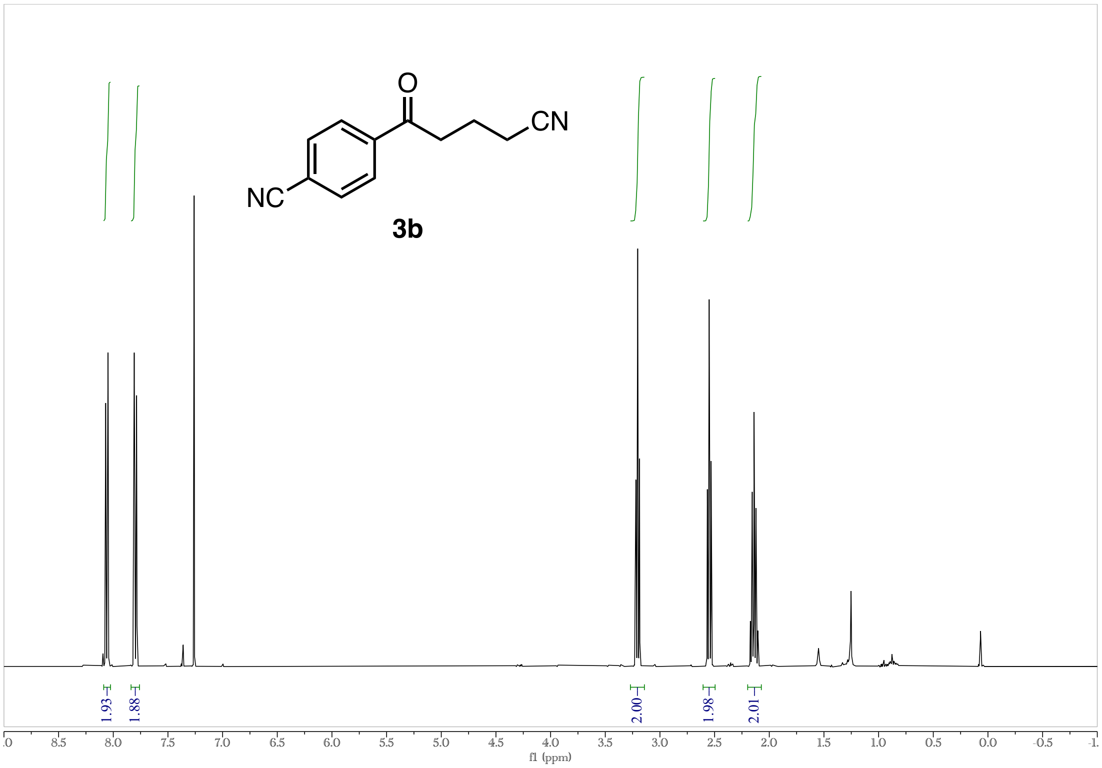

^{1}H NMR (400 MHz, $CDCl_3$)

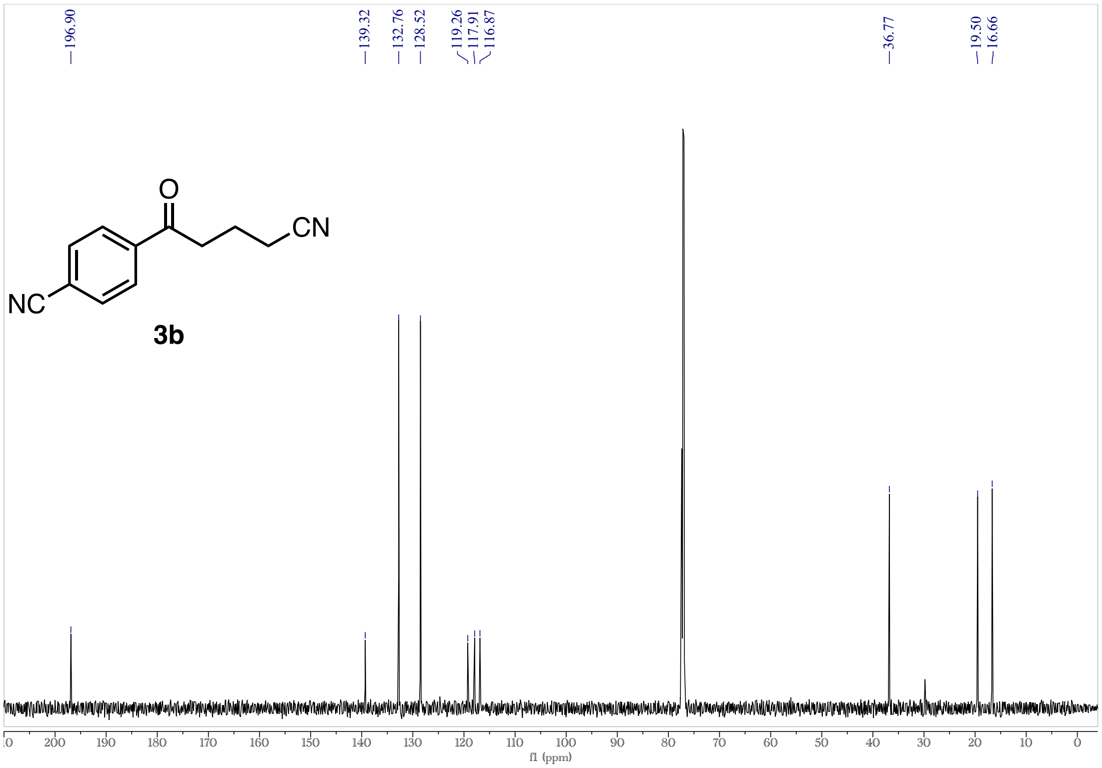

^{13}C{^{1}H} NMR (151 MHz, $CDCl_3$)

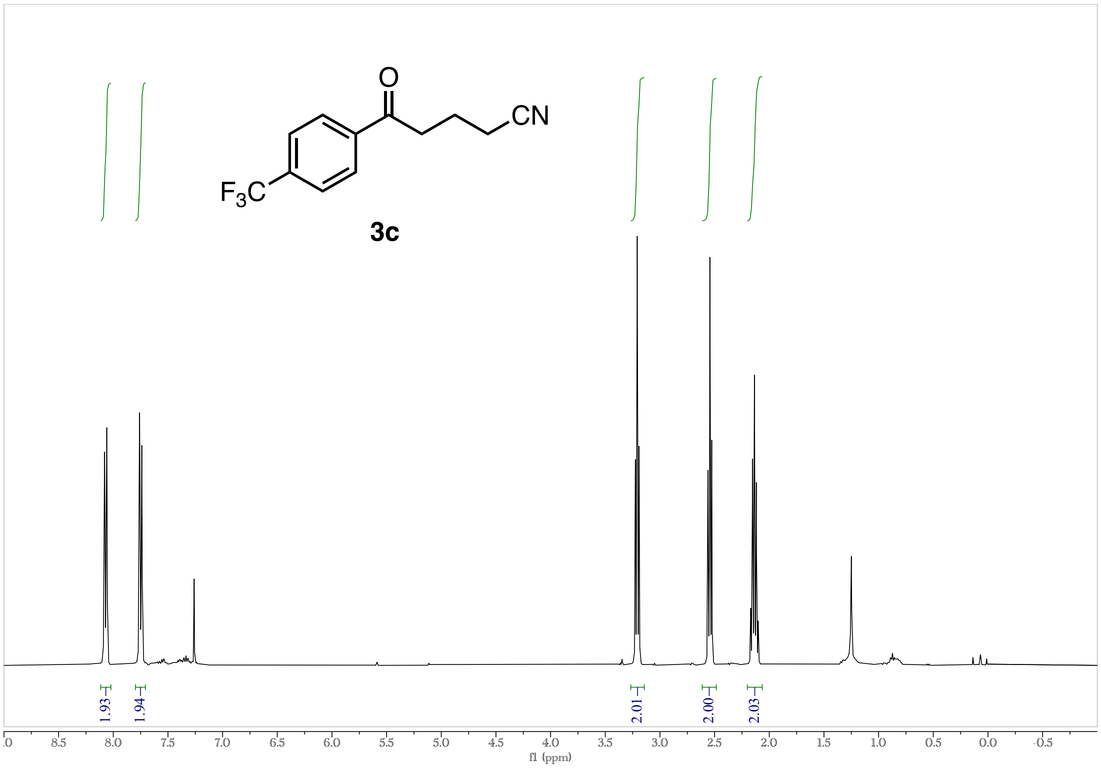

^{1}**H NMR** (400 MHz, $CDCl_3$)

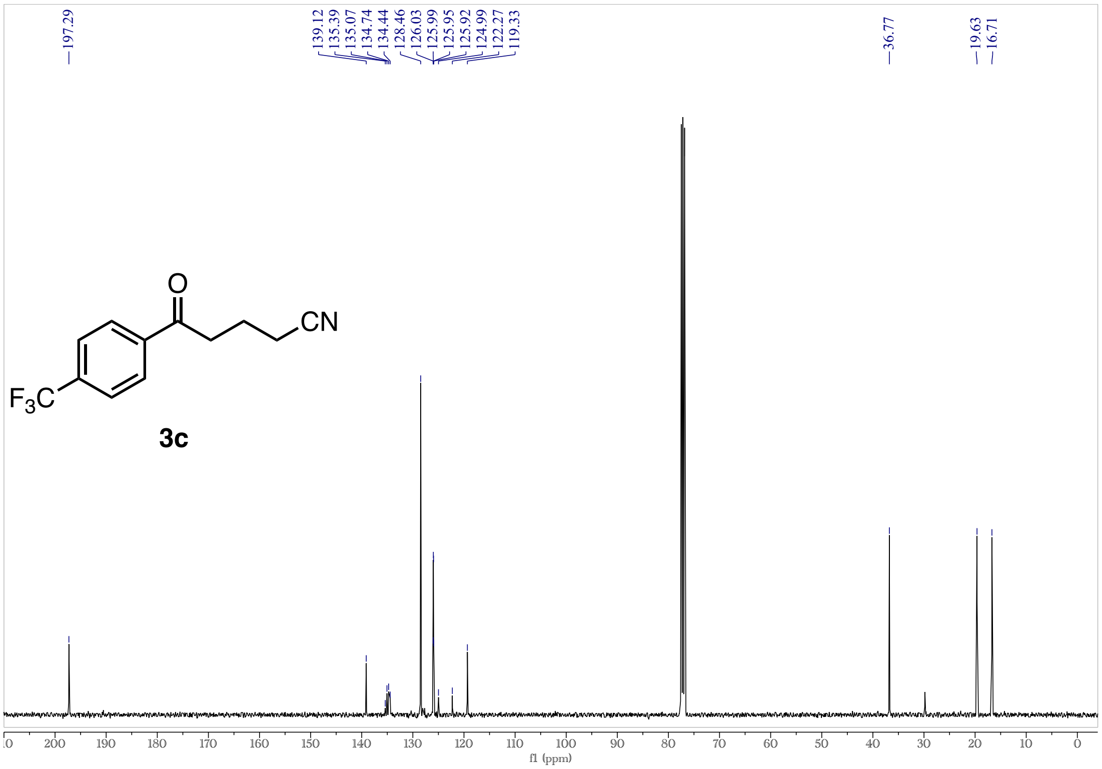

^{13}**C NMR{^{1}H}** (101 MHz, $CDCl_3$)

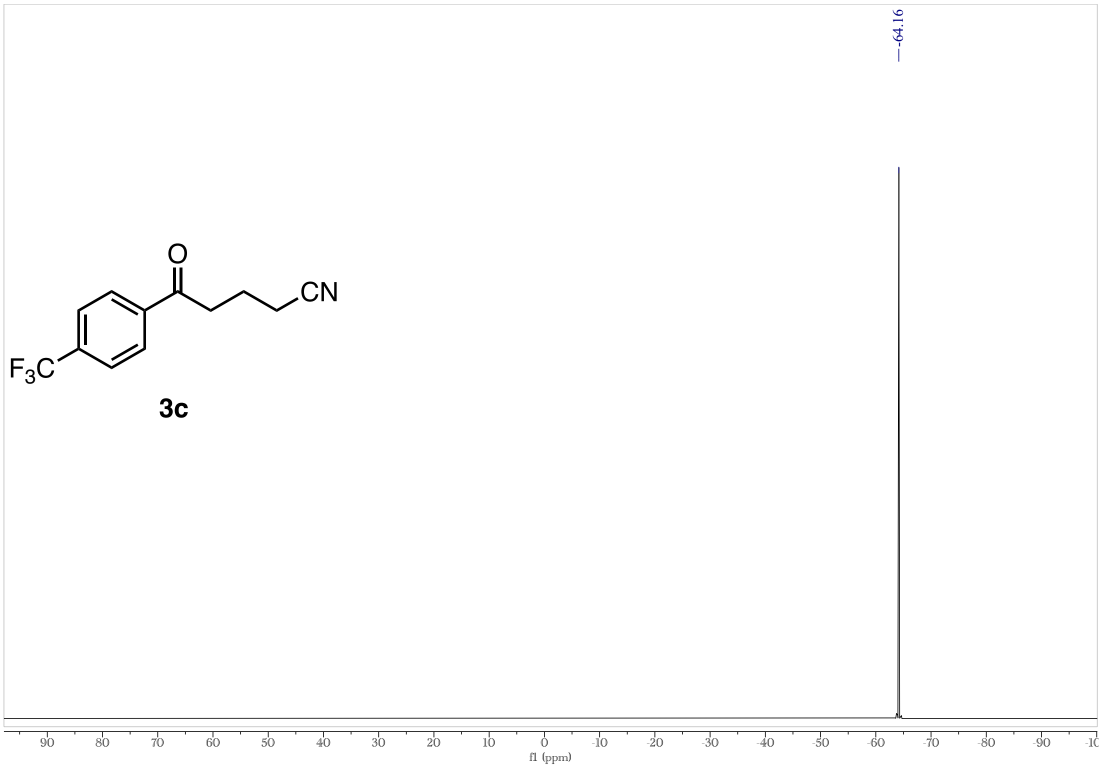

^{19}F NMR (376 MHz, $CDCl_3$)

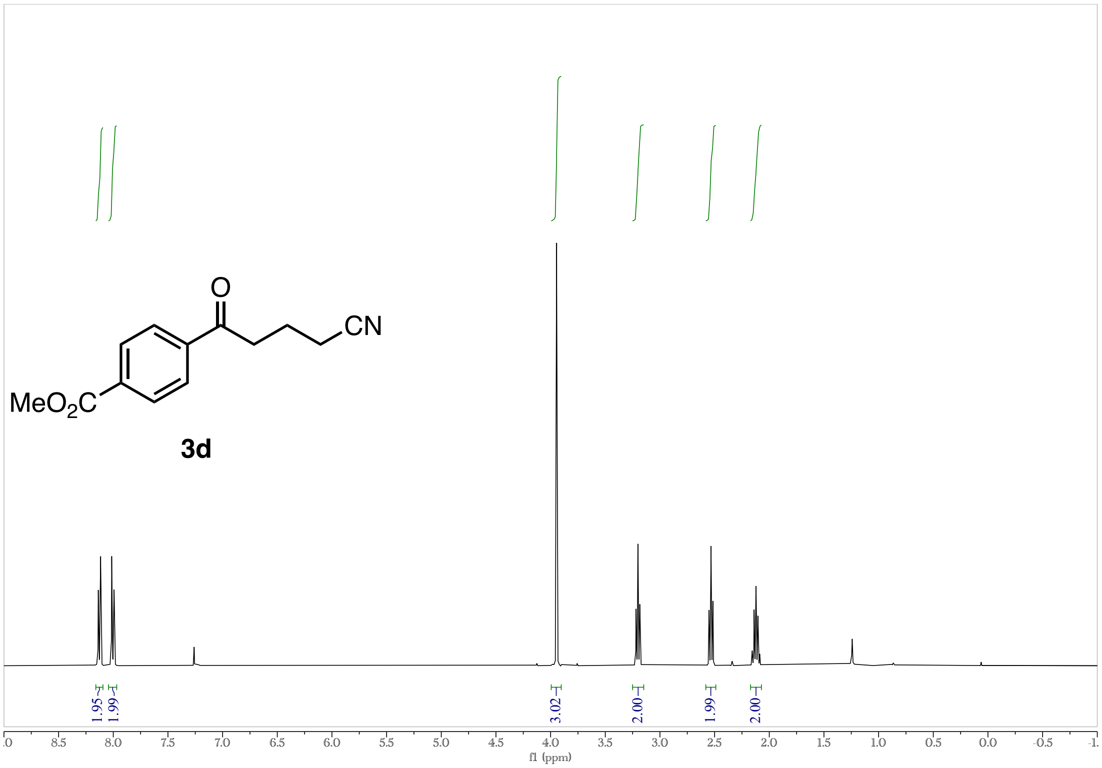

^{1}**H NMR** (400 MHz, CDCl$_3$)

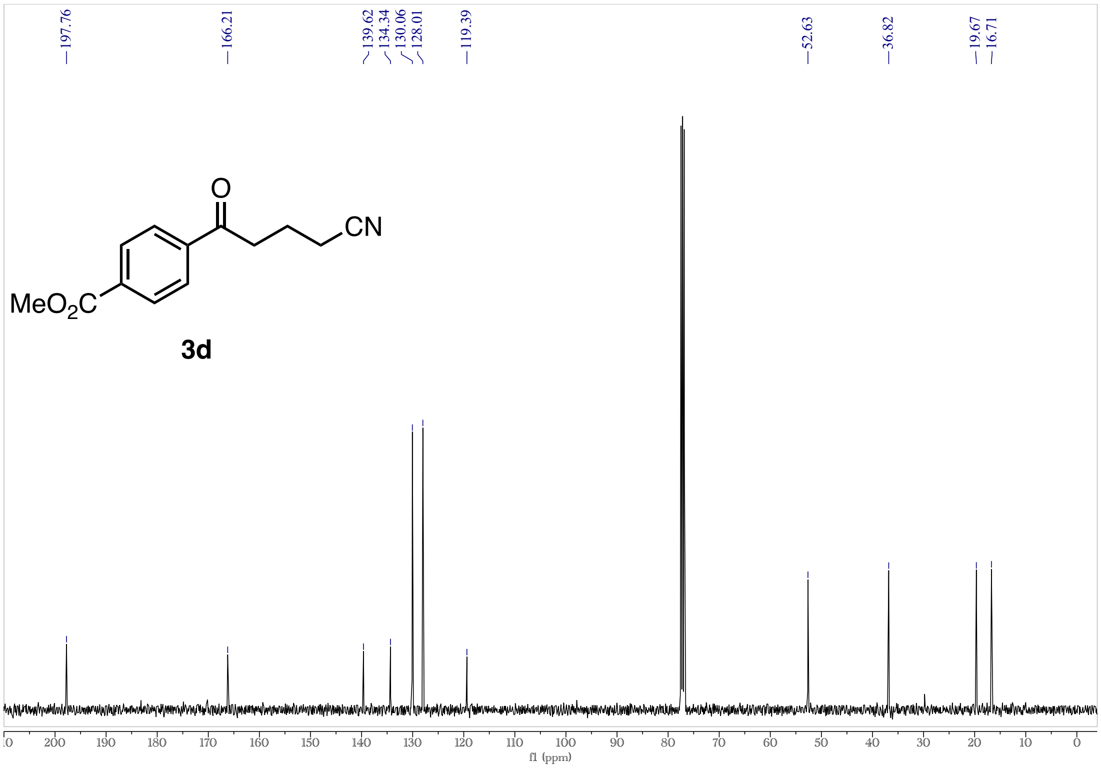

^{13}**C{^{1}H} NMR** (101 MHz, CDCl$_3$)

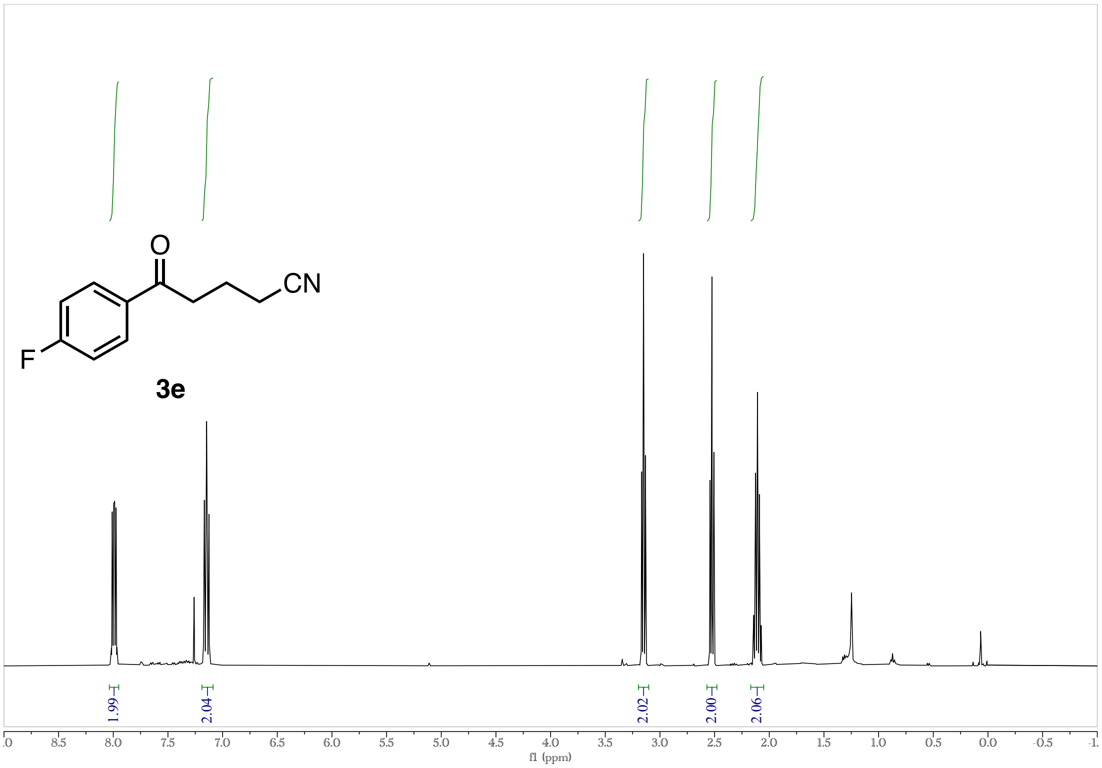

^{1}**H NMR** (400 MHz, $CDCl_3$)

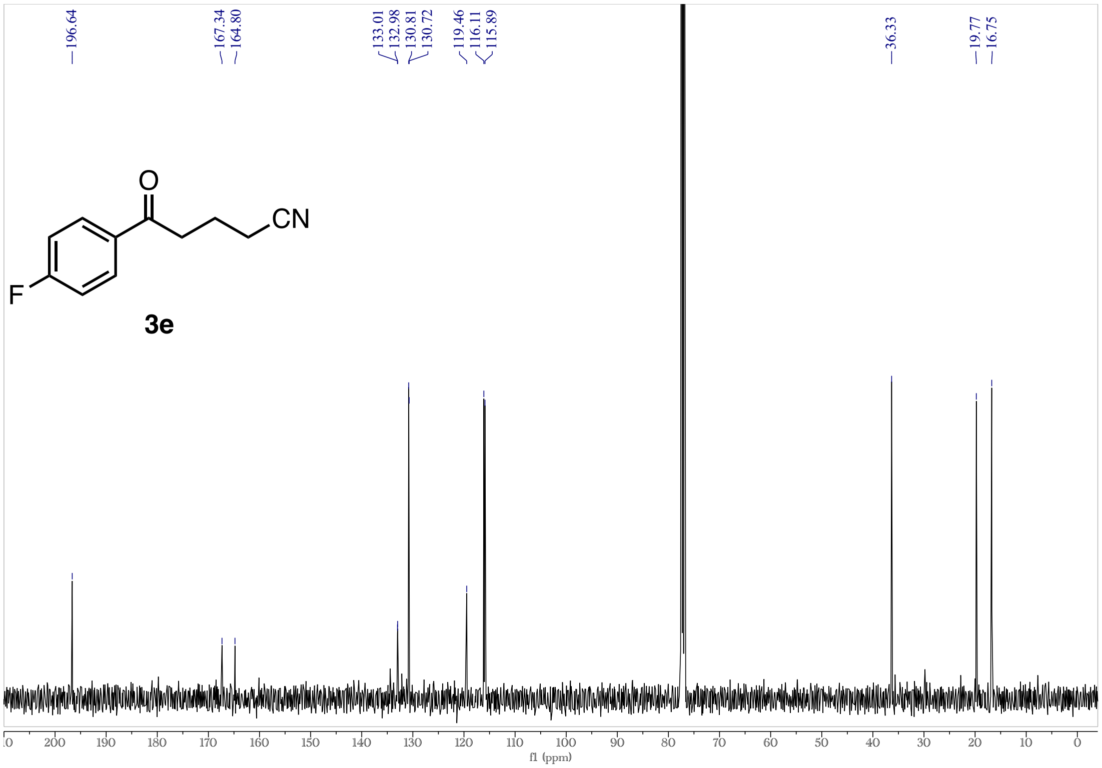

^{13}**C NMR{**^{1}**H}** (101 MHz, $CDCl_3$)

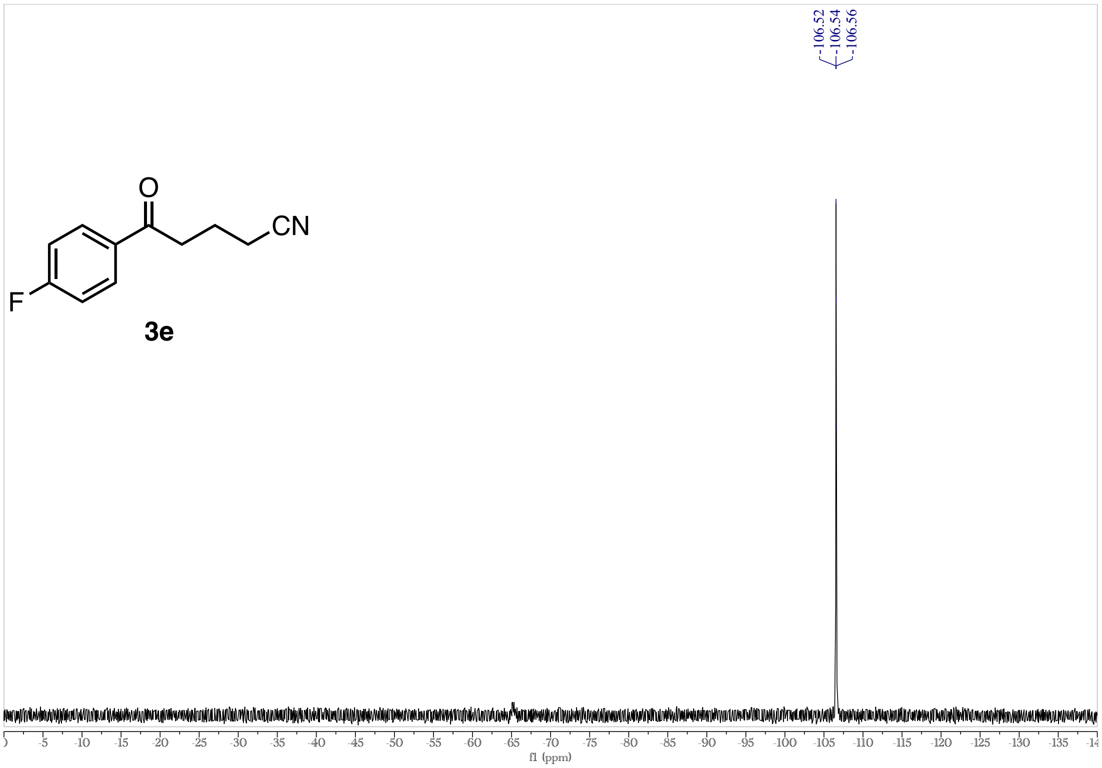

^{19}F NMR (376 MHz, $CDCl_3$)

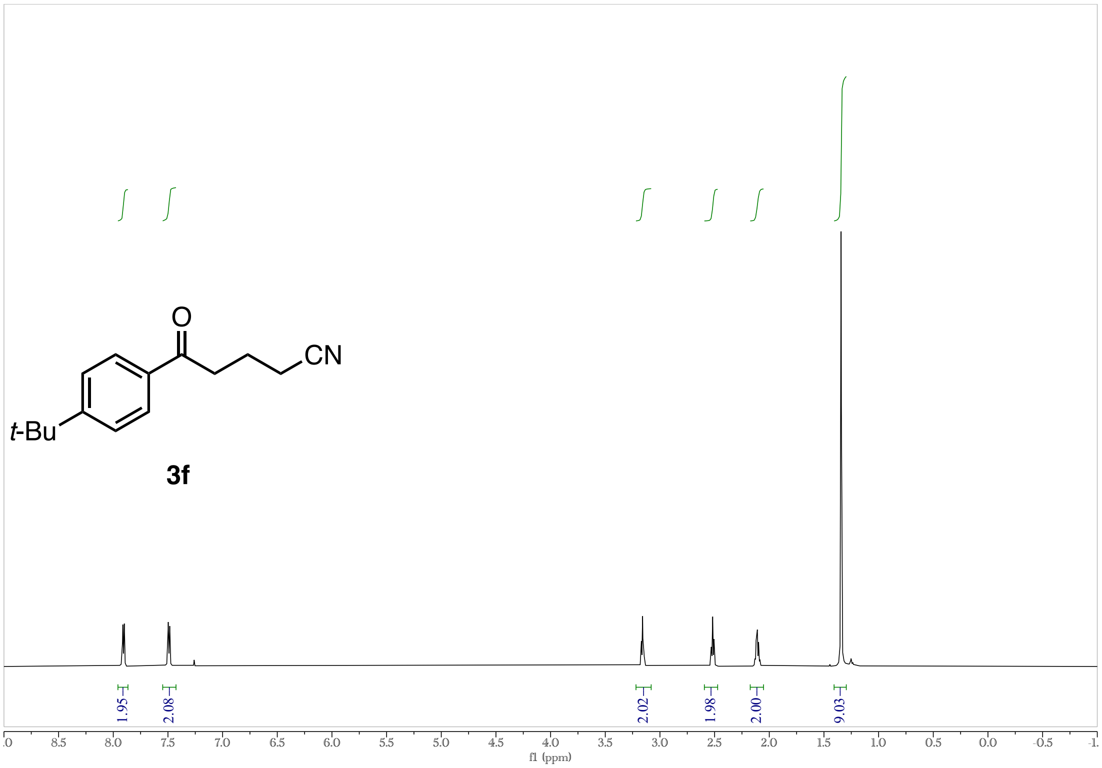

^{1}**H NMR** (600 MHz, $CDCl_3$)

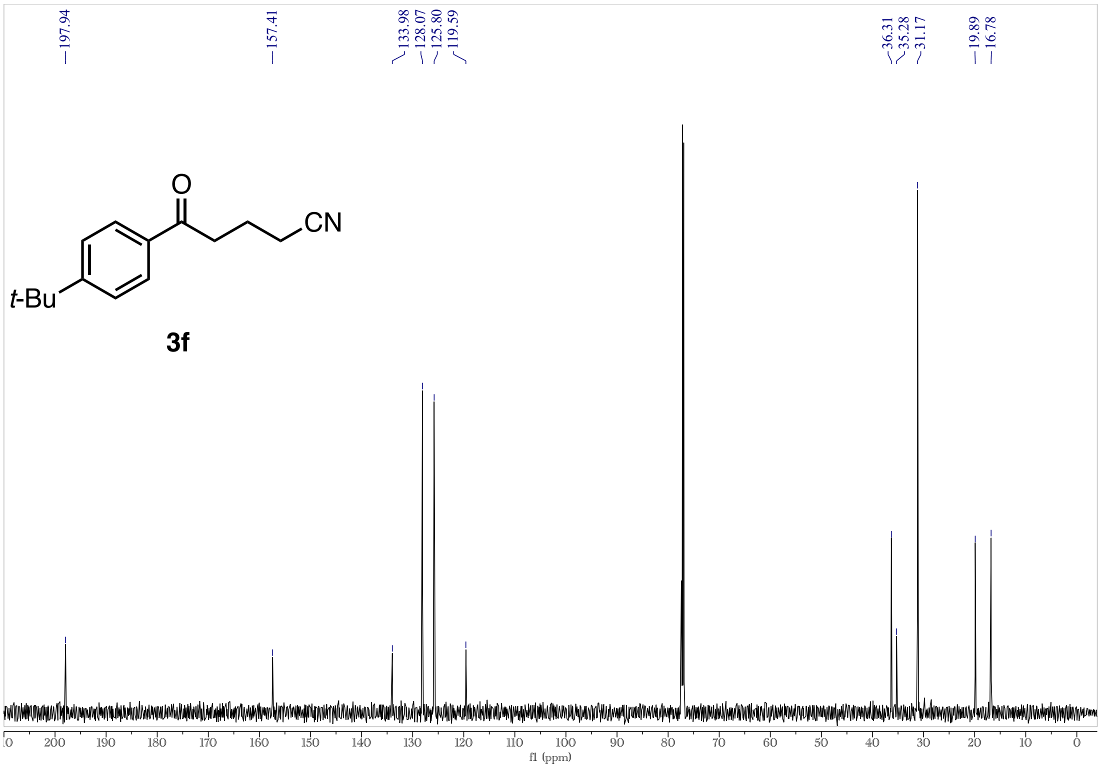

^{13}**C NMR{^{1}H}** (151 MHz, $CDCl_3$)

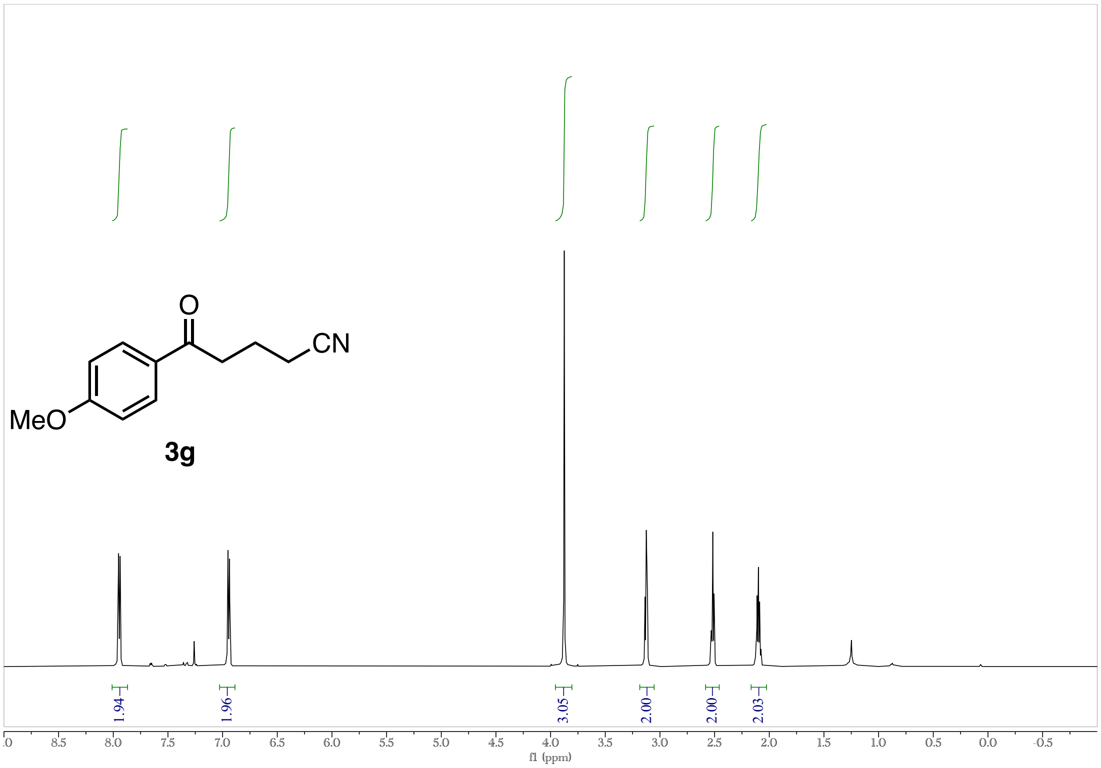

^{1}H NMR (600 MHz, $CDCl_3$)

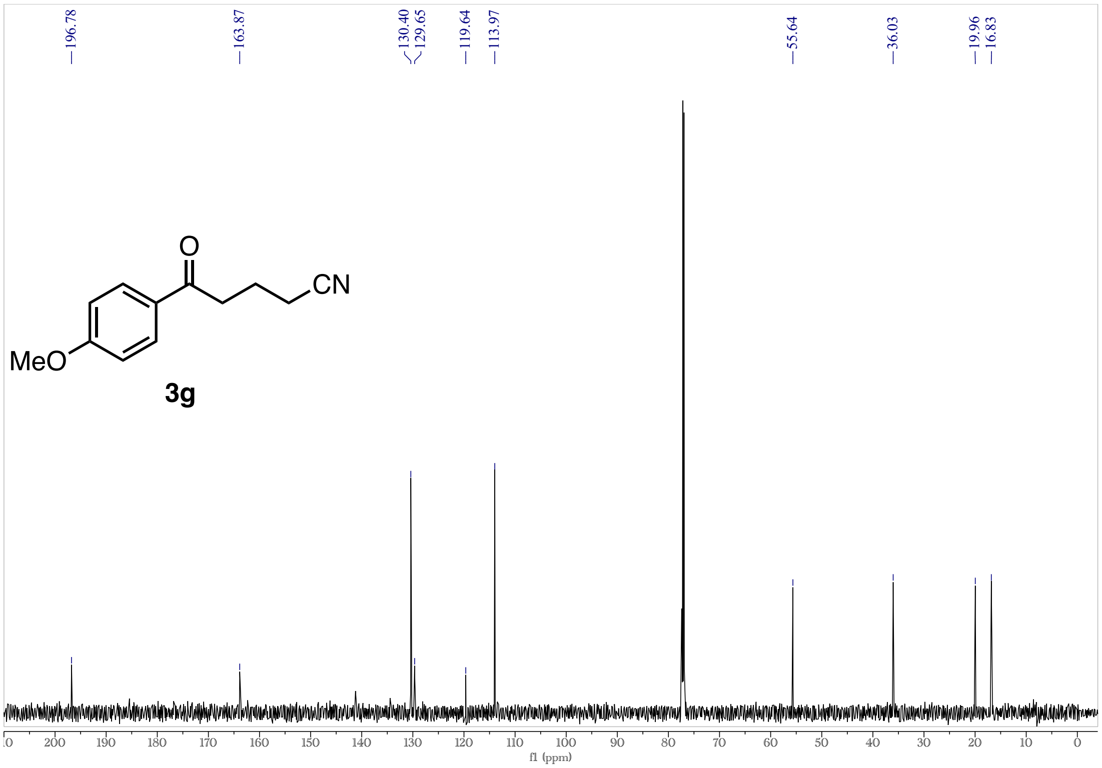

^{13}C NMR{^{1}H} (151 MHz, $CDCl_3$)

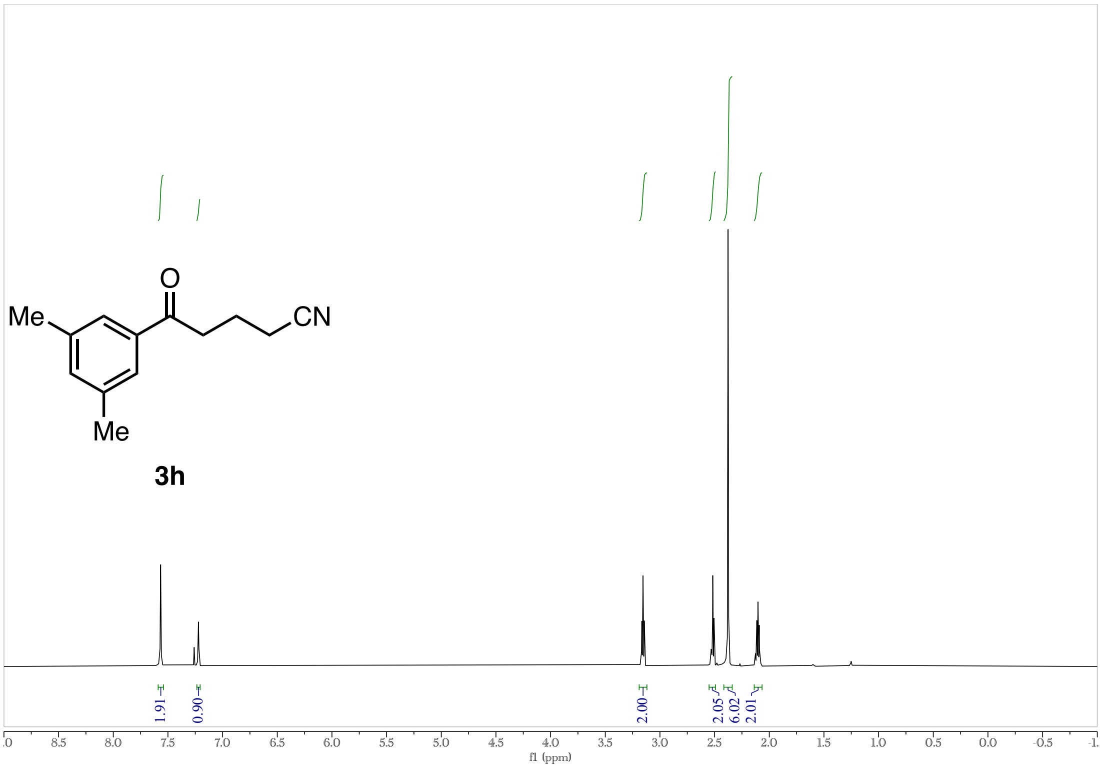

^{1}**H NMR** (600 MHz, $CDCl_3$)

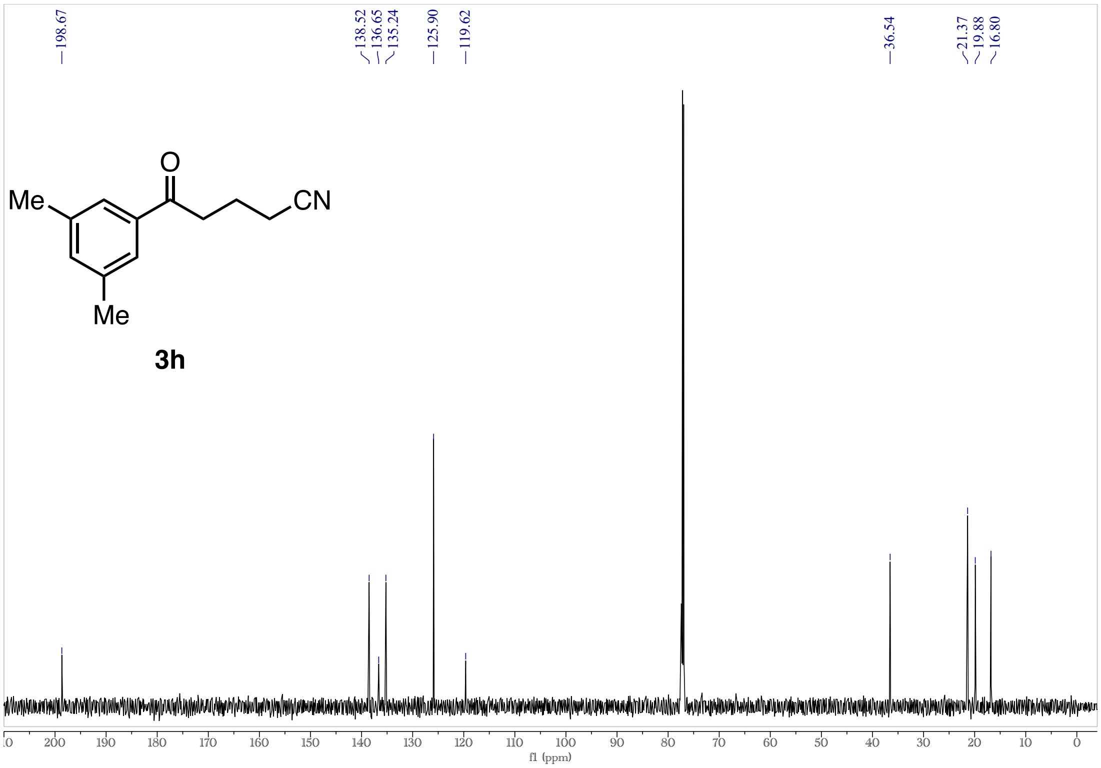

^{13}**C NMR{^{1}H}** (151 MHz, $CDCl_3$)

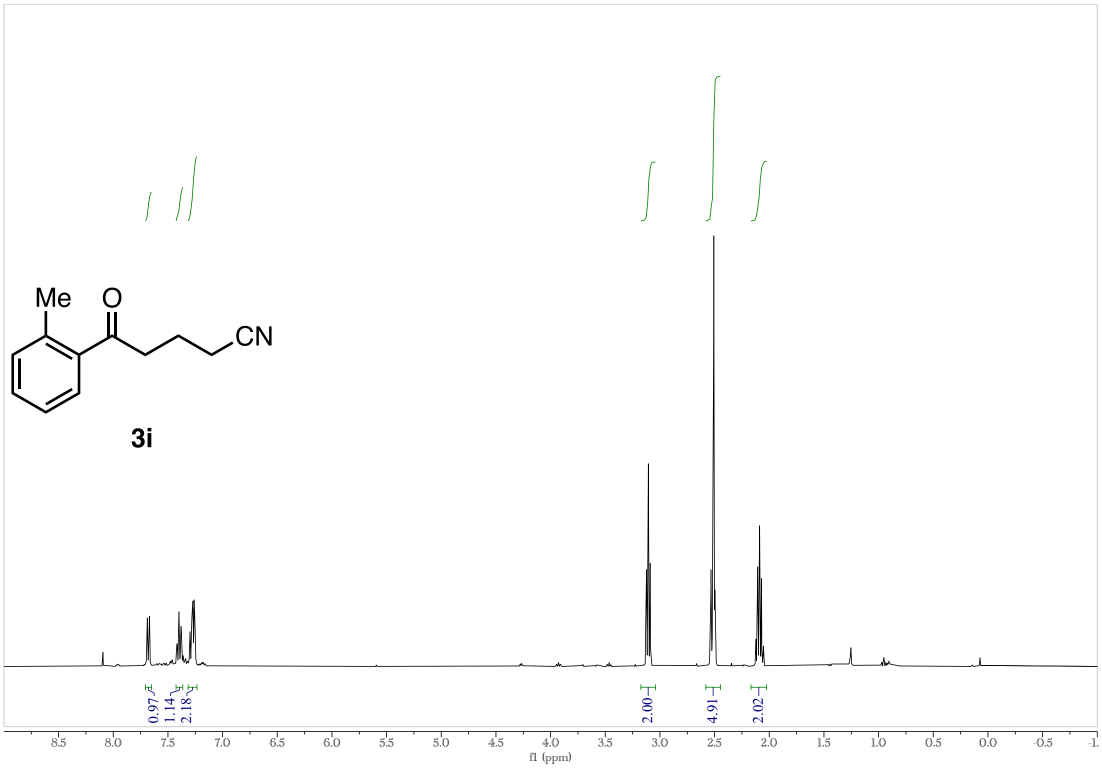

^{1}H NMR (400 MHz, $CDCl_3$)

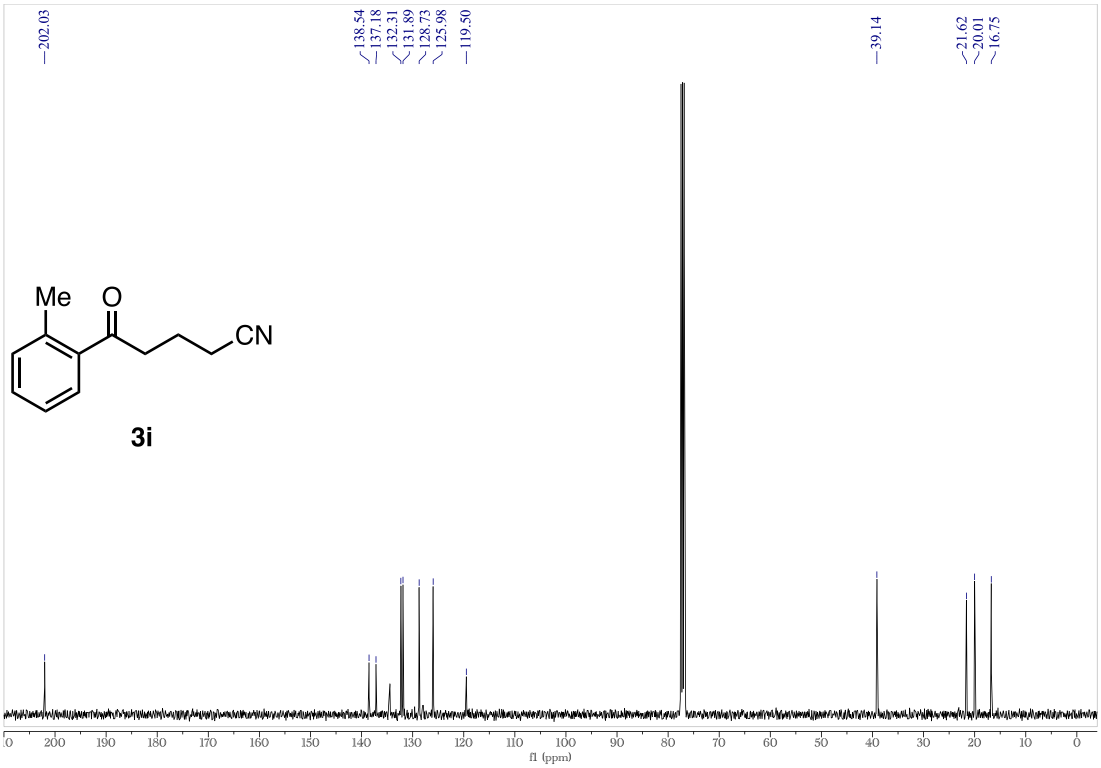

^{13}C NMR{^{1}H} (101 MHz, $CDCl_3$)

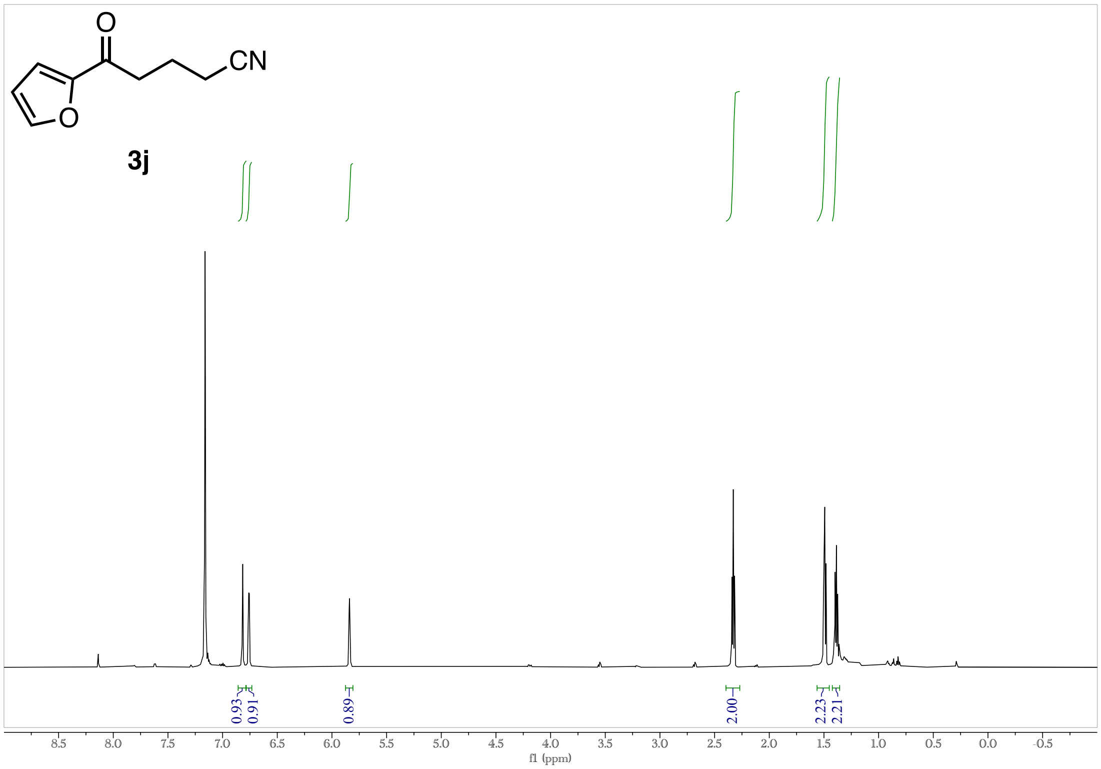

^{1}H NMR (600 MHz, C_6D_6)

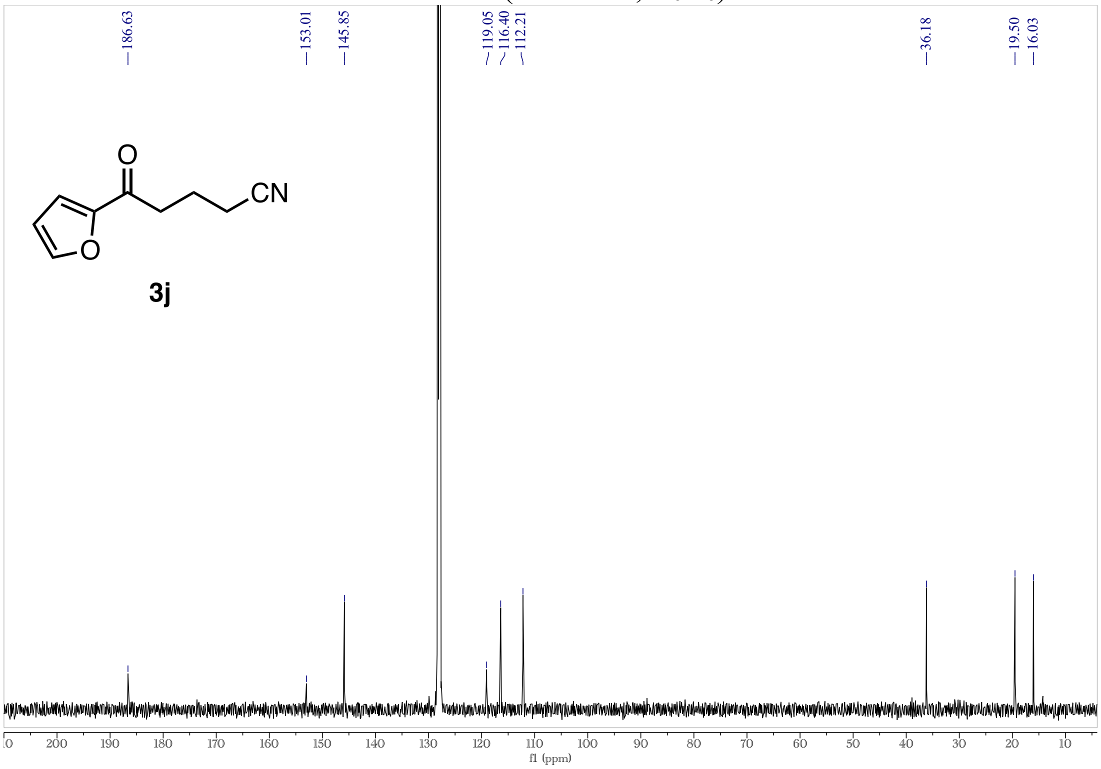

^{13}C{^{1}H} NMR (151 MHz, C_6D_6)

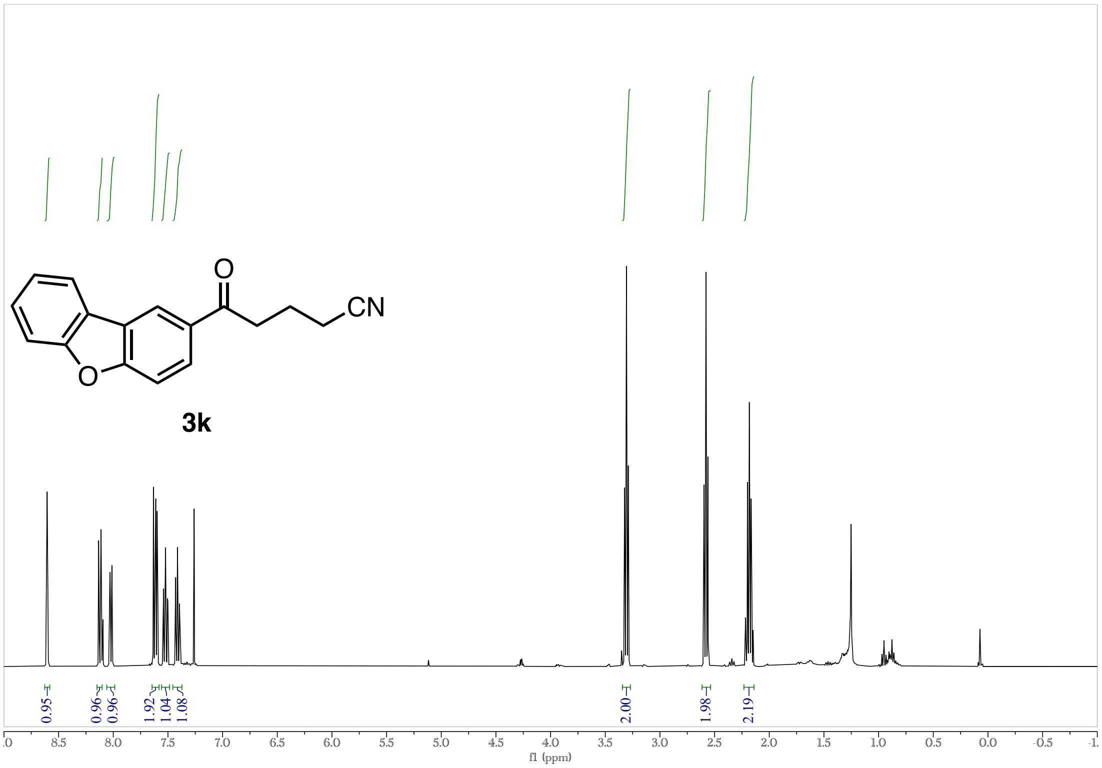

^{1}H NMR (400 MHz, $CDCl_3$)

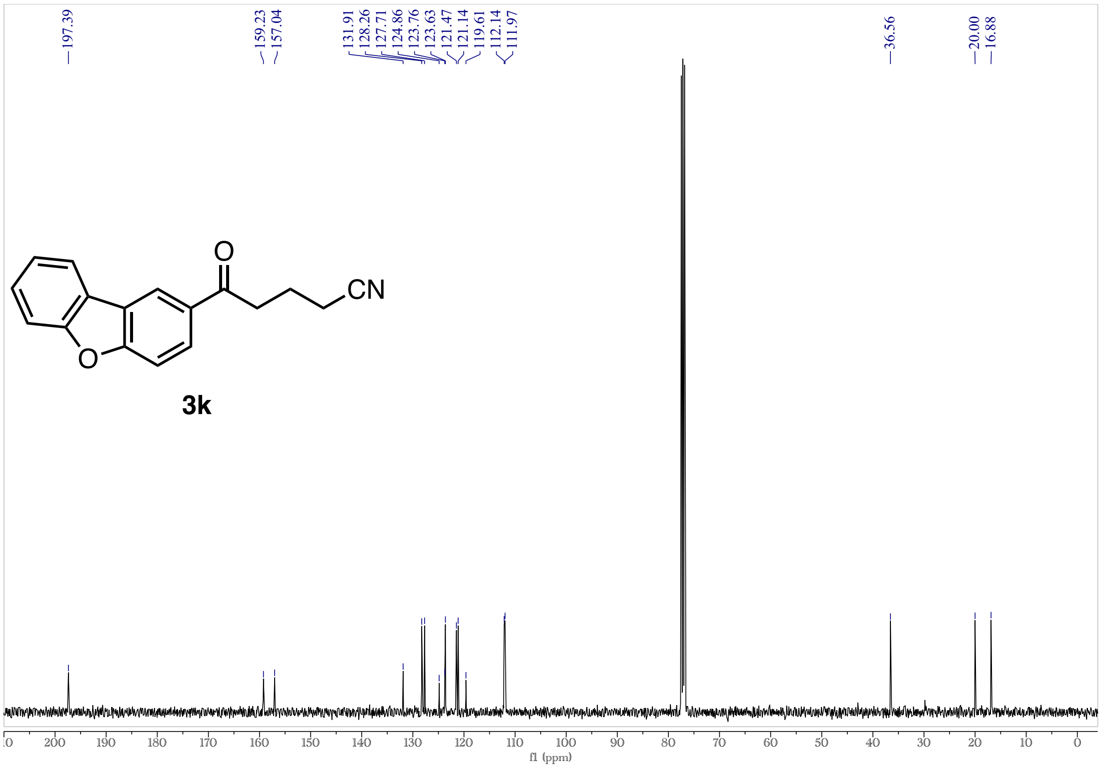

^{13}C{^{1}H} NMR (101 MHz, $CDCl_3$)

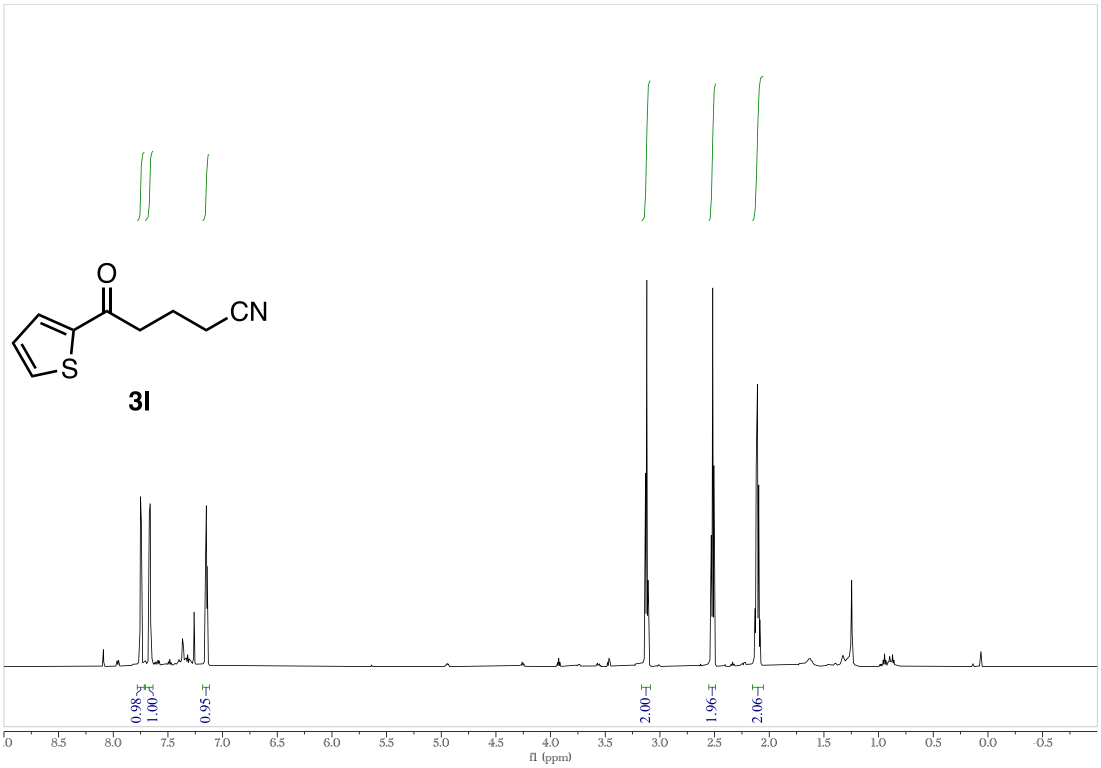

^{1}H NMR (600 MHz, $CDCl_3$)

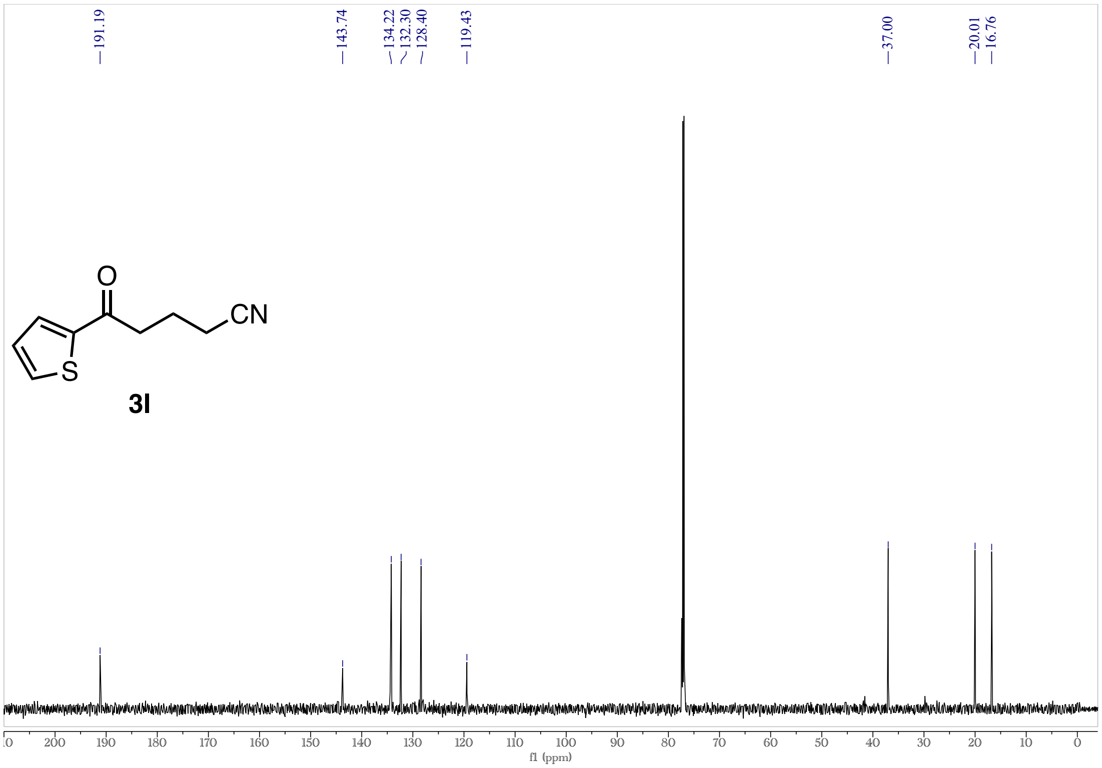

^{13}C{^{1}H} NMR (151 MHz, $CDCl_3$)

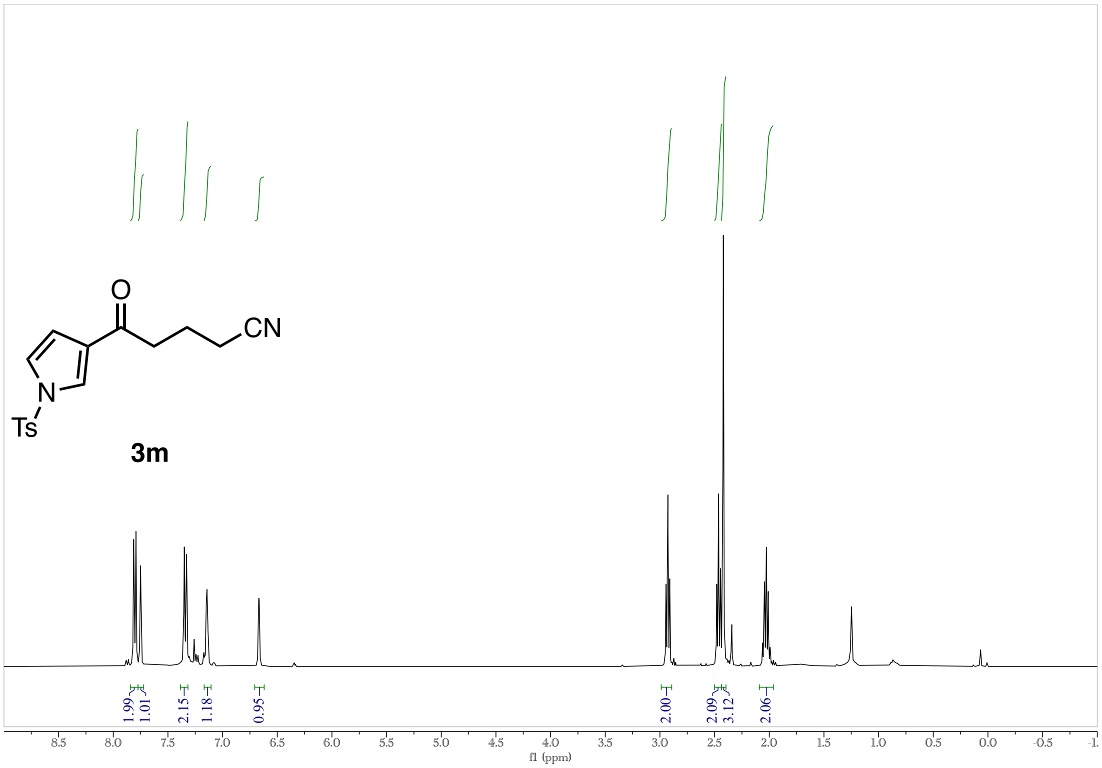

^{1}H NMR (400 MHz, $CDCl_3$)

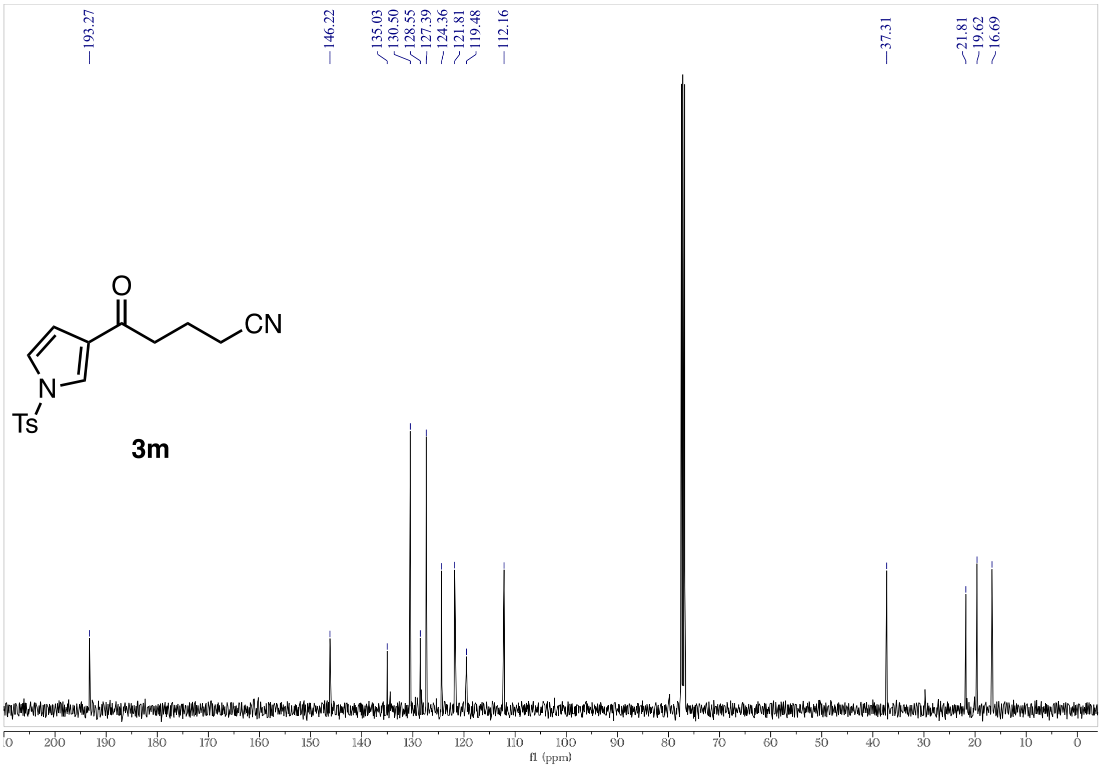

^{13}C{^{1}H} NMR (101 MHz, $CDCl_3$)

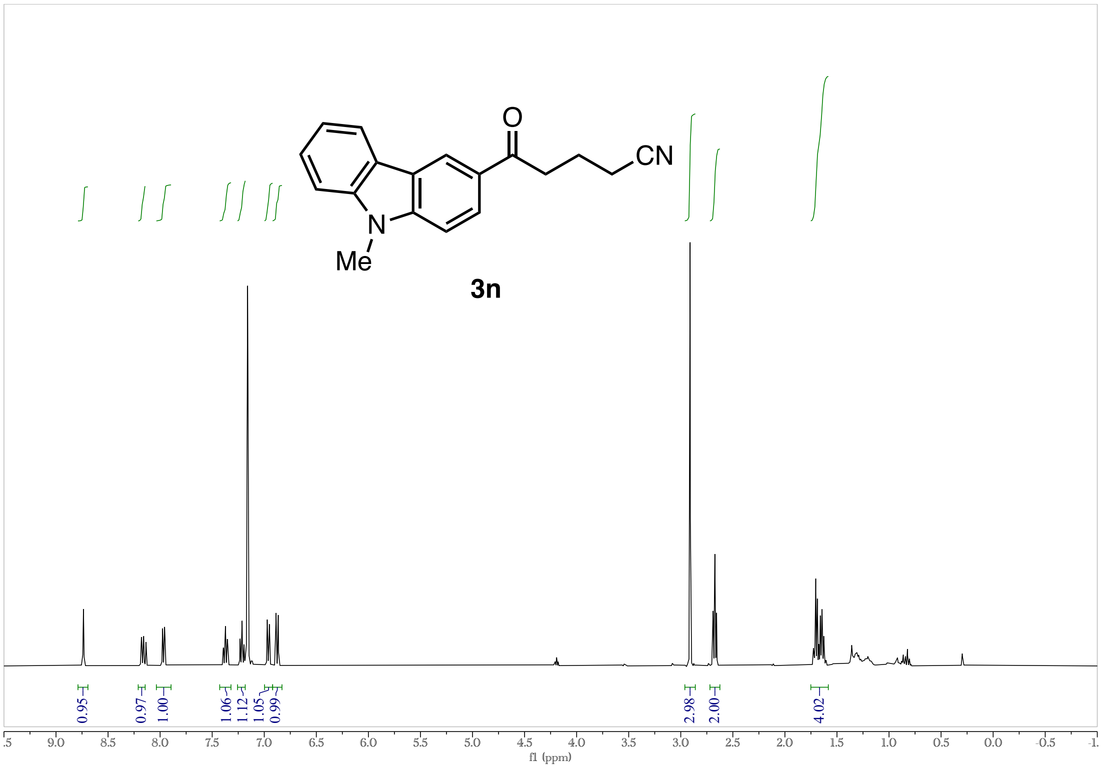

1H NMR (400 MHz, C_6D_6)

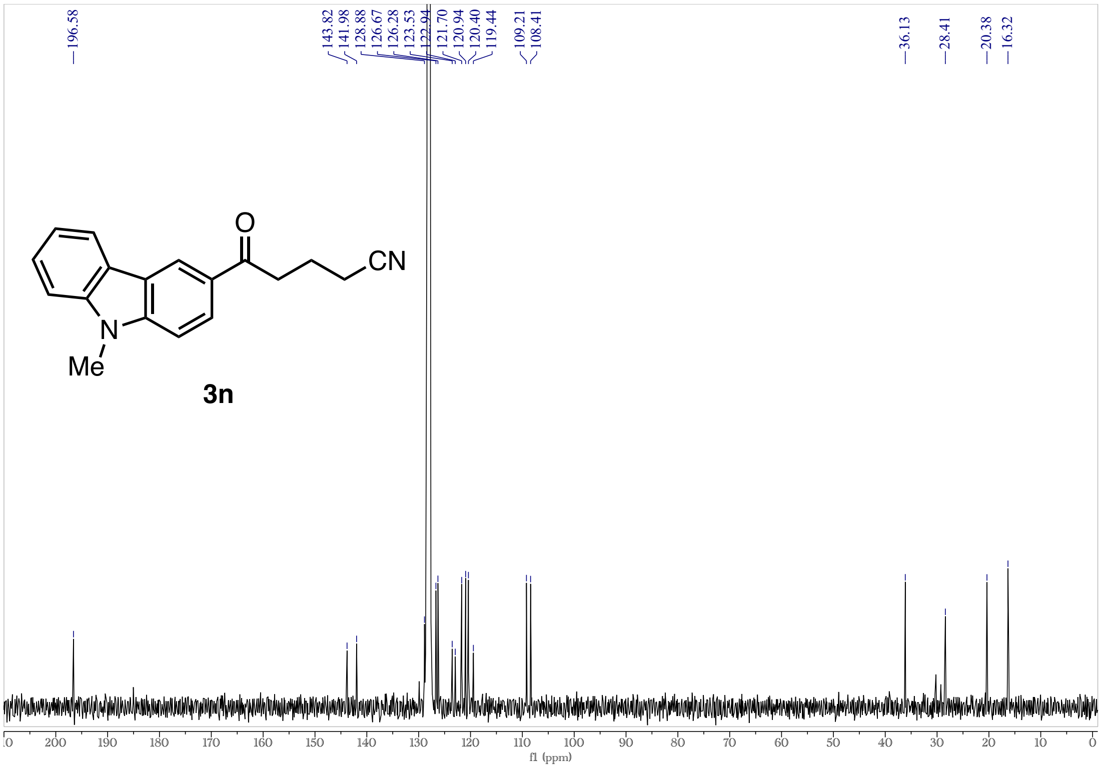

$^{13}C\{^1H\}$ (101 MHz, C_6D_6)

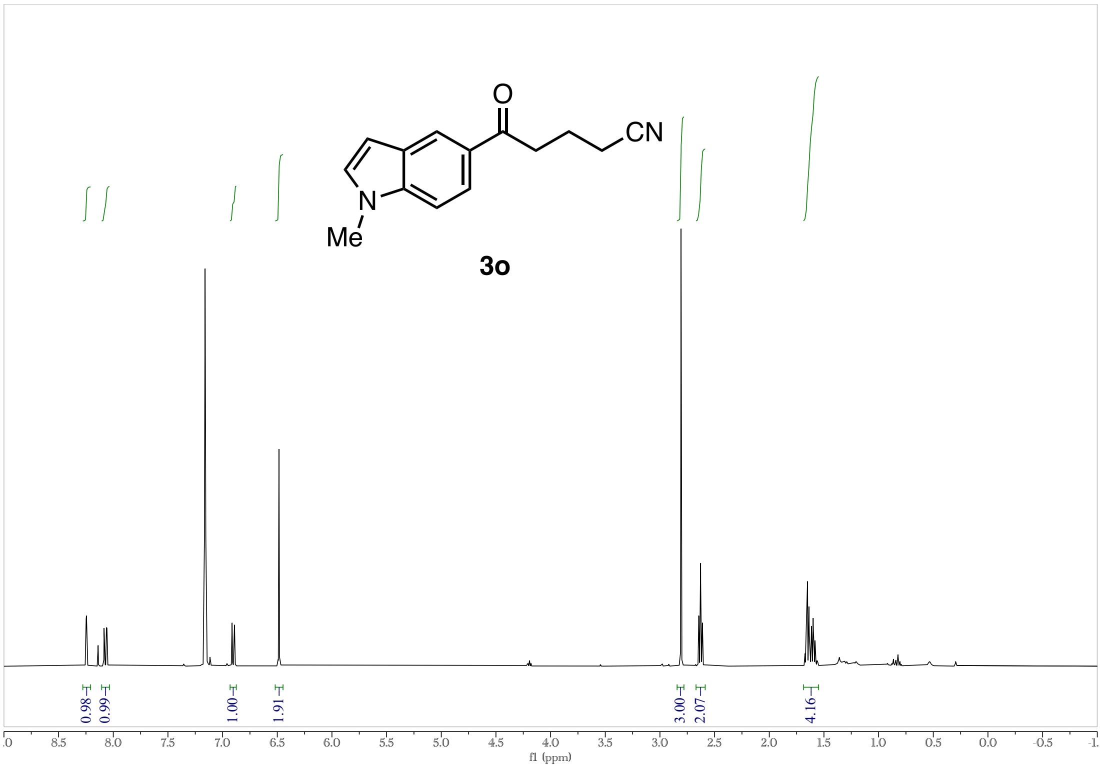

^{1}H NMR (400 MHz, C_6D_6)

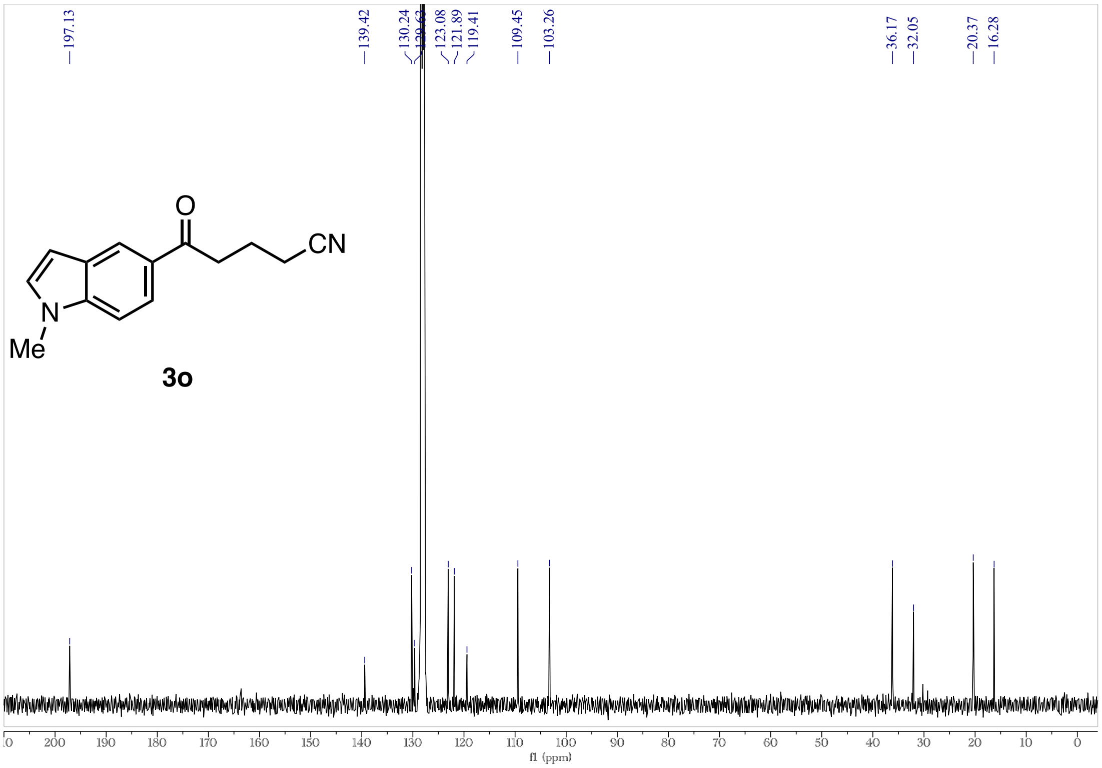

^{13}C{^{1}H} NMR (101 MHz, C_6D_6)

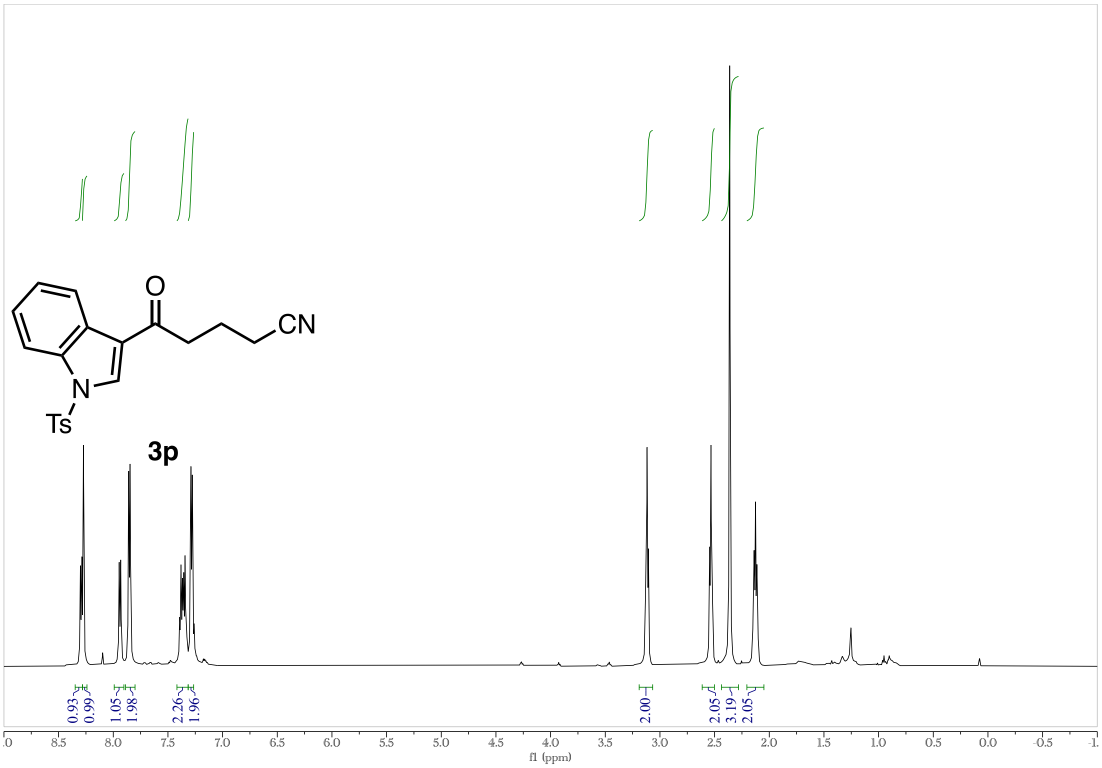

^{1}H NMR (600 MHz, $CDCl_3$)

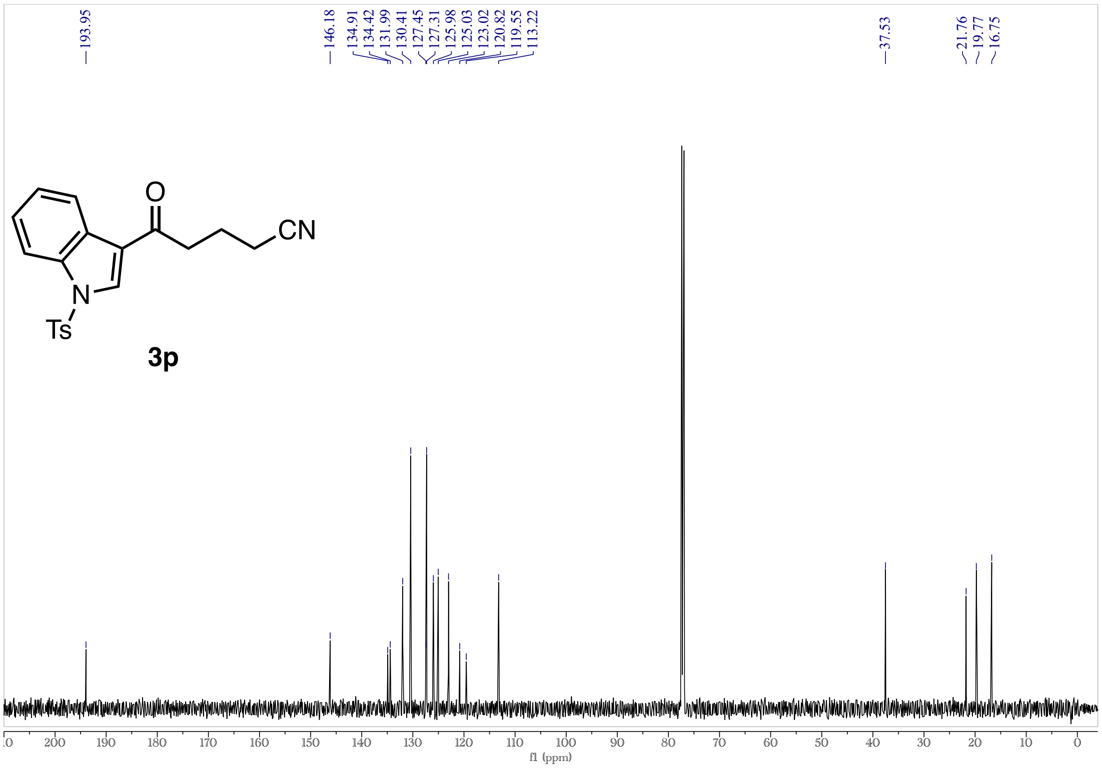

^{13}C{^{1}H} NMR (151 MHz, $CDCl_3$)

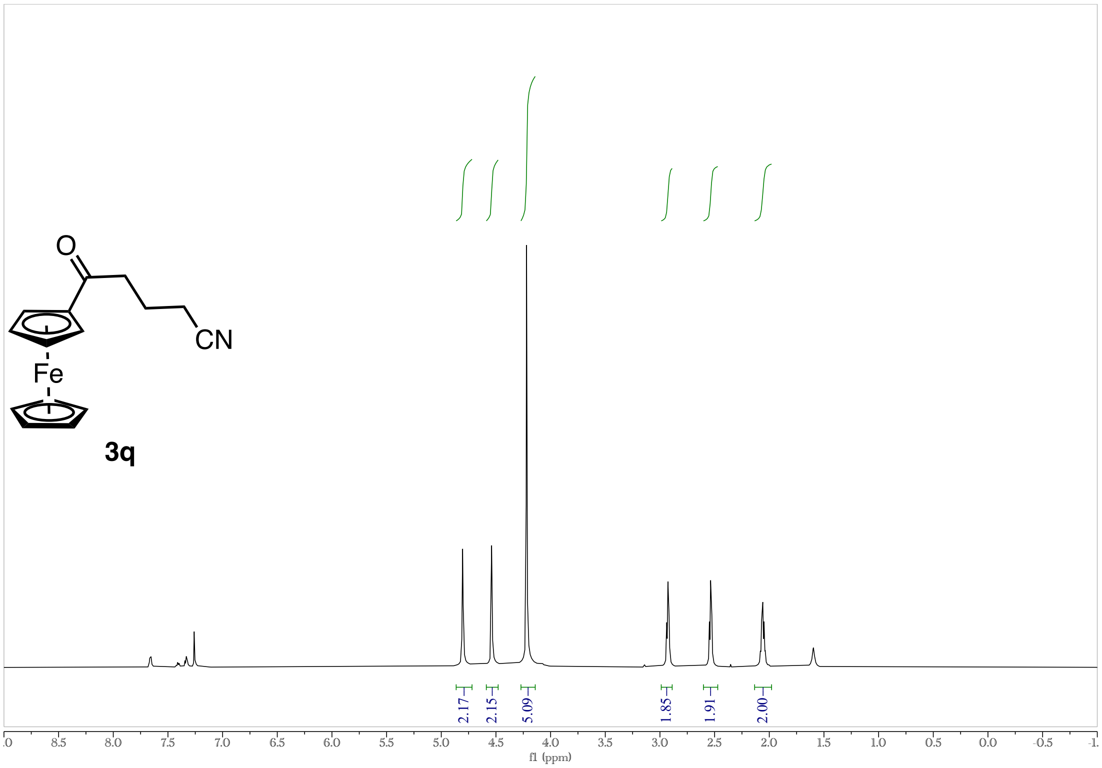

^{1}**H NMR** (600 MHz, CDCl$_3$)

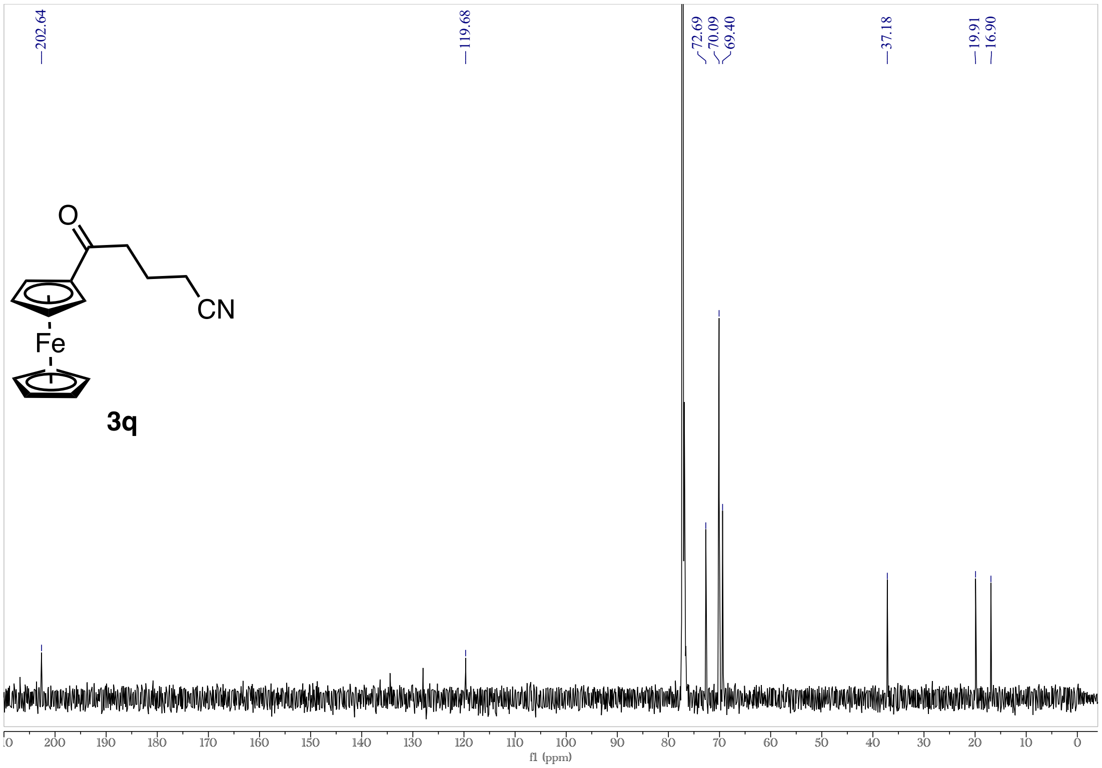

^{13}**C{^{1}H} NMR** (151 MHz, CDCl$_3$)

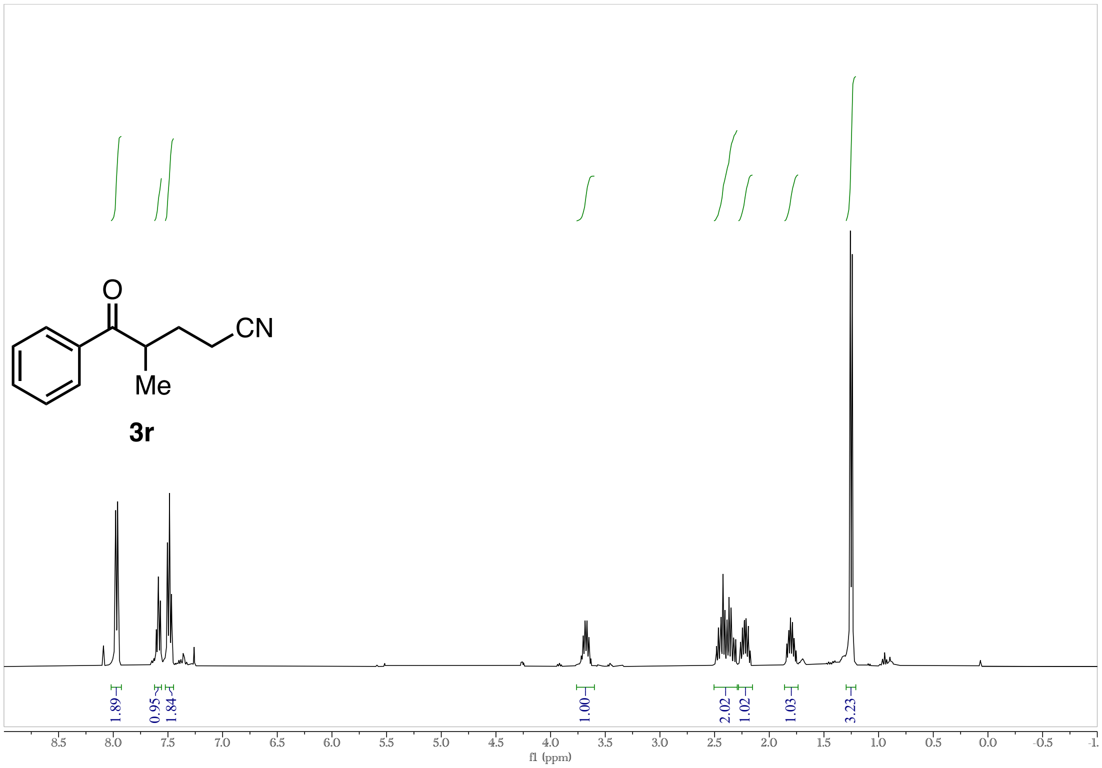

^{1}**H NMR** (400 MHz, $CDCl_3$)

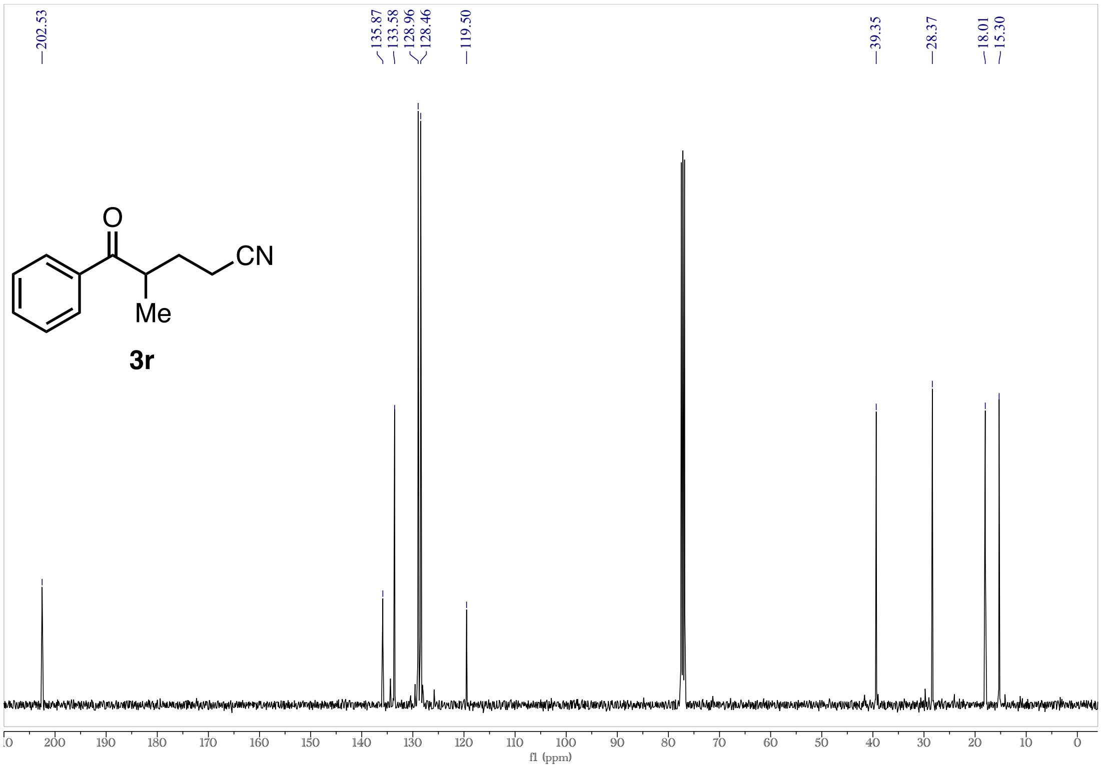

^{13}**C{^{1}H} NMR** (101 MHz, $CDCl_3$)

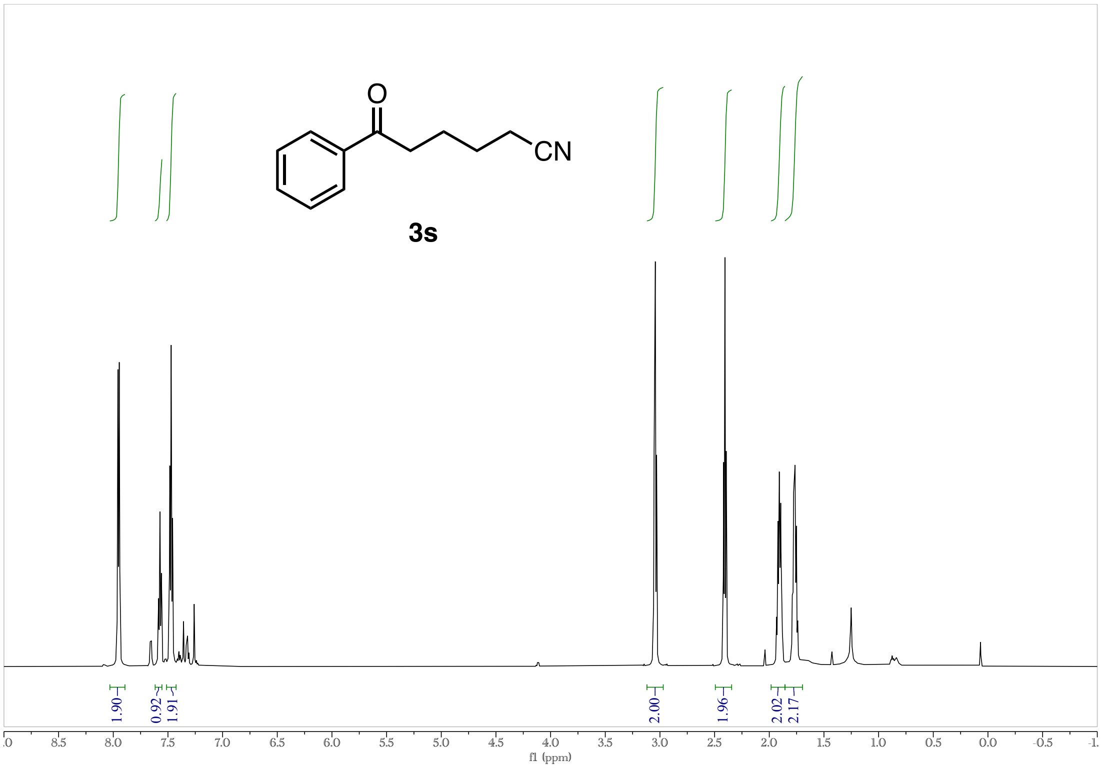

^{1}H NMR (600 MHz, $CDCl_3$)

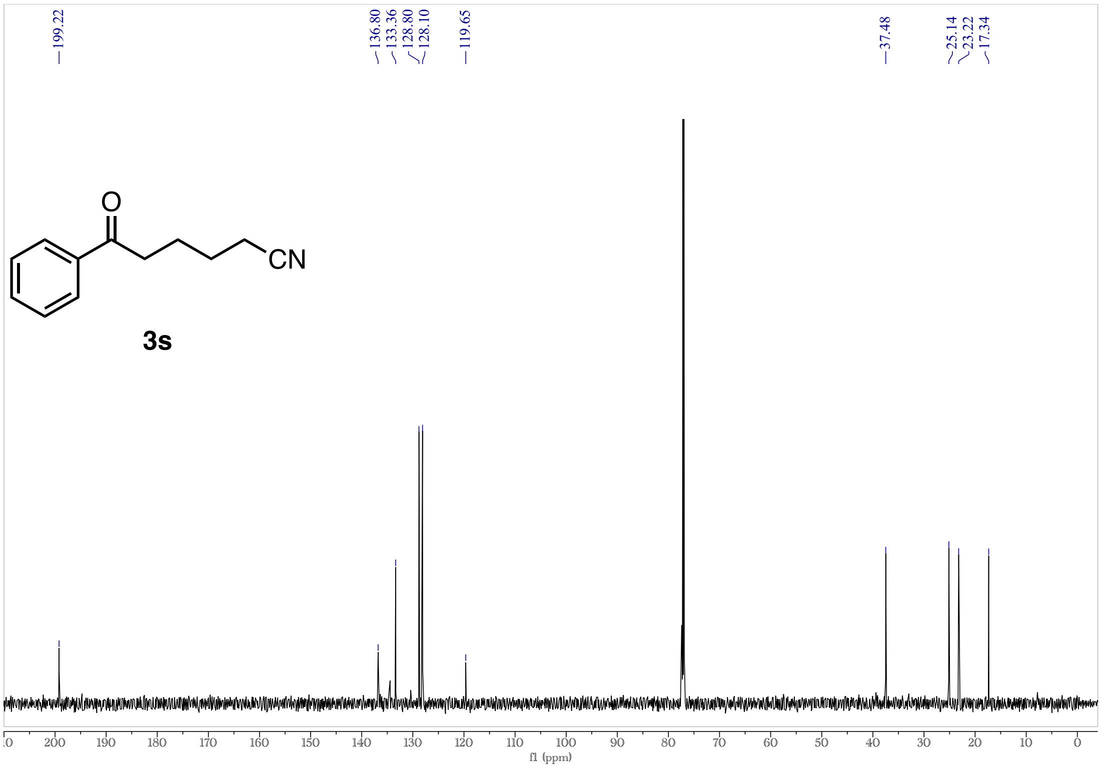

^{13}C{^{1}H} NMR (151 MHz, $CDCl_3$)

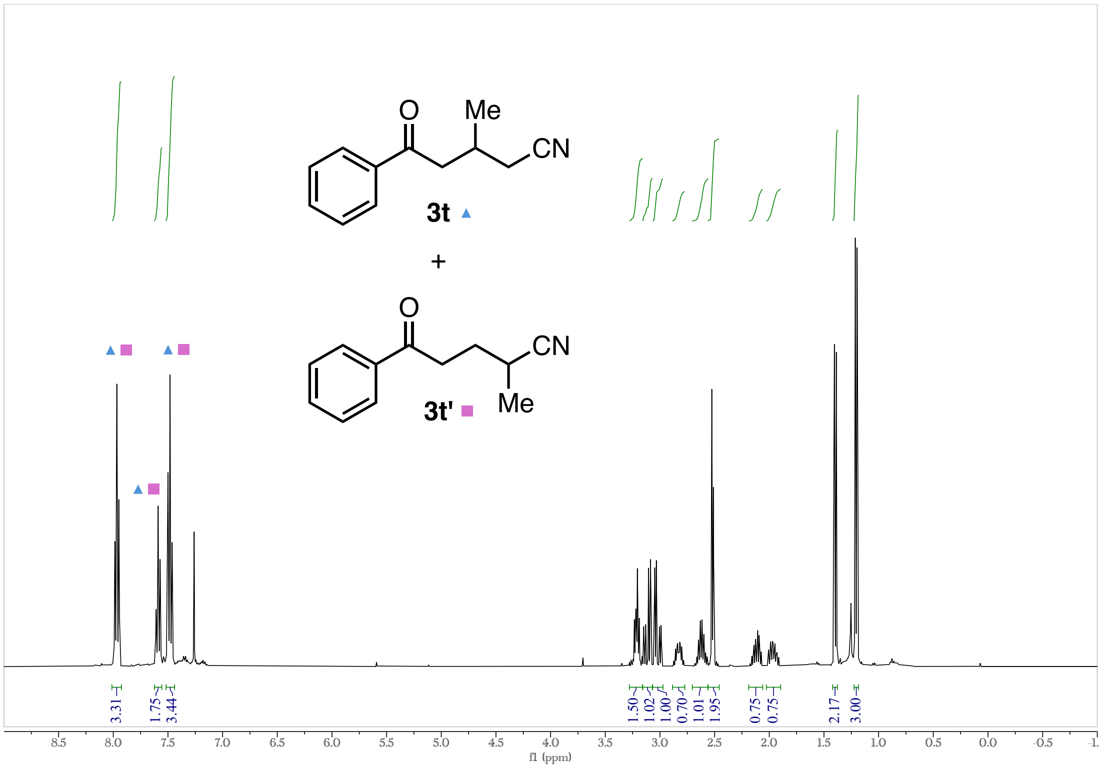

^{1}**H NMR** (400 MHz, $CDCl_3$)

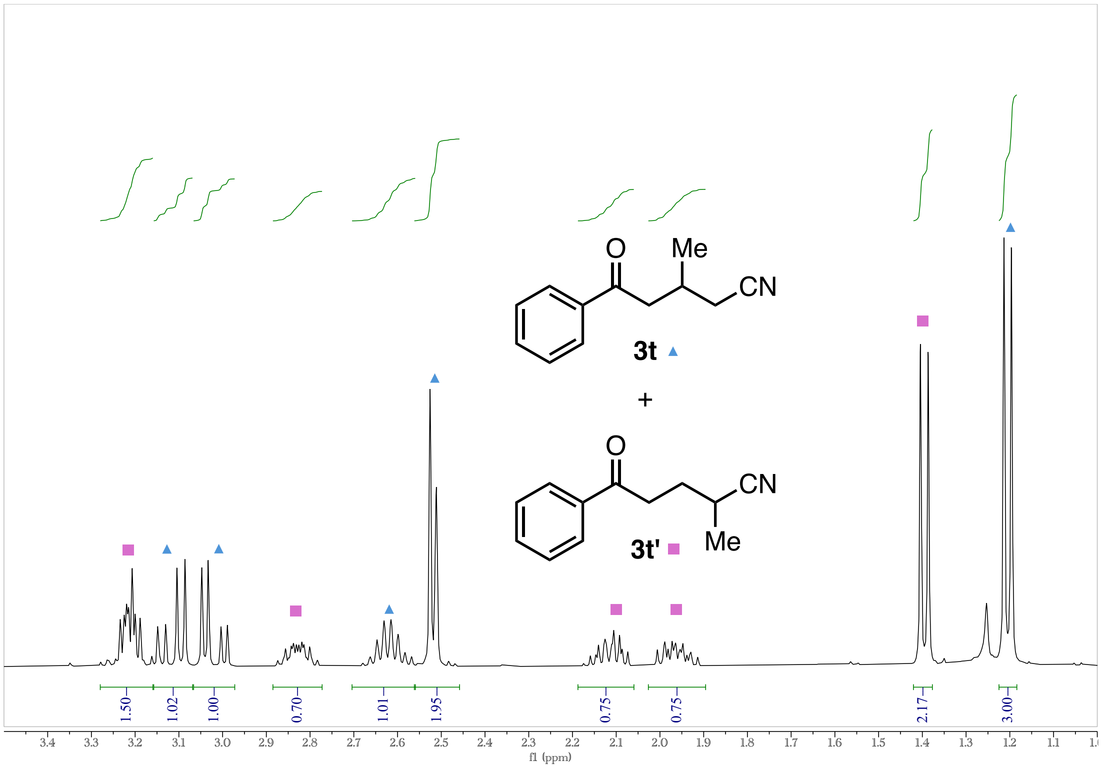

^{1}**H NMR** (400 MHz, $CDCl_3$) alkyl region zoom

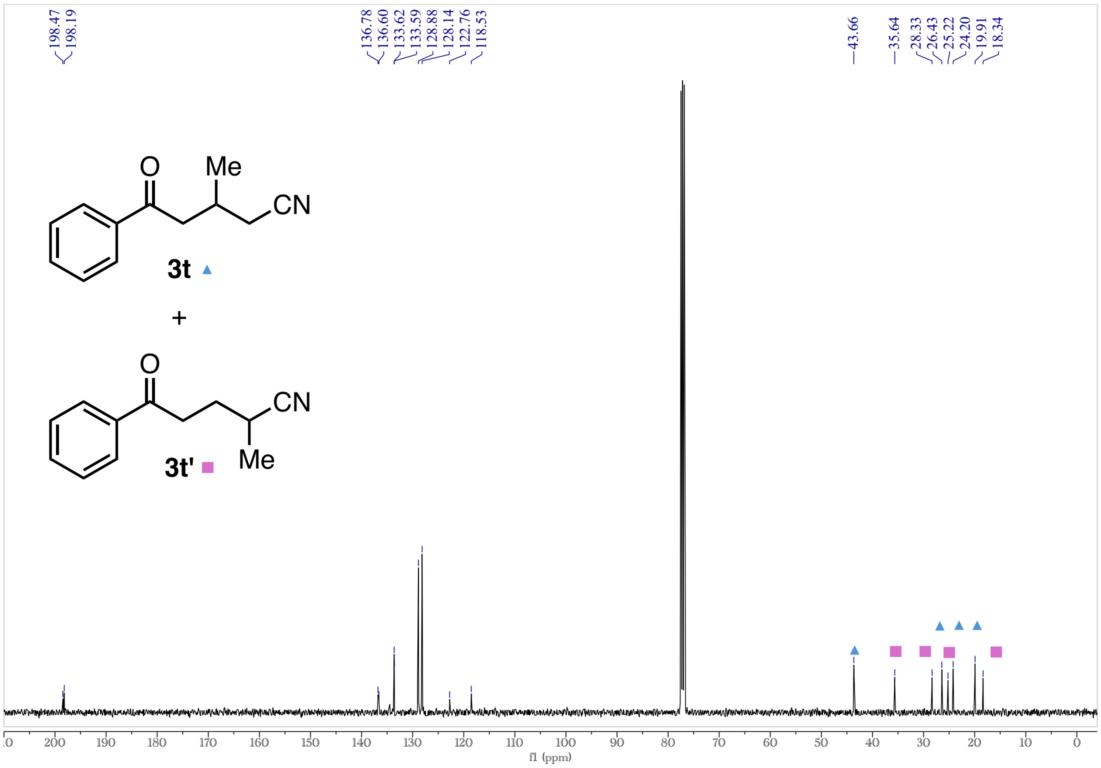

^{13}C{^{1}H} **NMR** (101 MHz, $CDCl_3$)

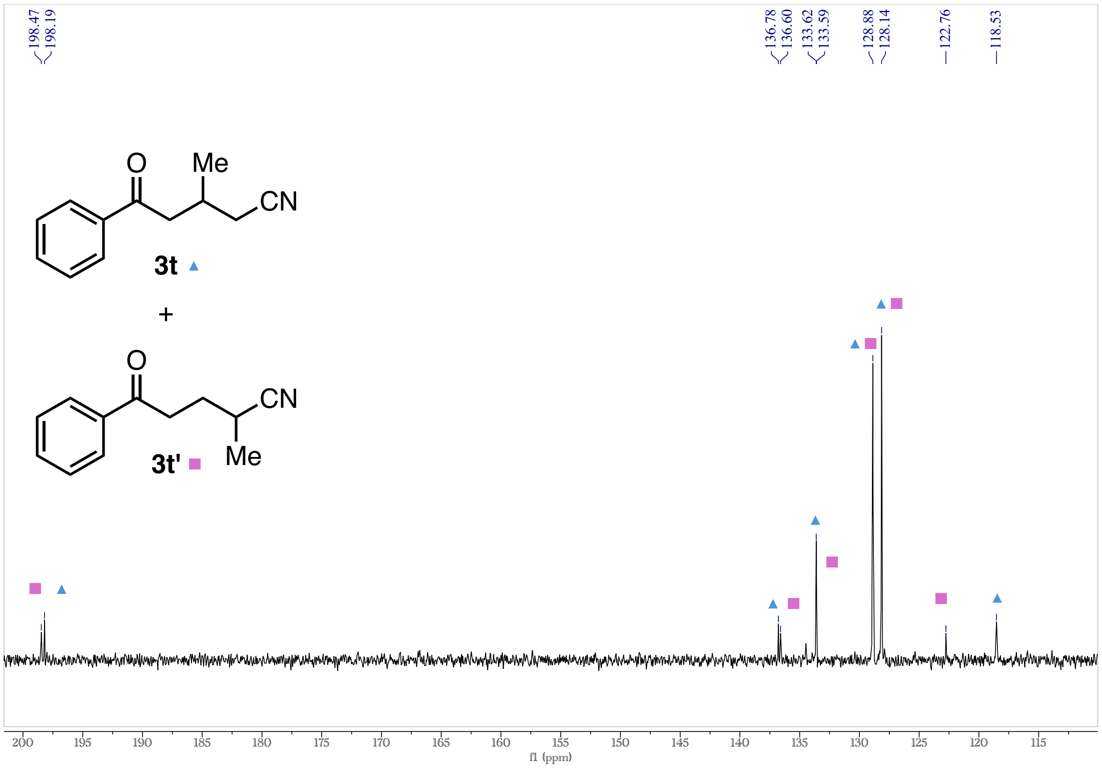

^{13}C{^{1}H} **NMR** (101 MHz, $CDCl_3$) aromatic region zoom

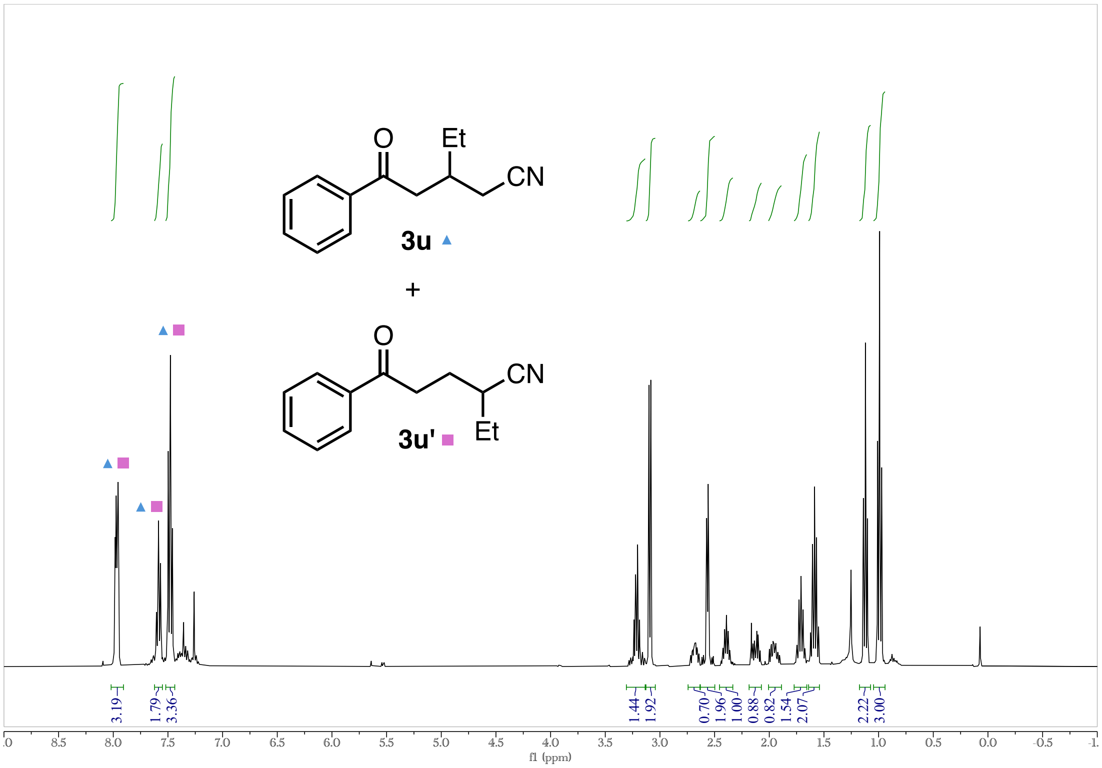

^{1}**H NMR** (400 MHz, $CDCl_3$)

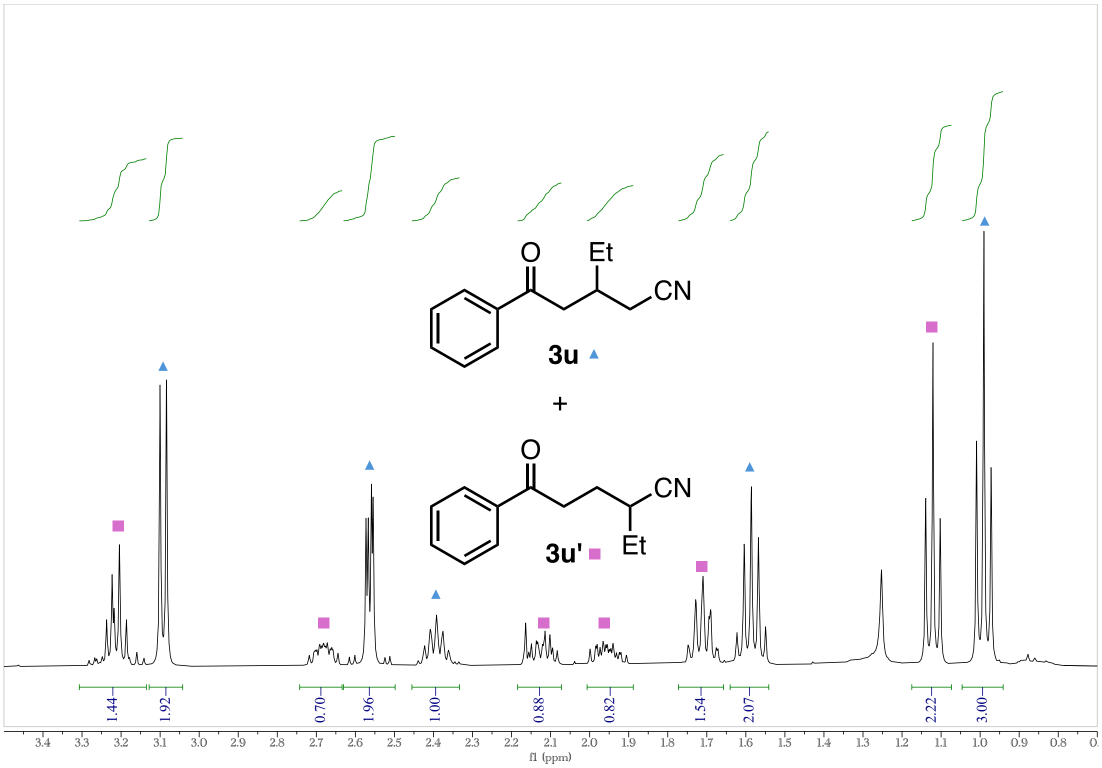

^{1}**H NMR** (400 MHz, $CDCl_3$) alkyl region zoom

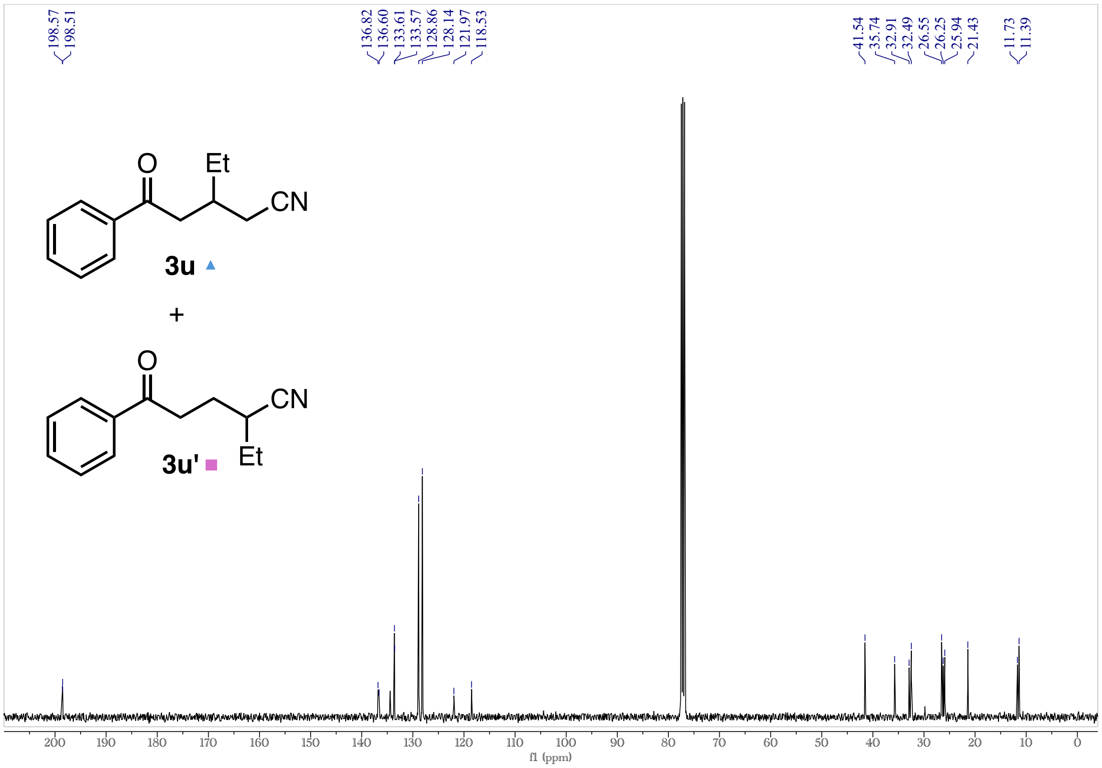

^{13}C{^{1}H} **NMR** (101 MHz, CDCl$_3$)

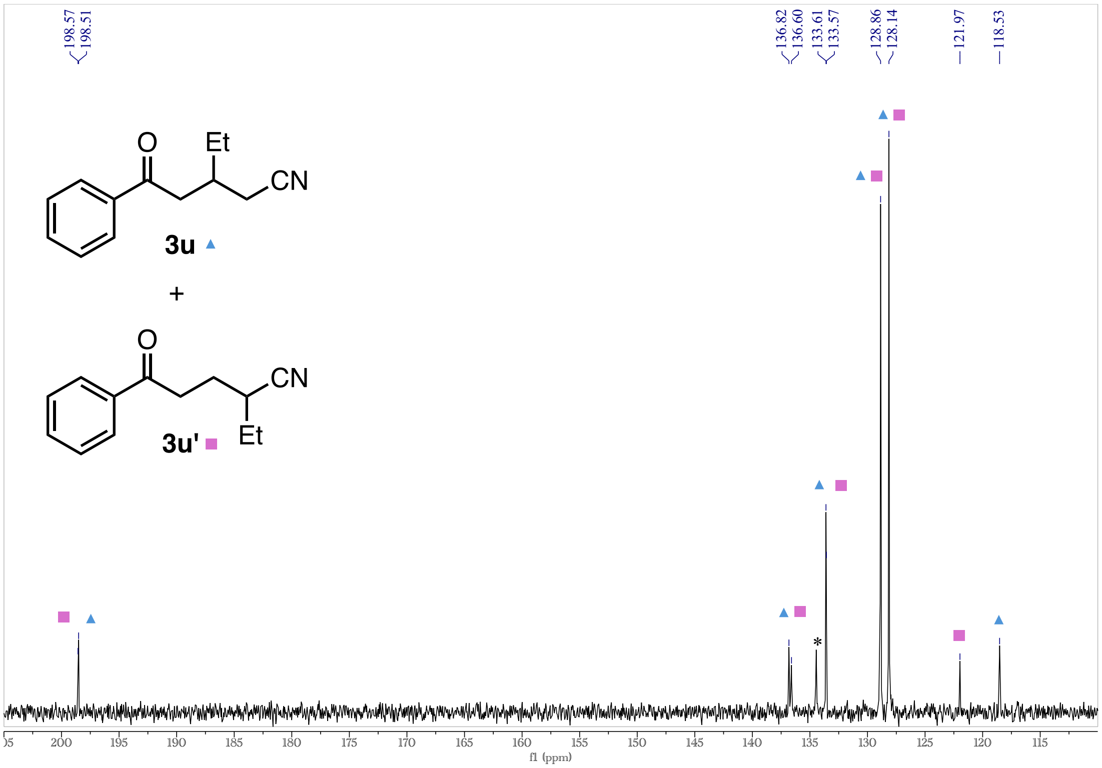

^{13}C{^{1}H} **NMR** (101 MHz, CDCl$_3$) aromatic region zoom

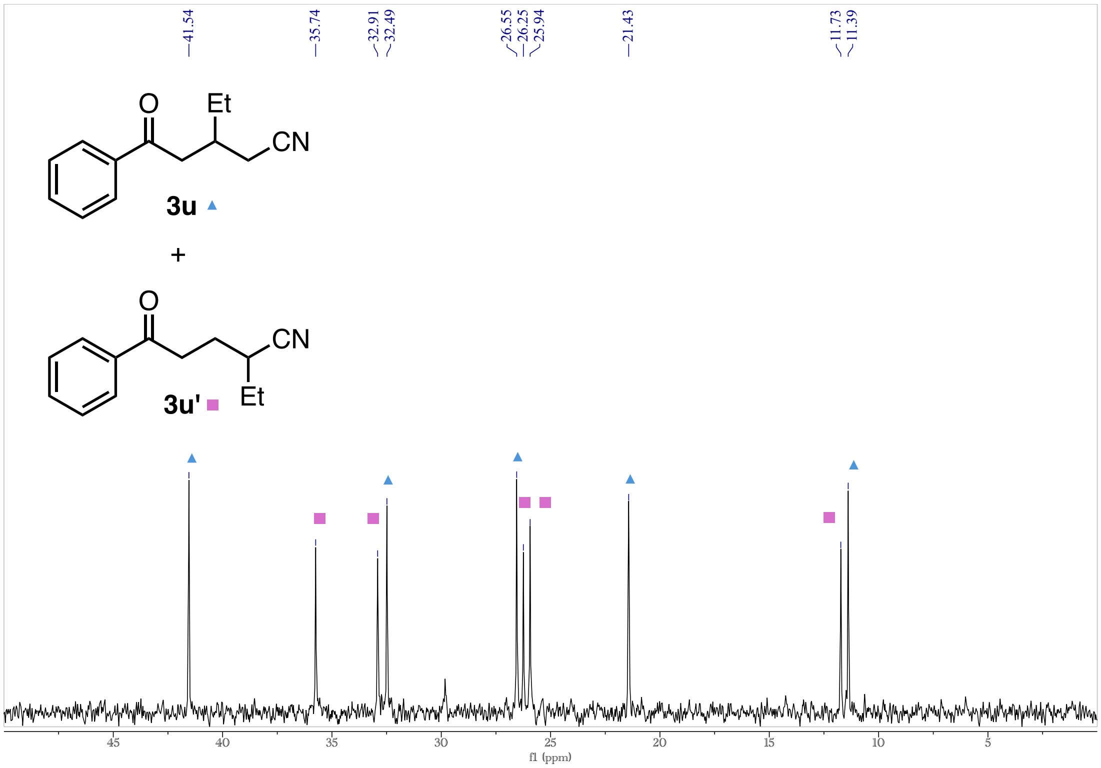

^{13}C{^{1}H} NMR (101 MHz, $CDCl_3$) alkyl region zoom

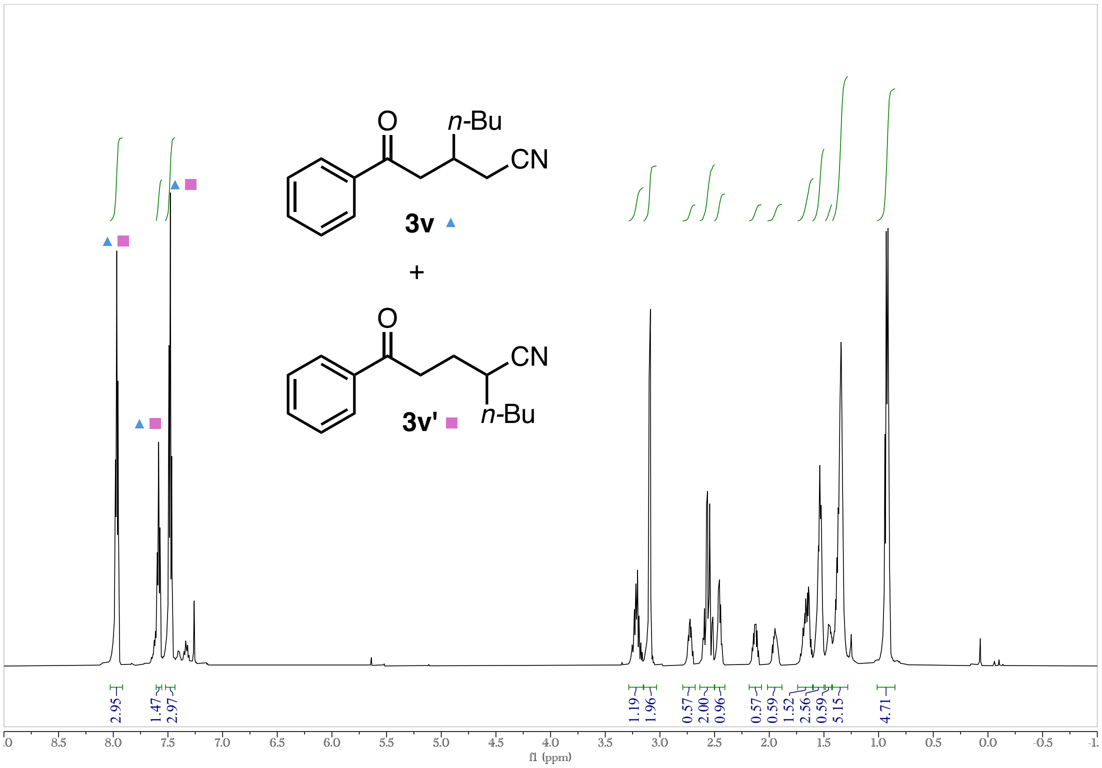

^{1}**H NMR** (600 MHz, $CDCl_3$)

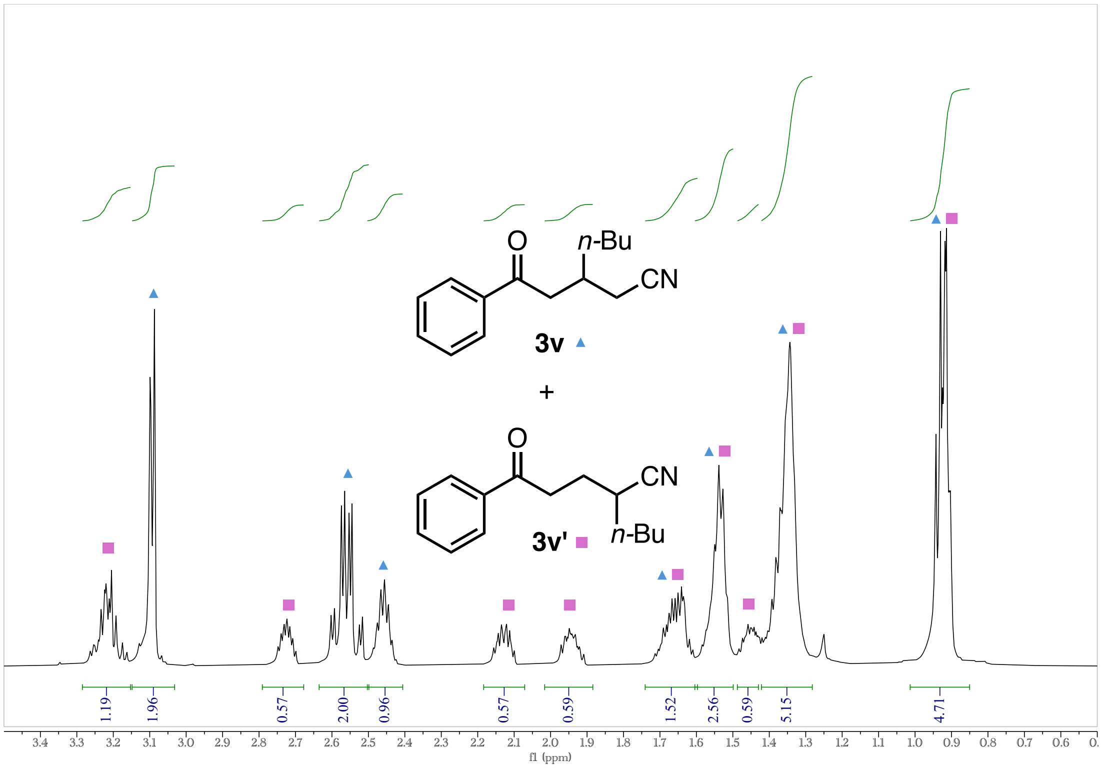

^{1}**H NMR** (600 MHz, $CDCl_3$) alkyl region zoom

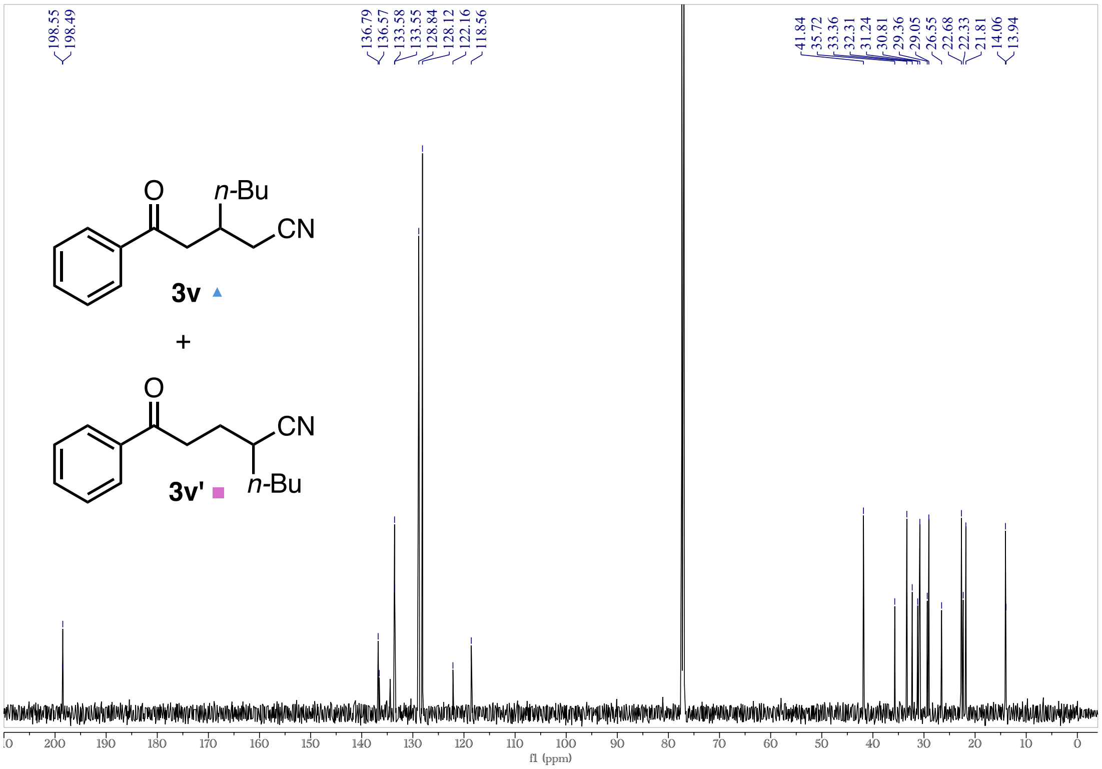

$^{13}C\{^1H\}$ NMR (151 MHz, $CDCl_3$)

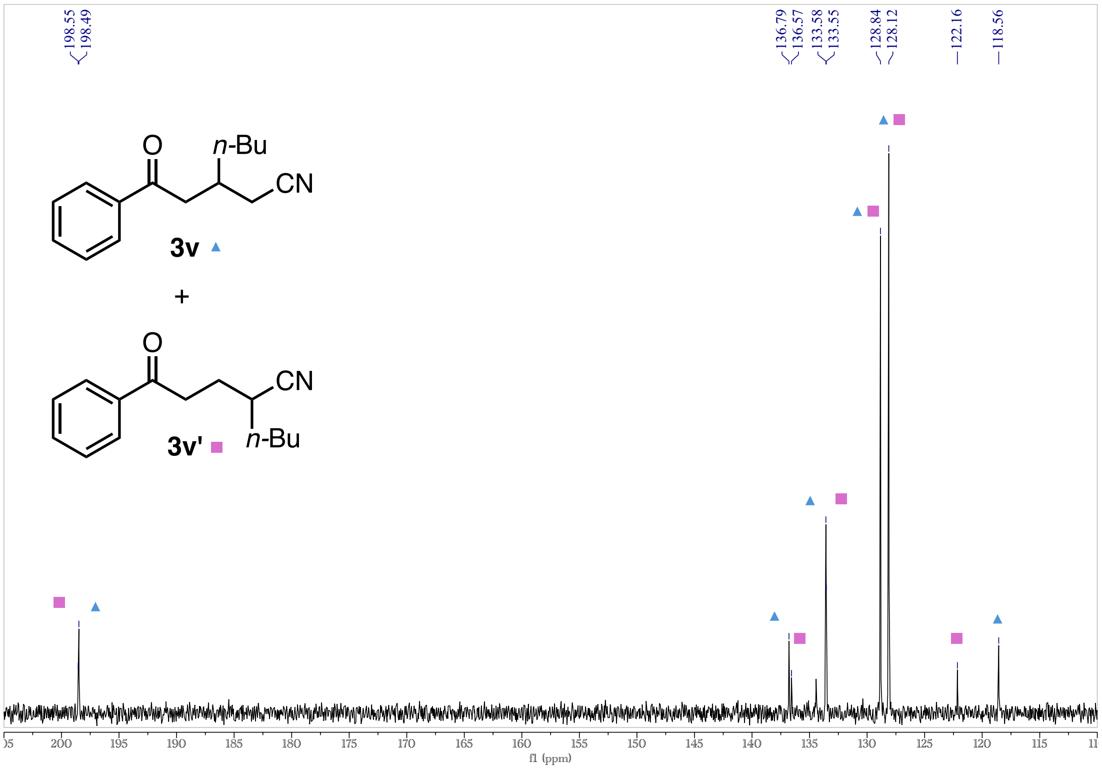

$^{13}C\{^1H\}$ NMR (151 MHz, $CDCl_3$) aromatic region zoom

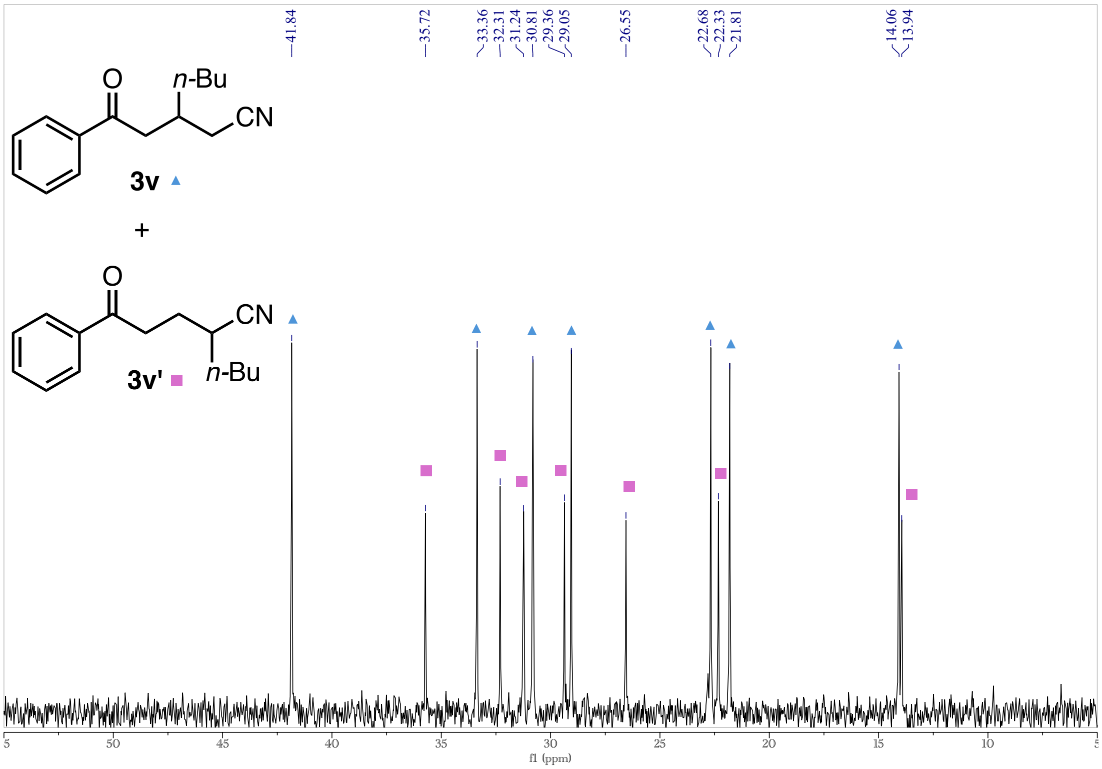

^{13}C{^{1}H} NMR (151 MHz, $CDCl_3$) alkyl region zoom

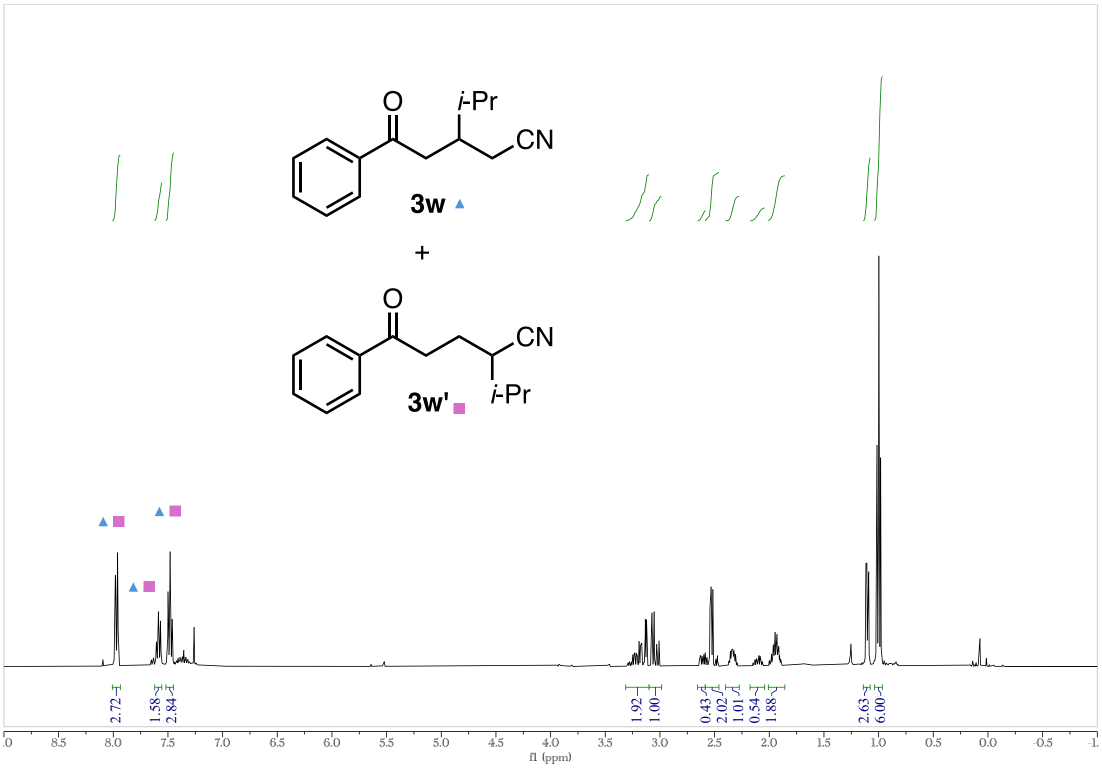

^{1}**H NMR** (400 MHz, $CDCl_3$)

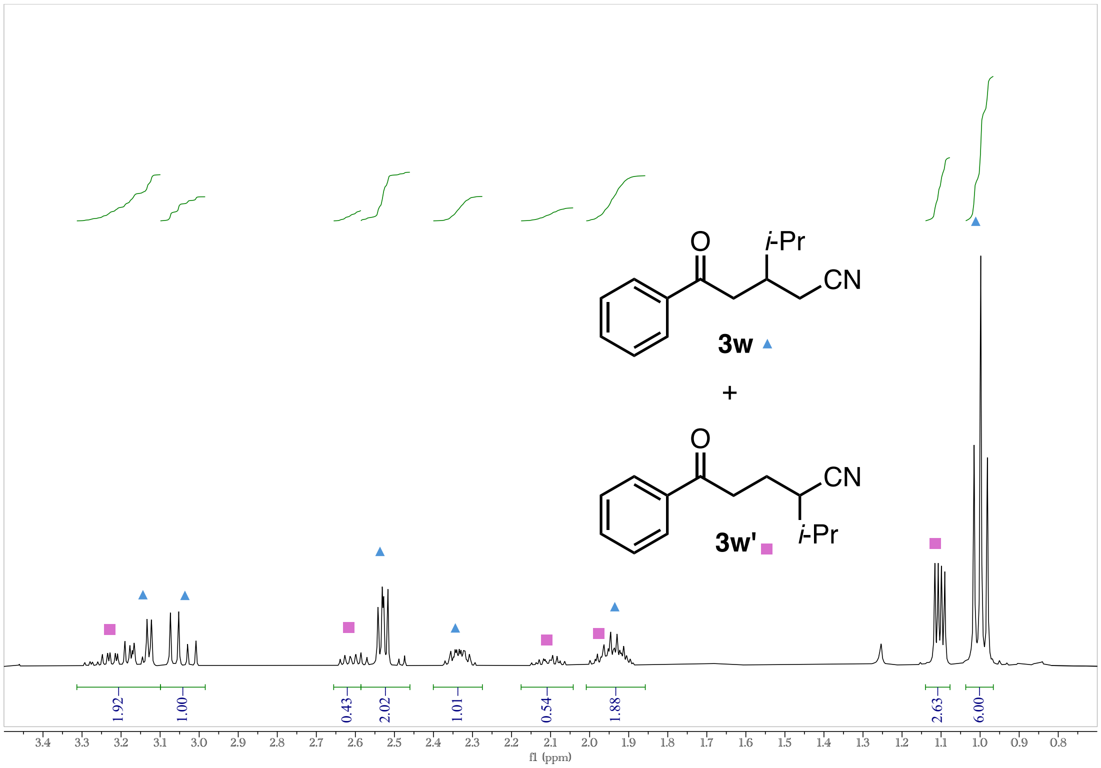

^{1}**H NMR** (400 MHz, $CDCl_3$) alkyl region zoom

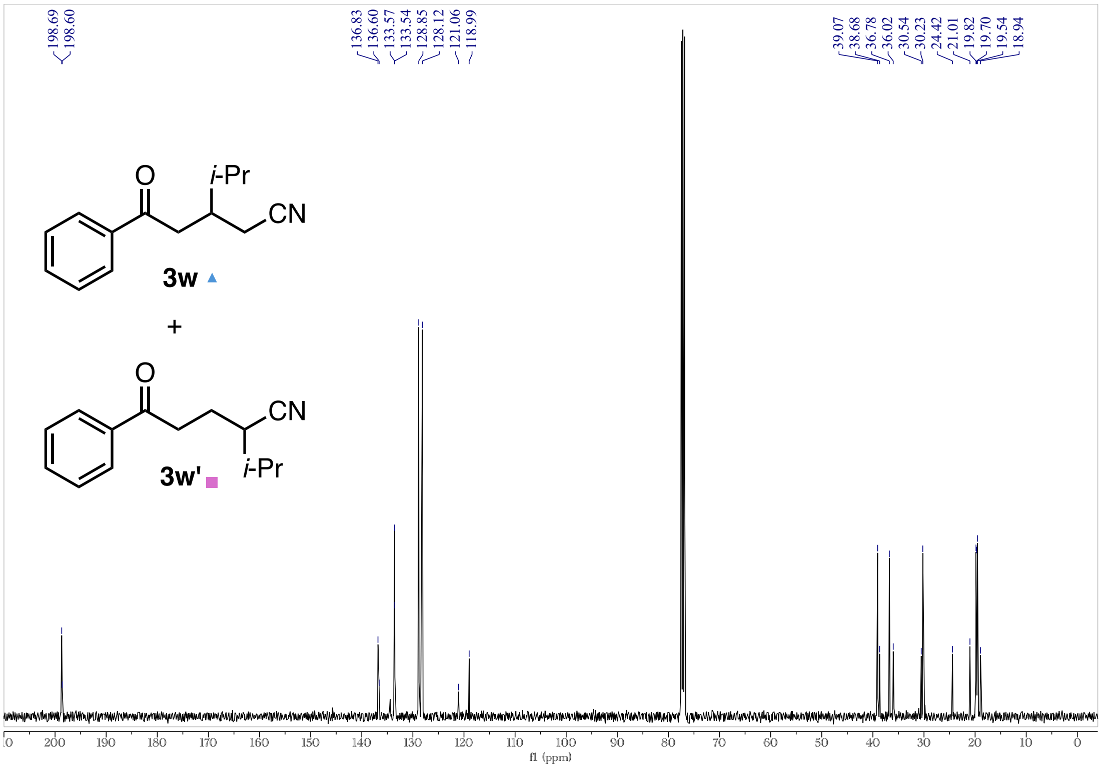

$^{13}C\{^1H\}$ NMR (101 MHz, $CDCl_3$)

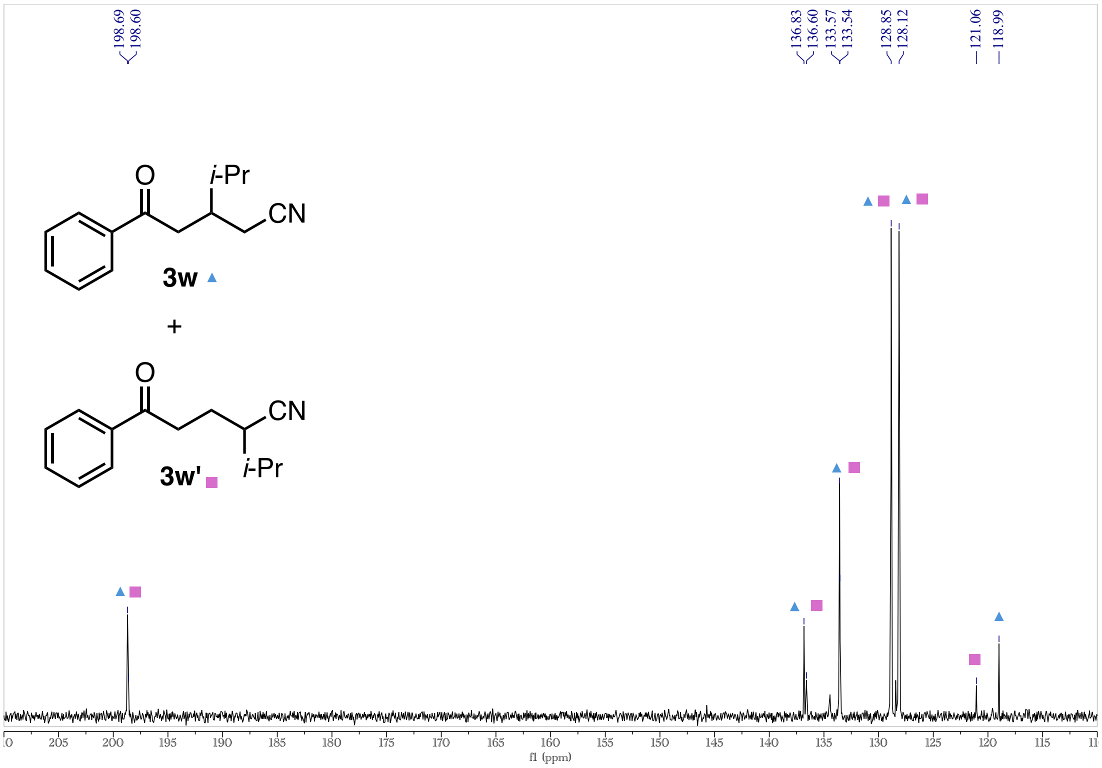

$^{13}C\{^1H\}$ NMR (101 MHz, $CDCl_3$) aromatic region zoom

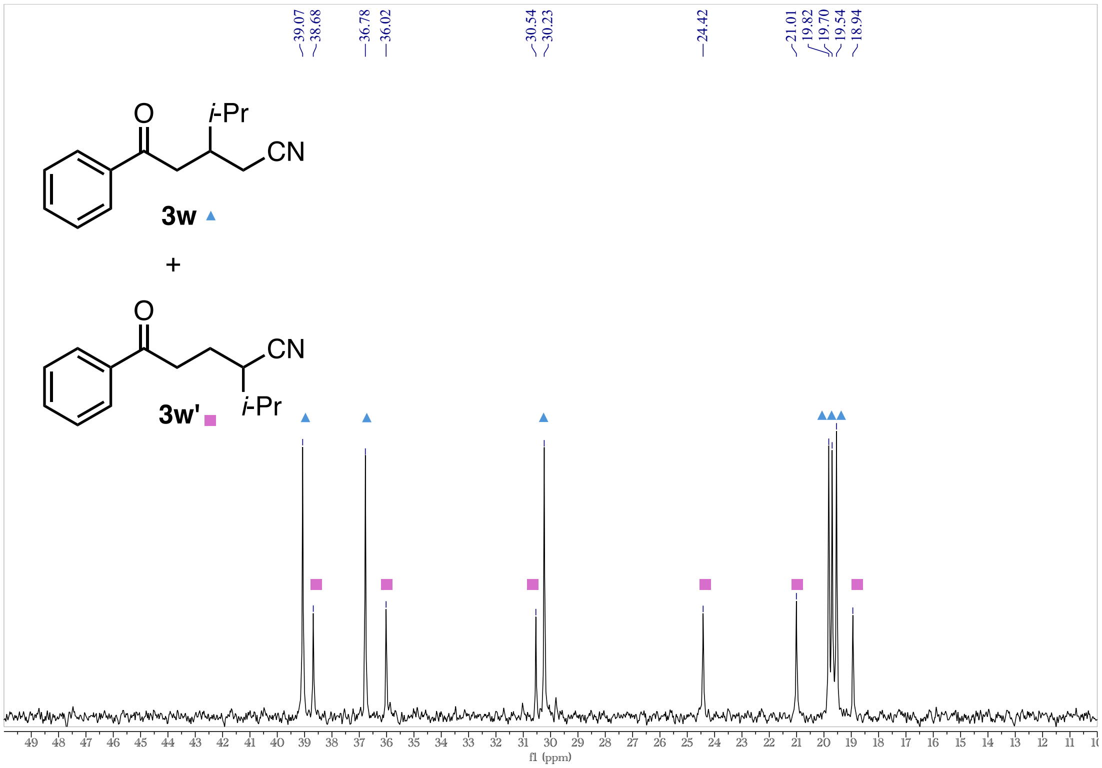

^{13}C{^{1}H} NMR (101 MHz, $CDCl_3$) alkyl region zoom

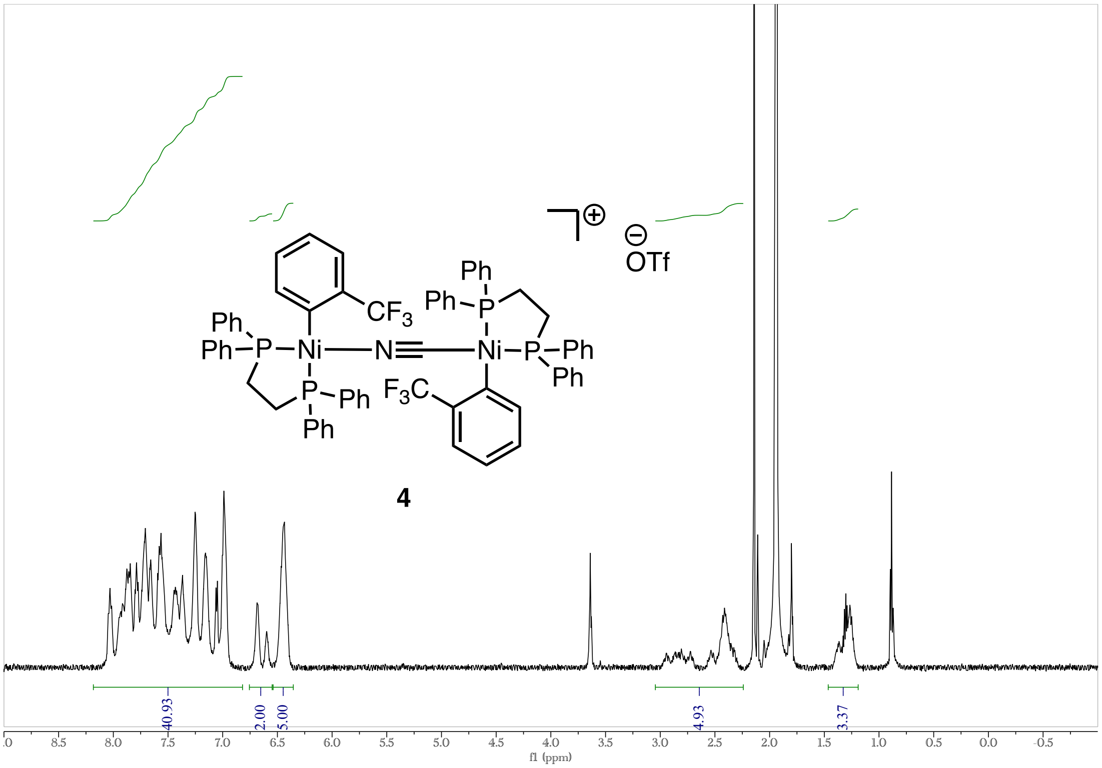

^{1}**H NMR** (600 MHz, CD_3CN)

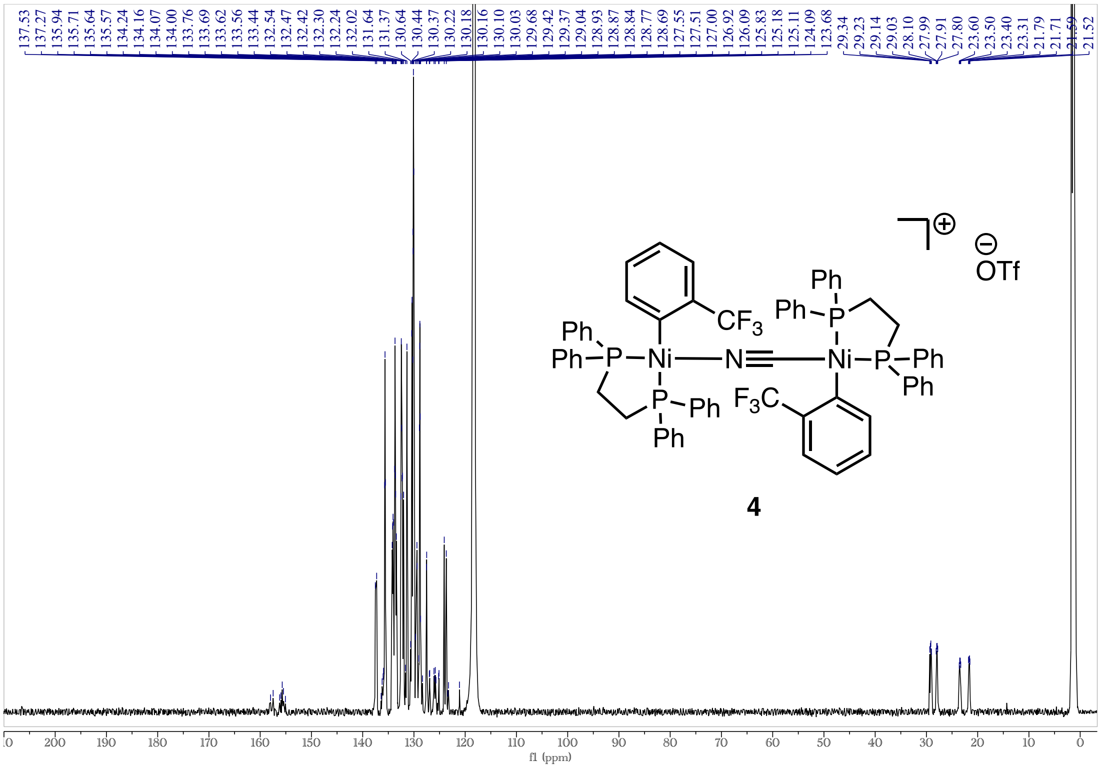

^{13}**C{^{1}H} NMR** (151 MHz, CD_3CN)

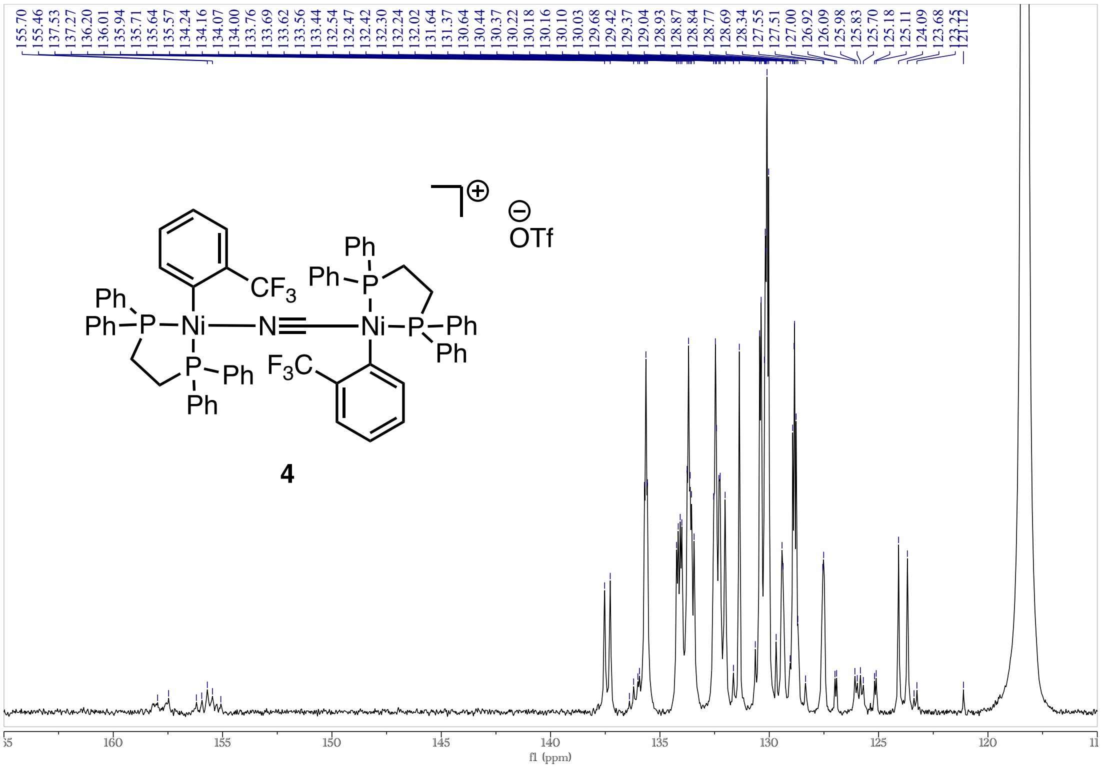

^{13}C{^{1}H} NMR (151 MHz, CD_3CN) aromatic region zoom

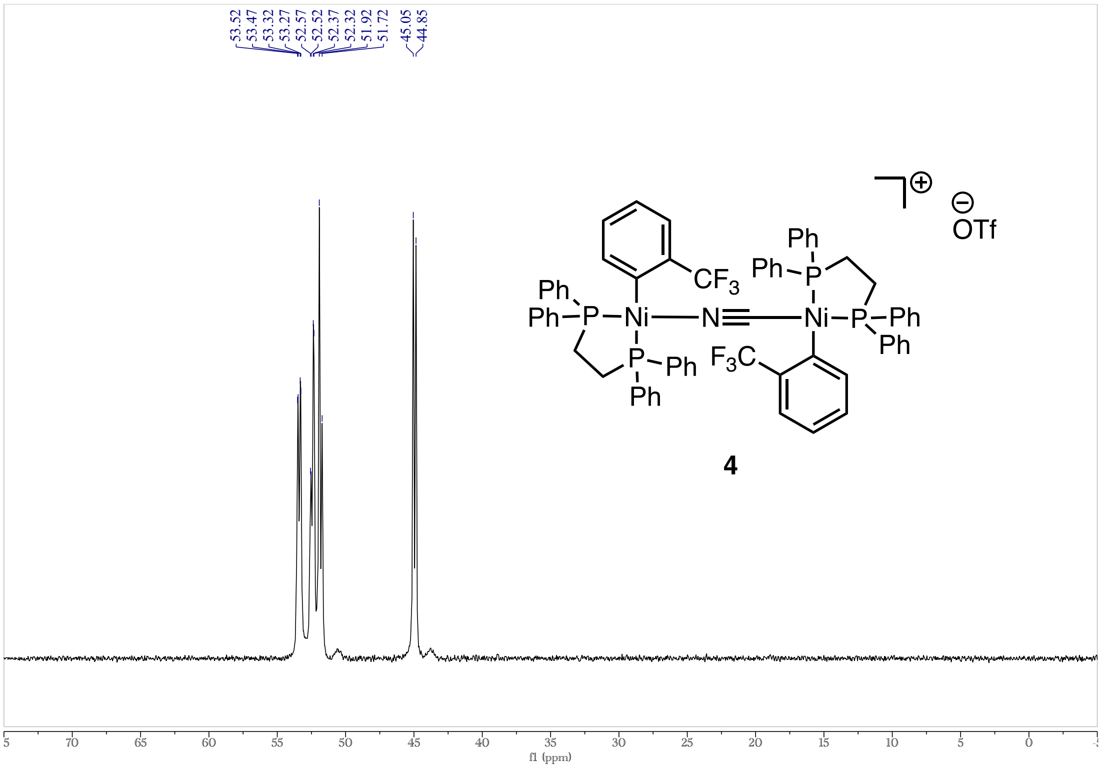

^{31}P NMR (162 MHz, CD_3CN)

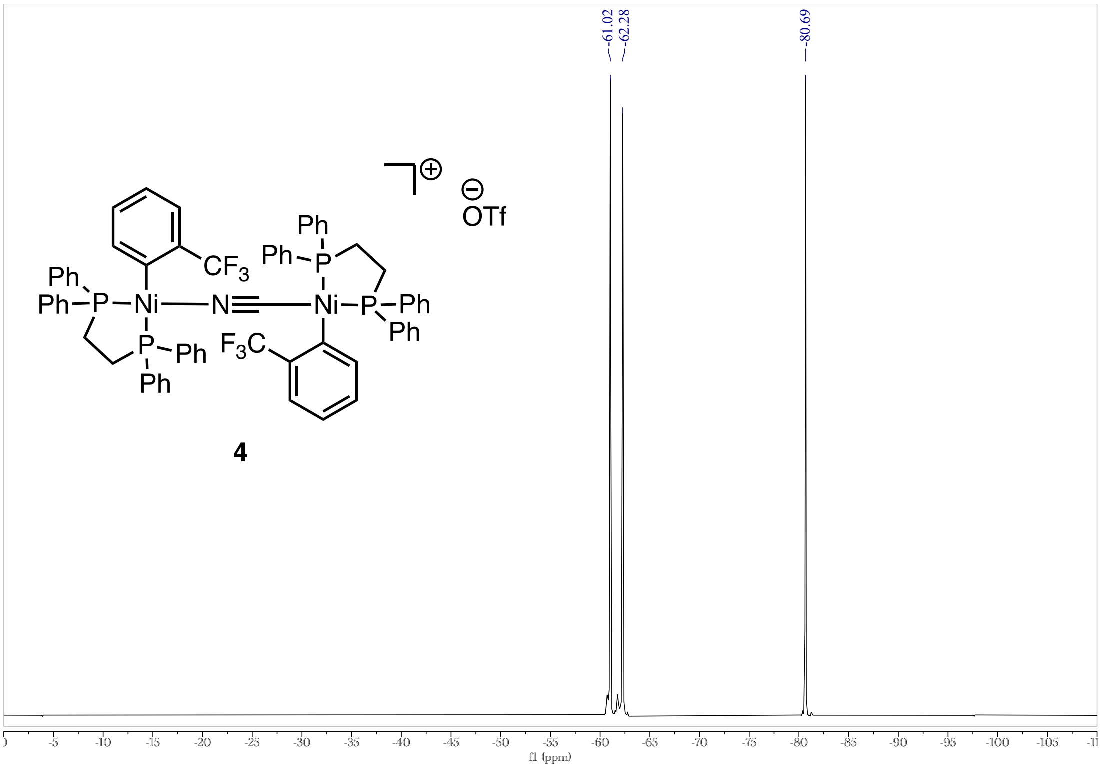

^{19}F NMR (376 MHz, CD_3CN)
